# METTL14 is required for exercise-induced cardiac hypertrophy and protects against myocardial ischemia-reperfusion injury

**DOI:** 10.1038/s41467-022-34434-y

**Published:** 2022-11-09

**Authors:** Lijun Wang, Jiaqi Wang, Pujiao Yu, Jingyi Feng, Gui-e Xu, Xuan Zhao, Tianhui Wang, H. Immo Lehmann, Guoping Li, Joost P. G. Sluijter, Junjie Xiao

**Affiliations:** 1 Cardiac Regeneration and Ageing Lab, Institute of Geriatrics (Shanghai University), Affiliated Nantong Hospital of Shanghai University (The Sixth People’s Hospital of Nantong), School of Medicine, Shanghai University, Nantong, 226011 China; 2 Institute of Cardiovascular Sciences, Shanghai Engineering Research Center of Organ Repair, School of Life Science, Shanghai University, Shanghai, 200444 China; 3 Department of Cardiology, Shanghai Tongji hospital, Tongji University School of Medicine, Shanghai, 200065 China; 4 Cardiovascular Division of the Massachusetts General Hospital and Harvard Medical School, Boston, MA 02114 USA; 5 Department of Cardiology, Laboratory of Experimental Cardiology, University Medical Center Utrecht, Utrecht, 3508GA The Netherlands; 6 Regenerative Medicine Center, Circulatory Health Laboratory, University Medical Center Utrecht, University Utrecht, Utrecht, 3508GA The Netherlands

**Keywords:** RNA modification, Cardiovascular diseases, Cardiovascular biology

## Abstract

RNA m^6^A modification is the most widely distributed RNA methylation and is closely related to various pathophysiological processes. Although the benefit of regular exercise on the heart has been well recognized, the role of RNA m^6^A in exercise training and exercise-induced physiological cardiac hypertrophy remains largely unknown. Here, we show that endurance exercise training leads to reduced cardiac mRNA m^6^A levels. METTL14 is downregulated by exercise, both at the level of RNA m^6^A and at the protein level. In vivo, wild-type METTL14 overexpression, but not MTase inactive mutant METTL14, blocks exercise-induced physiological cardiac hypertrophy. Cardiac-specific METTL14 knockdown attenuates acute ischemia-reperfusion injury as well as cardiac dysfunction in ischemia-reperfusion remodeling. Mechanistically, silencing METTL14 suppresses *Phlpp2* mRNA m^6^A modifications and activates Akt-S473, in turn regulating cardiomyocyte growth and apoptosis. Our data indicates that METTL14 plays an important role in maintaining cardiac homeostasis. METTL14 downregulation represents a promising therapeutic strategy to attenuate cardiac remodeling.

## Abstract (web summary)

The benefit of regular exercise on the heart has been well documented but the role of RNA m^6^A modification in exercise-induced cardiac remodelling remains largely unknown. In this study, the authors report that METTL14 plays an important role in RNA m^6^A modification in exercise-induced physiological cardiac hypertrophy.

## Introduction

Heart failure is a common cardiovascular disease that is marked by high morbidity and mortality^1^. Positively influencing cardiac remodeling to prevent occurrence of end-stage heart failure has been a mainstay of current therapeutic strategies. Regular exercise training benefits cardiac performance and induces physiological cardiac hypertrophy^2,3^. Previous studies have indicated, that key mediators of exercise-induced physiological cardiac hypertrophy may promote cardiomyocyte growth and reinforce resistance of cardiomyocytes against apoptosis, thereby protecting the heart from pathological remodeling^4–7^. N^6^-methyladenosine modification of RNA (RNA m^6^A) is a highly conserved and widely distributed modification in mammalian cells, playing an important role in post-transcriptional gene regulation^8^. RNA m^6^A has been found to be pertinent for cardiac homeostasis and as a response to pathological cardiac processes^9–14^. However, whether alteration of RNA m^6^A methylation is involved in exercise-induced physiological cardiac hypertrophy, and the role of RNA m^6^A methylation in exercise training remains largely unknown.

Modification of m^6^A is dynamically regulated by m^6^A methyltransferases (METTL3, METTL14, WTAP) and m^6^A demethylases (FTO, ALKBH5)^15^. METTL14, which forms a heterodimer of m^6^A methyltransferase with METTL3 to compose the core m^6^A methyltransferase complex, is required for the m^6^A modification substrate recognition and formation^16,17^. METTL14 has been found to play an important role in many diseases^18–22^, but the cardiac function of METTL14 remains unclear. Here, we found that endurance exercise led to reduced mRNA m^6^A methylation levels in murine hearts. METTL14 was downregulated by exercise training, both at the level of RNA m^6^A methylation, and at the protein level. We further explored the function and underlying mechanism of METTL14-mediated m^6^A modification in cardiomyocytes which suggested that the level of cardiac m^6^A methylation was increased by METTL14 overexpression. MTase inactive mutant METTL14 blunted the anti-hypertrophy effects of METTL14 overexpression on exercise-induced cardiac hypertrophy. Downregulation of METTL14 contributes to alleviation of acute myocardial ischemia-reperfusion (I/R) injury and cardiac dysfunction during I/R remodeling. Our data provides a fundamental understanding of METTL14 in cardiac remodeling and exhibits evidence that inhibition of METTL14 could be a therapeutic strategy.

## Results

### Endurance exercise training leads to reduced mRNA m^6^A methylation levels

**Fig. 1:**
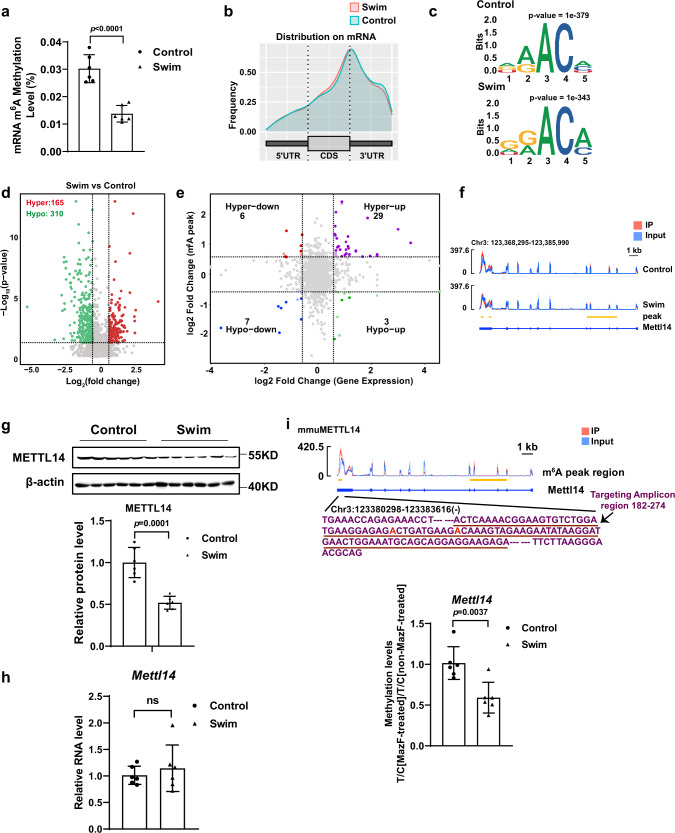
Endurance exercise training leads to a reduced mRNA m^6^A methylation level. **a** The global mRNA m^6^A level in swimming exercise-trained murine hearts (*n* = 6 /group). **b** Metagene profile demonstrating the distribution of m^6^A peaks across mRNA transcripts in the mouse hearts of the swim and control group. UTR, untranslated Region. CDS, coding sequence. **c** Sequence motif identified consensus motif within m^6^A peaks by MeRIP-seq in control and swim group, respectively. Binomial distribution test (two-sided). **d** Volcano plot of the m^6^A enrichment in mRNAs of swim and control murine hearts. m^6^A mRNAs with significantly hypermethylated peak (red) and hypomethylated peak (green) enrichment is highlighted (*p*-value < 0.05, fold change > 1.5). Negative binomial test (two-sided). **e** Correlation between the level of gene expression and changes in m^6^A modification levels in the swim and control hearts. **f** Integrative genomics viewer (IGV) tracks revealing the results of meRIP-seq (Red) and RNA-seq (Blue) reads distributions in *Mettl14* mRNA of the swim (*n* = 3 independent biological samples) and control mouse hearts (*n* = 4 independent biological samples). Plots are the medians of the *n* replicates presented. **g** Western blot analyses of METTL14 in whole lysates isolated from the hearts of swim and control murine (*n* = 6 mice/group). **h** qPCR analysis of *Mettl14* mRNA expression levels in the hearts with or without swim training (*n* = 6 mice/group). **i** The methylation levels of *Mettl14* mRNA in the swim and control hearts was measured by MazF-qPCR (*n* = 6 mice/group). The levels of a targeted amplicon (labeled “T”) is measured against a control (labeled “C”) amplicon in a MazF-digested sample and normalized against a non-digested sample. Seq, sequencing. IP, immunoprecipitation. All data are expressed as means ± SD. **a**, **g**, **h**, **i** Independent-sample *t*-test, two-sided. Source data are provided as a Source Data file.

The level of m^6^A modification in physiological cardiac hypertrophy was evaluated using an enzyme-linked immunosorbent assay (ELISA)-based m^6^A quantification assay. We quantified the global cardiac mRNA m^6^A level in a swimming exercise-induced physiological cardiac hypertrophy model and found that the global cardiac mRNA m^6^A level was significantly decreased upon exercise training (Fig. 1a). Subsequently, we performed m^6^A methylated RNA immunoprecipitation sequencing (meRIP-seq) in control and swimming exercise-induced physiological cardiac hypertrophy hearts (Supplementary Fig. 1a). Like previous meRIP-seq, our results revealed enrichment of m^6^A peaks on 3’- untranslated regions (3’-UTR) and stop codons (Fig. 1b, Supplementary Fig. 1b). The m^6^A peaks analyzed by HOMER^23^ demonstrated that DRACH (D = A, G, and U; R = G or A; H, non-guanine base) motif were enriched in control and swimming exercise-trained mouse hearts (Fig. 1c). RNA sequencing was used to determine a change in gene expression upon exercise training (Supplementary Fig. 1c). Both, the whole gene transcriptome and the m^6^A methylome were able to distinguish exercise status from sedentary control (Supplementary Fig. 1d). Differential m^6^A methylation peaks and statistical significance analyses were calculated by MeTDiff software^24^. MeRIP-seq demonstrated the presence of a total of 165 hypermethylated m^6^A peaks and 310 hypomethylated m^6^A peaks between control and exercised hearts (*p* –value < 0.05, fold change > 1.5) (Fig. 1d). Subsequently, RNA m^6^A peak data was plotted against the RNA-seq data, and RNA m^6^A modification levels were correlated with RNA expression levels. As shown in Fig. 1e, 10 hypo-methylated m^6^A peaks were identified of which the mRNA transcripts were significantly (*p* < 0.05; fold change > 1.5) downregulated (7; Hypo-down) or upregulated (3; Hypo-up). Thirty-five hyper-methylated m^6^A peaks were identified of which the mRNA transcripts were significantly (*p* < 0.05; fold change > 1.5) downregulated (6; Hyper-down) or upregulated (29; Hyper-up) in swimming exercise-trained hearts relative to the control group. Differential analysis of m^6^A sequencing data revealed 475 differentially methylated peaks in 436 genes (Supplementary Fig. 1e), indicating that RNA m^6^A which is involved in regulating of exercise-induced cardiac hypertrophy, has its effects at the level of post-transcriptional modification, rarely affecting mRNA abundance. In order to study RNA m^6^A alteration in response to exercise training, studies initially focused on known effectors of m^6^A modification and screening was performed based on three criteria: (1) Transcript not affected on the transcriptional level in response to exercise training, as demonstrated by no change in RNA-seq (swim vs. control); (2) Effectors that changed on the level of protein expression during exercise training (swim vs. control); (3) Transcript that differentially changed in response to exercise training, exhibited as differentially methylated m^6^A peaks in meRIP-seq (swim vs. control). Among these, METTL14 showed a significant decrease in response to swim training in meRIP-seq and at the protein level, while mRNA abundance of *Mettl14* demonstrated no change after exercise training (Fig. 1f–g and Supplementary Fig. 2). The mRNA abundance of *Mettl14* was further verified by qPCR and demonstrated no change after exercise training (Fig. 1h). Reduction of m^6^A methylation was further confirmed by MazF-qPCR (Fig. 1i), which is an antibody-independent method for mapping and measurement of m^6^A levels within mRNAs^25^. These data indicate that METTL14 is an exercise-induced cardiac m^6^A alteration response factor, which hence promotes us to pursue further investigation.

### Role of METTL14 on NRCMs growth and apoptosis

**Fig. 2:**
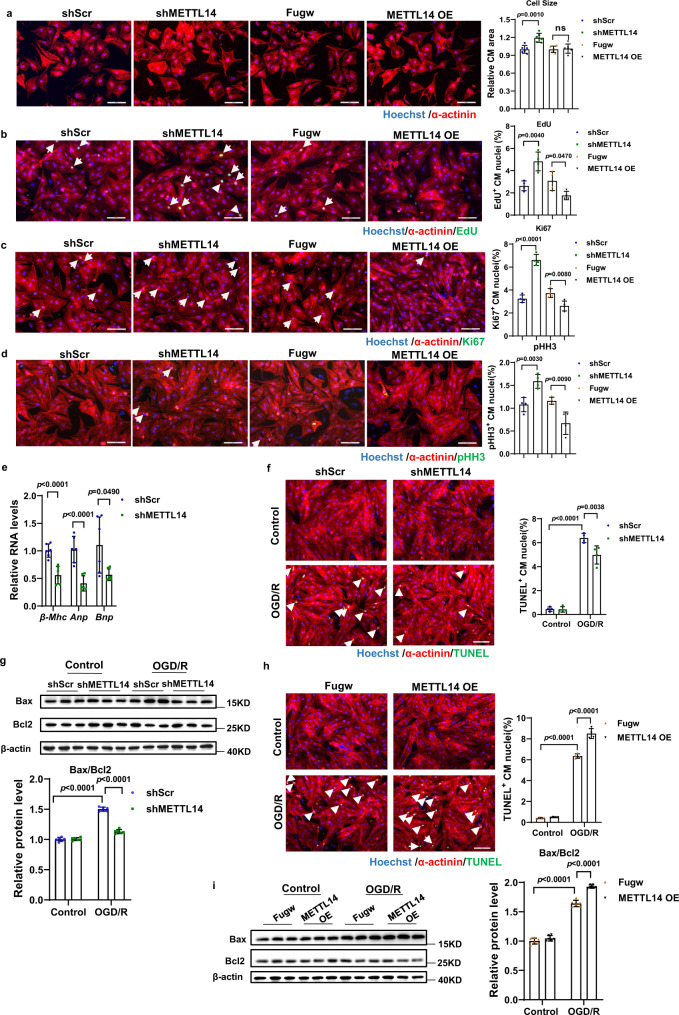
Role of METTL14 on NRCMs growth and apoptosis. **a** Representative images of immunofluorescence staining and quantification of the relative cardiomyocyte size treated with or without METTL14 overexpression or inhibition (*n* = 6 wells/group). Scale bar: 100 μm. **b** Representative images of immunofluorescence staining and quantification of the 5-ethynyl-2’-deoxyuridine (EdU) positive cardiomyocytes treated with or without METTL14 overexpression or inhibition (*n* = 4 wells/group). Scale bar: 100 μm. **c** Representative images of immunofluorescence staining and quantification of the Ki67 positive cardiomyocytes treated with or without METTL14 overexpression or inhibition (*n* = 4 wells/group). Scale bar: 100 μm. **d** Representative images of immunofluorescence staining and quantification of pHH3 positive cardiomyocytes treated with or without METTL14 overexpression or inhibition (*n* = 4 wells/group). Scale bar: 100 μm. **e** qPCR analysis of *Anp*, *Bnp*, *β-Mhc* mRNA expression levels in NRCMs treated with shMETTL14 or shScr (*n* = 6 wells /group). **f** Representative images of immunofluorescence staining and quantification of the TUNEL positive cardiomyocytes treated with shMETTL14 or shScr in OGD/R induced apoptosis model (*n* = 4 wells/group). Scale bar: 100 μm. **g** Representative western blot and statistical data of NRCMs apoptosis by detection of Bax, and Bcl2 in OGD/R induced apoptosis model treated with shMETTL14 or shScr (*n* = 6 wells/group). **h** Representative images of immunofluorescence staining and quantification of the TUNEL positive cardiomyocytes treated with METTL14 or Fugw in OGD/R induced apoptosis model (*n* = 4 wells/group). Scale bar: 100 μm. **i** Representative western blot and statistical data of NRCMs apoptosis by detection of Bax, and Bcl2 in OGD/R induced apoptosis model treated with METTL14 or Fugw (*n* = 6 wells/group). NRCM, neonatal rat cardiomyocyte. pHH3, phospho-Histone H3; shScr, Scramble short hairpin RNA; shMETTL14, METTL14 short hairpin RNA to knockdown METTL14; Fugw, control without METTL14 overexpression; METTL14 OE, METTL14 overexpression; TUNEL, terminal deoxynucleotidyl transferase dUTP nick end labeling; OGD/R, oxygen-glucose deprivation/reperfusion. All data are expressed as means ± SD. ns, nonstatistically significant. **a**–**e** Independent-sample *t*-test, two-sided; **f**–**i** two-way ANOVA followed by Tukey’s post hoc test. Source data are provided as a Source Data file.

To explore the role of METTL14 in cardiomyocytes, we performed gain- and loss- of function assays in primary neonatal rat cardiomyocytes (NRCMs). Overexpression and inhibition effects of METTL14 in NRCM was verified by western blot analyses (Supplementary Fig. 3). As shown in Fig. 2a, knockdown of METTL14 (shMETTL14) in NRCM resulted in an increase in basal cardiomyocyte size, while METTL14 overexpression (METTL14 OE) did not lead to a further decrease in size. Knockdown of METTL14 resulted in an increase in EdU incorporation (Fig. 2b), number of Ki67 positive NRCMs (Fig. 2c), as well as phosphor-histone H3 (pHH3) positive cells (Fig. 2d). Consistently, METTL14 OE led to a reduction of DNA synthesis (Fig. 2b), number of Ki67 positive cells (Fig. 2c), and number of positive pHH3 cells (Fig. 2d). Knockdown of METTL14 demonstrated a decrease in expression of heart failure markers *Anp*, *Bnp*, and *β-Mhc* mRNA (Fig. 2e). These data show that inhibition of METTL14 effectively promotes NRCM physiological hypertrophy, DNA synthesis, and a proportion of cardiomyocytes to enter into mitosis.

Next, we evaluated the effects of METTL14 on NRCM apoptosis in oxygen-glucose deprivation/reperfusion (OGD/R) treated NRCMs. As evidenced by TUNEL staining and western blotting (Bax/Bcl2), METTL14 knockdown demonstrated significant protective effects during OGD/R-induced NRCM apoptosis, while METTL14 overexpression led to aggravation of NRCM apoptosis (Fig. 2f–i). Therefore, these data reveal that METTL14 participates in the regulation of cardiomyocyte growth and apoptosis.

### METTL14 downregulation is necessary for exercise-induced cardiac hypertrophy

**Fig. 3:**
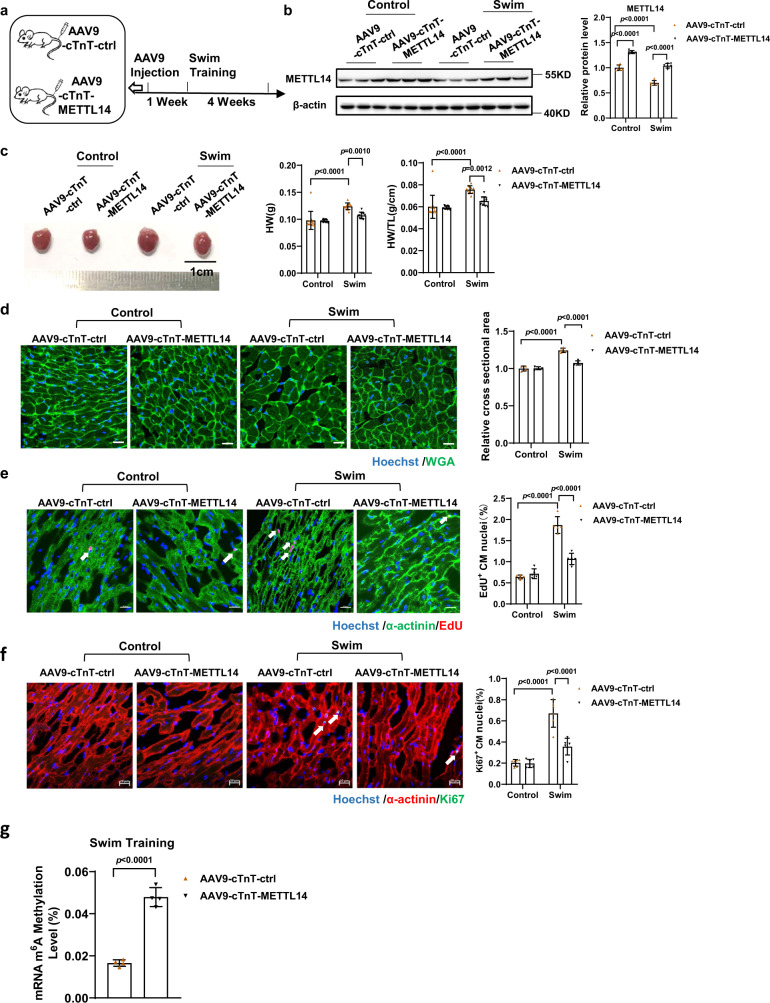
METTL14 downregulation is required for exercise-induced physiological cardiac hypertrophy. **a** Schedule of virus injection and swim-induced mice’s physiological cardiac hypertrophy model establishment. **b** Representative western blot and statistical data of METTL14 expression levels in mouse hearts treated as indicated (*n* = 6 mice/group). **c** Cardiac morphology, heart weight (HW), and heart weight/tibia length (HW/TL) in mouse hearts treated as indicated (*n* = 12, 12, 11 and 11 mice, respectively). **d** Representative images of Wheat Germ Agglutinin (WGA) staining and quantification of the relative cell cross-sectional area in mouse hearts treated as indicated (*n* = 4 mice/group). Scale bar: 20 μm. **e** Representative images of immunofluorescent staining and quantification of the cardiomyocytes 5-ethynyl-2’-deoxyuridine (EdU) positive ratio in mouse hearts treated as indicated (*n* = 5 mice/group). Scale bar: 20 μm. **f** Representative images of immunofluorescent staining and quantification of the cardiomyocytes Ki67 positive ratio in mouse hearts treated as indicated (*n* = 6 mice/group). Scale bar: 20 μm. **g** The relative mRNA m^6^A level in swimming-exercise treated murine hearts, infected with METTL14 overexpression or control AAV9 (*n* = 4/group). AAV9-cTnT-ctrl, cardiac-specific troponin-T promoter-driven control AAV9; AAV9-cTnT-METTL14, cardiac-specific troponin-T promoter-driven METTL14 overexpression AAV9. All data are expressed as means ± SD. **b**–**f** Two-way ANOVA followed by Tukey’s post hoc test; **g** independent-sample *t*-test, two-sided. Source data are provided as a Source Data file.

After our in vitro observations, we wondered whether METTL14 was required for exercise-induced cardiac growth. Mice were tail-vein injected with adeno-associated virus serotype 9 carrying a cardiac-specific troponin T promoter to drive METTL14 overexpression (AAV9-cTnT-METTL14), or with empty AAV9 control virus (AAV9-cTnT-ctrl) as controls. One week after tail-vein injection, mice were subjected to swim-training for 4 weeks, and hearts were collected for further analysis (Fig. 3a). Successful overexpression of METTL14 was confirmed by western blot (Fig. 3b), as well as the confirmed lowering of METTL14 levels upon exercise. As shown in Fig. 3c, trained mice that received an overexpression of METTL14 AAV9 demonstrated a significant decrease in heart weight (HW) and heart weight/tibia length (HW/TL) compared with trained mice that received the control injections (AAV9-cTnT-ctrl). Consistently, wheat germ agglutinin (WGA)-staining suggested that cell size decreased after METTL14 overexpression in swim-trained mice compared to control virus treated (Fig. 3d). In addition, the number of EdU-positive and Ki67-positive cardiomyocytes decreased after METTL14 overexpression in cardiac tissue of swim-trained mice (Fig. 3e, f). The global cardiac mRNA m^6^A level significantly decreased after normal exercise training (Fig. 1a). However, cardiac mRNA m^6^A modification levels in METTL14 overexpression animals were significantly increased compared to the control virus group upon swim training (Fig. 3g). Collectively, these data indicate that METTL14 overexpression partially abolishes exercise-induced cardiac hypertrophy.

### MTase inactive mutant METTL14 blunts anti-hypertrophy effects of METTL14 overexpression on exercise-induced cardiac hypertrophy

**Fig. 4:**
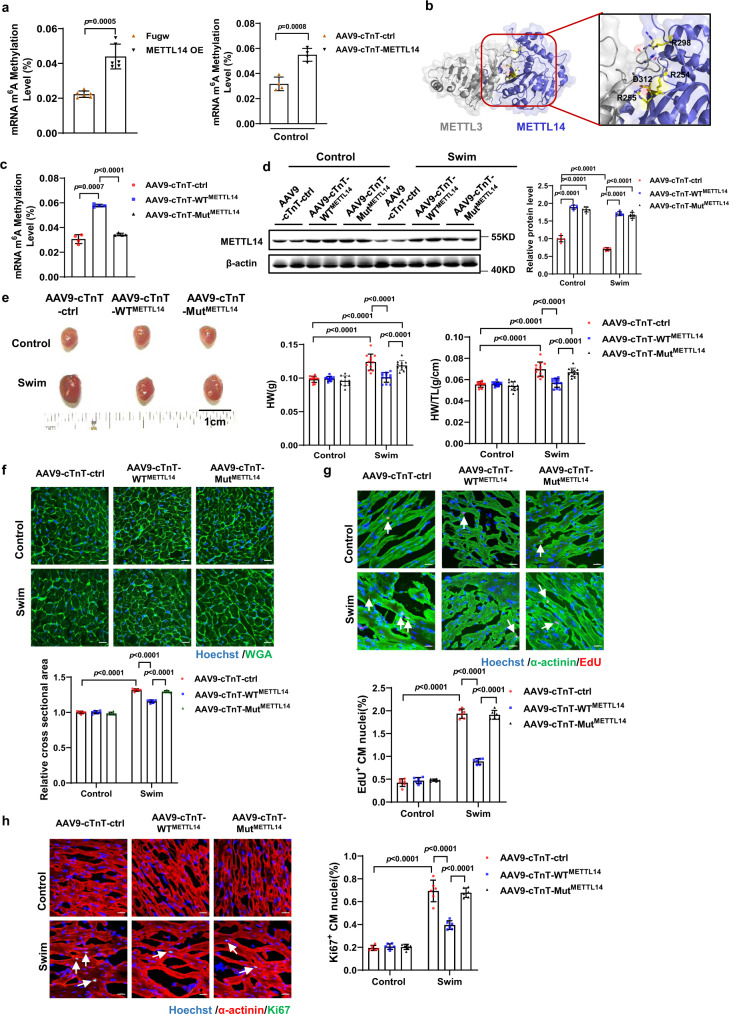
MTase inactive mutant METTL14 blunts the anti-hypertrophic effects of METTL14 overexpression on exercise-induced cardiac hypertrophy. **a** The relative mRNA m^6^A level infected with METTL14 overexpression in NRCM (*n* = 6/group) or mouse hearts (*n* = 4 /group). **b** Crystal structure of METTL3–METTL14 complex (PDB ID: 5IL0) showing the positively charged groove formed by METTL3-METTL14. Charged residues which are contributed by METTL14 are labeled as sticks. **c** The relative mRNA m^6^A level in hearts treated as indicated (*n* = 4/group). **d** Representative western blot and statistical data of METTL14 expression levels in mouse hearts treated as indicated (*n* = 6 mice/group). **e** Cardiac gross morphology, HW, and HW/TL in mouse hearts treated as indicated (*n* = 12 mice/group). **f** Representative images of WGA staining and quantification of the relative cell cross-sectional area in the mouse hearts treated as indicated (*n* = 6 mice/group). Scale bar: 20 μm. **g** Representative images of immunofluorescent staining and quantification of cardiomyocytes EdU positive ratio in the mouse hearts treated as indicated (*n* = 6 mice/group). Scale bar: 20 μm. **h** Representative images of immunofluorescent staining and quantification of the cardiomyocytes Ki67 positive ratio in the mouse hearts treated as indicated (*n* = 6 mice/group). Scale bar: 20 μm. NRCM, neonatal rat cardiomyocyte; Fugw, control without METTL14 overexpression; METTL14 OE, METTL14 overexpression; AAV9-cTnT-ctrl, cardiac-specific troponin-T promoter-driven control AAV9; AAV9-cTnT-METTL14, cardiac-specific troponin-T promoter-driven METTL14 overexpression AAV9; AAV9-cTnT-WT^METTL14^, cardiac-specific troponin-T promoter-driven wild-type METTL14 overexpression AAV9; AAV9-cTnT- Mut^METTL14^, cardiac-specific troponin-T promoter-driven METTL14 R254/R255A-R298P-D312A mutant overexpression AAV9; HW, heart weight. HW/TL, heart weight/tibia length. WGA, Wheat Germ Agglutinin. EdU, 5-ethynyl-2’-deoxyuridine. All data are expressed as means ± SD. **a** Independent-sample *t*-test, two-sided; **c** one-way ANOVA followed by Dunnett T3 test; **d**–**h** two-way ANOVA followed by Tukey’s post hoc test. Source data are provided as a Source Data file.

RNA m^6^A is added to mRNA by a multi-subunit “writer complex” composed of a METTL3-METTL14 heterodimer and adaptor proteins^16,26^. Previous reports suggest that METTL14 functions as a structural partner for m^6^A substrate recognition and activation of METTL3 via allosteric binding to function synergistically^17,27^. To examine whether the cardiac RNA m^6^A level change is associated with METTL14 function, we performed the experiments as follows. First, mRNA m^6^A levels were determined. The cardiac mRNA m^6^A level was associated with METTL14 alteration in neonatal cardiomyocytes and in mouse hearts (Fig. 4a and Supplementary Fig. 4). Second, a mutant of METTL14 was generated (Mut^METTL14^, METTL14 R254/R255A-R298P-D312A mutant); this has been reported to disrupt the positively charged groove that is formed by METTL3-METTL14 and dramatically abolishes MTase activity (Fig. 4b)^17,28,29^. Next, mice were tail-vein injected with a cTnT-driven AAV9 to drive WT^METTL14^ or Mut^METTL14^ overexpression. Seven days after injection, mice were subjected to 4-weeks of swim training (Supplementary Fig. 5a). Disruption of cardiac m^6^A level elevation in Mut^METTL14^ but not in WT^METTL14^ hearts was confirmed (Fig. 4c). Successful overexpression of WT^METTL14^ and Mut^METTL14^ was also confirmed by western blot (Fig. 4d). To distinguish WT^METTL14^ and Mut^METTL14^, primers for both WT and Mut^METTL14^, or specific for Mut^METTL14^ were designed and applied to RT-qPCR (Supplementary Fig. 5b). Swim-trained mice that received WT^METTL14^ AAV9 demonstrated a significant decrease in gross cardiac size, heart weight, and heart weight/tibia length, compared to trained mice that received Mut^METTL14^ and control AAV9 (Fig. 4e). WGA, EdU, and Ki67 staining verified that, unlike WT^METTL14^, Mut^METTL14^ AAV9 treatment did not lead to cell size alteration as well as a change in proportion of EdU-positive and Ki67-positive cardiomyocytes compared to AAV9-cTnT-ctrl group (Fig. 4f–h). Cardiac mRNA m^6^A level in WT^METTL14^, but not Mut^METTL14^ also increased compared with the control AAV9 treated group after swim training (Supplementary Fig. 5c). These data indicate, that abolishing the m^6^A methylation elevation effect via Mut^METTL14^ overexpression, leads to blunting of the antihypertrophic effects of METTL14 overexpression in mouse hearts that underwent swim training. Moreover, we performed functional assays in NRCM via Mut^METTL14^ (R234/235A-R278P-D292A^METTL14^) (Supplementary Fig. 6a). Abolished MTase activity by METTL14 mutants, was also verified by quantification of mRNA m^6^A levels in cardiomyocytes (Supplementary Fig. 6b). As demonstrated in Supplementary Fig. 6c, d, the pro-apoptotic and antiproliferative effects of WT^METTL14^ overexpression on NRCM were disrupted by Mut^METTL14^ overexpression. Thus, cardiac m^6^A methylation levels, which are increased by METTL14 overexpression, are required for the effects of METTL14 to blunt exercise-induced cardiac hypertrophy.

### Cardiac-specific knockdown of METTL14 alleviates acute I/R injury in mouse hearts

**Fig. 5:**
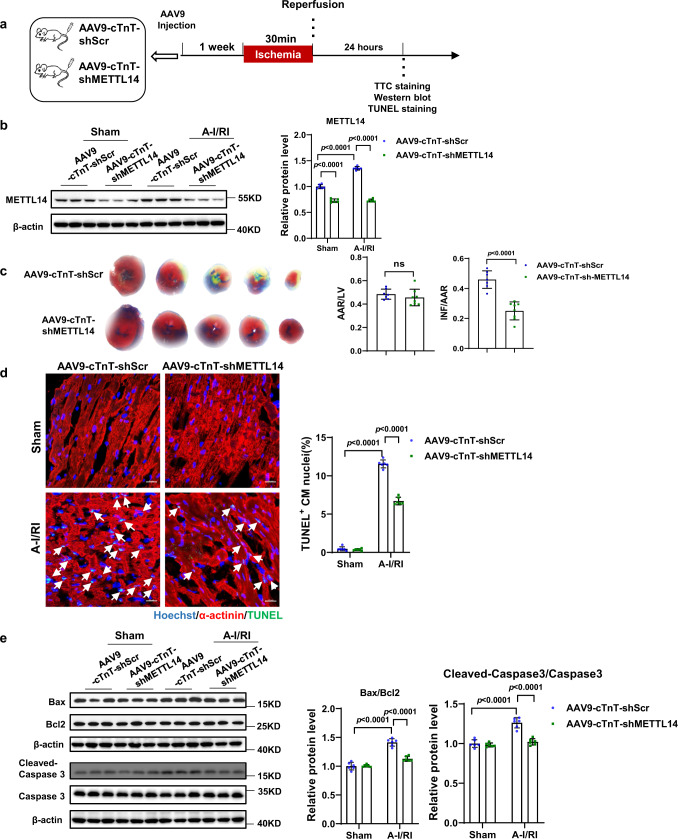
Cardiac-specific knockdown METTL14 alleviates acute myocardial I/R injury in mouse hearts. **a** Schedule of virus injection and acute myocardial I/R injury model establishment. **b** Representative western blot and statistical data of METTL14 expression levels in mouse hearts treated as indicated (*n* = 6 mice/group). **c** Representative images of 2,3,5-triphenyltetrazolium chloride (TTC) staining and quantification of the area at risk/left ventricular weight (AAR/LV) ratio and the infarct size/area at risk (INF/AAR) ratio (*n* = 7 and 8 mice, respectively). **d** Representative images of immunofluorescence staining and quantification of the TUNEL positive cardiomyocytes in mouse hearts treated as indicated (*n* = 6 mice/group). Scale bar: 20 μm. **e** Representative western blot and statistical data of myocardium apoptosis by detection of Bax, Bcl2, caspase 3, and Cleaved-Caspase 3 expression levels in mouse hearts treated as indicated (*n* = 6 mice/group). AAV9-cTnT-shScr, cardiac-specific troponin-T promoter-driven Scramble AAV9; AAV9-cTnT-shMETTL14, cardiac-specific troponin-T promoter-driven METTL14 knockdown AAV9; TUNEL, terminal deoxynucleotidyl transferase dUTP nick end labeling; ns, nonstatistically significant; I/R, ischemia-reperfusion. All data are expressed as means ± SD. **b**, **d**, and **e**, two-way ANOVA followed by Tukey’s post hoc test; **c**, independent-sample *t*-test, two-sided. Source data are provided as a Source Data file.

To further investigate the potential protective role of silencing METTL14 in the heart, we explored whether METTL14 knockdown protected against I/R injury in vivo. A miR-30d-based shRNA cassette was used in a cTnT promoter-driven METTL14 shRNA construct, thereby restricting shMETTL14 expression to cardiomyocytes^30^. Adult male mice received a tail vein injection of AAV9-cTnT-shMETTL14 or AAV9-cTnT-shScr that was subsequently followed by acute I/R injury (30 min/24 h) or sham surgery 1-week post AAV9 injection (Fig. 5a). Cardiac METTL14 downregulation was verified by western blotting upon AAV9-cTnT-shMETTL14 (Fig. 5b). Homogeneity of acute I/R injury was evaluated by calculation of the area at risk/left ventricular weight (AAR/LV) ratio in combination with TTC (2,3,5-triphenyltetrazolium chloride) staining. Upon shMETTL14 treatment, infarct size upon I/R injury was notably decreased (Fig. 5c). Furthermore, TUNEL (terminal deoxynucleotidyl transferase dUTP nick end labeling) staining and western blot analyses for apoptosis markers showed that silencing METTL14 protected the heart against acute I/R injury-induced cell apoptosis in vivo (Fig. 5d, e). Hence, downregulation of METTL14 alleviates acute I/R injury in mouse hearts.

### METTL14 inhibition alleviates cardiac dysfunction in I/R remodeling

**Fig. 6:**
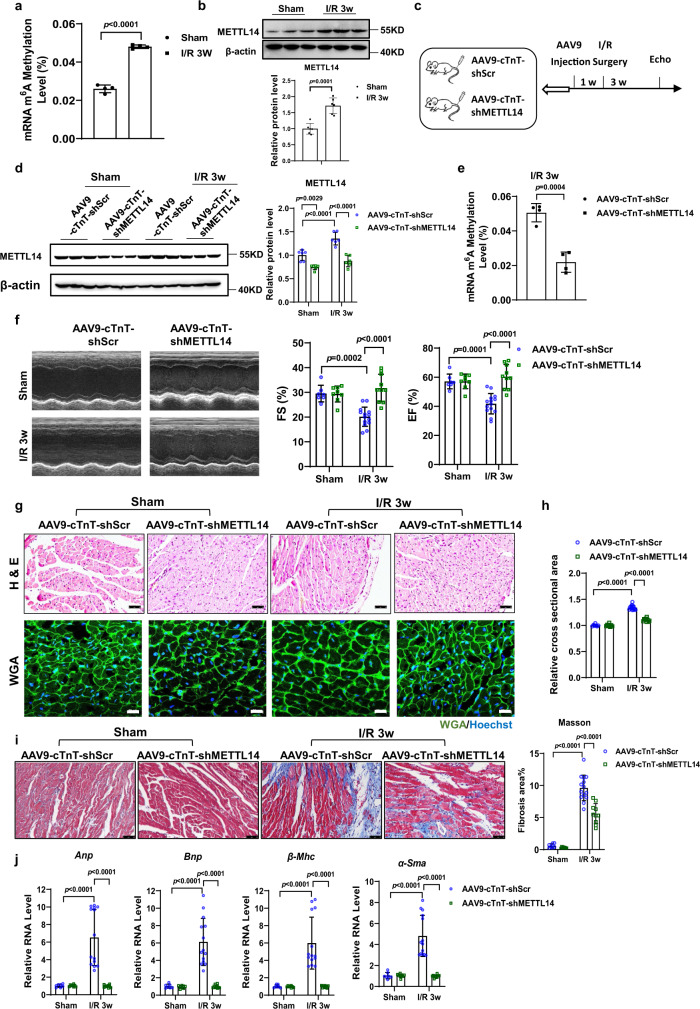
METTL14 knockdown could alleviate cardiac dysfunction in I/R remodeling. **a** The relative mRNA m^6^A level in murine hearts with or without I/R remodeling (*n* = 4/group). **b** Representative western blot and statistical data of METTL14 expression levels in the I/R 3w mouse hearts compared with sham surgery (*n* = 6 mice/group). **c** Schedule of virus injection and myocardial I/R injury-induced mouse pathological cardiac remodeling model establishment. **d** Representative western blot and statistical data of METTL14 expression levels in mouse hearts treated as indicated (*n* = 6 mice/group). **e** The relative mRNA m^6^A level in I/R 3w murine hearts infected with METTL14 knockdown or scrambled control AAV9 (*n* = 4/group). **f** Representative echocardiographic images and left ventricular ejection fraction (EF), ventricular fractional shortening (FS) of mice injected with AAV9-cTnT-shMETTL14 or scrambled control at day 21 after I/R surgery (*n* = 7, 8, 14, and 10 mice, respectively). **g** Representative images of H&E staining (Top, scale bar: 50 μm) and WGA staining (Bottom, scale bar: 20 μm). **h** Quantitative analysis of cross-sectional area of cardiac cells stained with WGA of ventricular myocardial cells in mouse hearts treated as indicated (*n* = 7, 8, 14, and 10 mice, respectively). **i** Representative images of Masson’s trichrome staining and quantification of fibrotic area (%) in mouse hearts treated as indicated (*n* = 7, 8, 14, and 10 mice, respectively). Scale bar: 50 μm. **j** qPCR analysis of *Anp*, *Bnp*, *β-Mhc*, and *α-Sma* mRNA levels in mouse hearts treated as indicated (*n* = 7, 8, 14, and 10 mice, respectively). I/R, ischemia-reperfusion. Echo, echocardiography. AAV9-cTnT-shScr, cardiac-specific troponin-T promoter-driven Scramble AAV9; AAV9-cTnT-shMETTL14, cardiac-specific troponin-T promoter-driven METTL14 knockdown AAV9; WGA, Wheat Germ Agglutinin; H&E, Hematoxylin & eosin. All data are expressed as means ± SD. **d**, **f**, **h**, **i,** **j** Two-way ANOVA followed by Tukey’s post hoc test; **a**, **b**, **e** independent-sample *t*-test, two-sided. Source data are provided as a Source Data file.

In addition to I/R responses, we also observed that the level of mRNA m^6^A modification and protein levels of METTL14 remained increased during myocardial I/R remodeling (Fig. 6a, b). This prompted us to investigate whether the protective effect of METTL14 downregulation exists to protect the heart from myocardial I/R remodeling. Therefore, we silenced METTL14 in mouse hearts through tail vein injection of an AAV9-cTnT-shMETTL14, which was followed by myocardial I/R or sham surgery 1 week post AAV9 injection (Fig. 6c). In addition, we validated knockdown of METTL14 and decrease of mRNA m^6^A modification levels in AAV9-cTnT-shMETTL14 treated mice (Fig. 6d, e), followed by functional follow-up *via* echocardiography 3 weeks after I/R induction. As shown in Fig. 6f, upon AAV9-cTnT-shMETTL14 treatment, cardiac function was preserved, as reflected by a maintained left ventricular ejection fraction (EF) and fractional shortening (FS) after I/R remodeling. Hematoxylin & eosin (H&E) and WGA staining revealed that development of enlarged ventricular myocardial cells during I/R remodeling was prevented in METTL14 knockdown mice (Fig. 6g, h). Furthermore, downregulation of METTL14 alleviated cardiac fibrosis during I/R remodeling, as evidenced by Masson trichrome staining (Fig. 6i) and prevented increased expression of heart failure markers *Anp*, *Bnp*, *β-Mhc*, and *α-Sma* on mRNA levels (Fig. 6j). Overall, these results suggested that silencing of METTL14 protects the heart from myocardial I/R remodeling.

### METTL14 knockdown inhibits *Phlpp2* mRNA m^6^A modification and activates Akt-S473

**Fig. 7:**
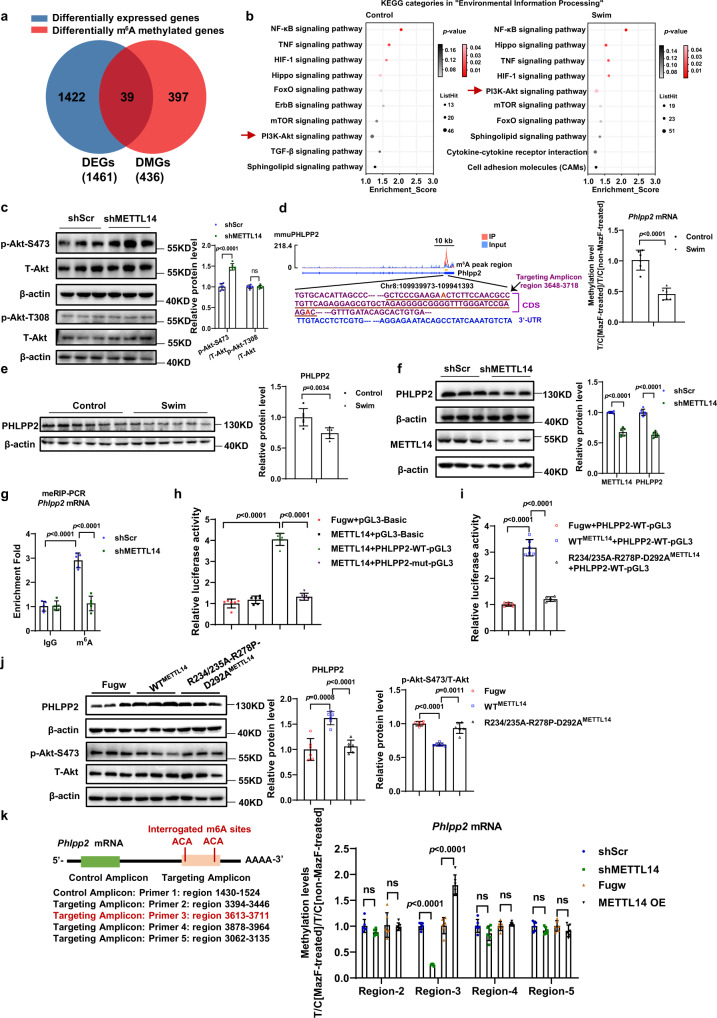
METTL14 knockdown inhibits Phlpp2 mRNA m^6^A modifications and activates Akt-S473. **a** Venn diagram showing the comparison of differentially methylated transcripts and differentially expressed transcripts identified in the exercise-induced cardiac hypertrophy murine model. DMG, differentially m^6^A methylated genes. DEG, differentially expressed genes. **b** KEGG analysis of significantly enriched m^6^A methylated peaks (*p* < 0.05; fold-change > 2.0) in control group and swim group, respectively. **c** Representative western blot and statistical data of phosphorylation levels of Akt-S473, Akt-T308 in NRCMs with or without METTL14 knockdown (*n* = 6 wells/group). **d** The methylation levels of *Phlpp2* mRNA in swim and control mouse hearts measured by MazF-qPCR (*n* = 6 mice/group). The levels of a targeted amplicon (labeled “T”) is measured against a control (labeled “C”) amplicon in a MazF-digested sample and normalized against a nondigested sample. **e** Western blot analyses of PHLPP2 in whole lysates isolated from swim and control murine hearts (*n* = 6 mice/group). **f** Western blot analyses of PHLPP2 and METTL14 in NRCM with or without METTL14 inhibition (*n* = 6 wells/group). **g** m^6^A enrichment levels in *Phlpp2* mRNA in H9C2 cardiomyocytes treated as indicated using m^6^A-RIP (meRIP)-qPCR (*n* = 5/group). **h** Relative luciferase activity of *Rattus* PHLPP2 wild-type (PHLPP2-WT-pGL3) or m^6^A mutant (PHLPP2-mut-pGL3) reporter gene with or without *Rattus* METTL14 overexpression (*n* = 6/group). **i** Relative luciferase activity of *Rattus* PHLPP2-WT-pGL3 reporter gene with *Rattus* wild-type METTL14 (WT^METTL14^) or mutant METTL14 (R234/235A-R278P-D292A^METTL14^) overexpression (*n* = 6/group). **j** Representative western blot and statistical data of PHLPP2 and phosphorylation level of Akt-S473 in NRCMs with WT^METTL14^ or R234/235A-R278P-D292A^METTL14^ overexpression (*n* = 6/group). **k** MazF-qPCR identified the methylation modification region of *Phlpp2* mRNA which is specifically regulated by METTL14 (*n* = 6/group). The levels of a targeted amplicon (labeled “T”) is measured against a control (labeled “C”) amplicon in a MazF-digested sample and normalized against a nondigested sample. NRCM, neonatal rat cardiomyocyte; shScr, Scramble short hairpin RNA; shMETTL14, METTL14 short hairpin RNA to knockdown METTL14. ns, nonstatistically significant. All data are expressed as means ± SD. **c**–**f**, **k** Independent-sample *t*-test, two-sided; **g** two-way ANOVA followed by Tukey’s post hoc test; **h**, **j** one-way ANOVA followed by Bonferroni test; **i** one-way ANOVA followed by Dunnett T3 test.) Source data are provided as a Source Data file.

Upon exercise stimulation, we observed 1461 differentially expressed genes (DEGs) and 436 differentially m^6^A methylated genes (DMGs). Interestingly, most of the differentially methylated genes (397/436) did not overlap with the differentially expressed genes (1422/1461), suggesting that altered post-transcriptional RNA m^6^A methylation in response to exercise is different from altered transcript levels (Fig. 7a). By comparing results of a KEGG analysis of significant enriched m^6^A methylated peaks (*p* < 0.05; fold-change > 2.0) between the control group and the swim group, we found that the PI3K-Akt signaling pathway was significantly enriched in the swim group (*p*-value < 0.05), while no significant difference was demonstrated for the control group (*p*-value > 0.05) (Fig. 7b). In addition, the PI3K-Akt pathway was the only signaling pathway that was significantly influenced by swim training. Of note, the PI3K-Akt is the major and classic signaling pathway involved in exercise-induced cardiac hypertrophy^2^. Upon these observations, we hypothesized that exercise-induced changes in Akt signaling are mediated *via* downregulation of METTL14. Therefore, we detected the phosphorylation level of Akt upon knockdown of METTL14. As shown in Fig. 7c, the phosphorylation level of Akt-T308 is not changed, while the phosphorylation level of Akt-S473 is increased, suggesting that the activation of Akt-S473 is regulated by METTL14. Activation of Akt-S473 was also present in OGD/R treated-NRCM upon METTL14 knockdown (Supplementary Fig. 7).

To further determine the mechanism that mediates increased Akt activation upon reduced METTL14 expression, we examined pleckstrin homology (PH) domain leucine-rich repeat protein phosphatase 2 (PHLPP2), a phosphatase regulating Akt-S473 phosphorylation^31^. The RNA m^6^A modification level and protein level of PHLPP2 are both downregulated in the hearts of swim-trained mice (Fig. 7d, e), which is consistent with activated Akt signaling after exercise training. In vitro, increase of the PHLPP2 protein levels upon METTL14 overexpression and a decrease in response to METTL14 knockdown were observed in NRCM (Fig. 7f, and Supplementary Fig. 8a). Also, the binding of METTL14 to *Phlpp2* mRNA was confirmed by METTL14-RIP assay (Supplementary Fig. 8b). The mRNA expression level of *Phlpp2* was not changed in swimming mouse hearts and in METTL14 alteration (Supplementary Fig. 8c). Consistently, the mRNA expression level of *Phlpp2* was not changed in the swimming mouse hearts (Supplementary Fig. 8d). YTHDF1 is the reader protein that has been reported to be involved in promotion translation efficiency of mRNA^32^. YTHDF1 RIP assay suggested that the binding of *Phlpp2* mRNA to YTHDF1 dramatically decreased after METTL14 knockdown, which is consistent with the decreased PHLPP2 protein level after METTL14 knockdown (Supplementary Fig. 8e). These data suggest that YTHDF1 was involved in recognition of METTL14 to PHLPP2.

To determine whether the effect of METTL14 on PHLPP2 expression is via affecting the *Phlpp2* mRNA m^6^A level, the presence of m^6^A modifications in *Phlpp2* and decreased m^6^A levels of *Phlpp2* mRNA after METTL14 knockdown were further verified by meRIP-PCR in cardiomyocytes (Fig. 7g). Accordingly, the m^6^A deposition in *Phlpp2* was increased in mouse hearts with METTL14 overexpression as confirmed by MazF-qPCR (Supplementary Fig. 8f). We then generated luciferase reporter gene that integrated the m^6^A peak region with WT or mutated m^6^A nucleotides (scheme of WT-PHLPP2 or Mut-PHLPP2 luciferase reporter gene shown in Supplementary Fig. 8g). In the PHLPP2 mutant pGL3 (PHLPP2-mut-pGL3) construct, the adenine, which located in the m^6^A peak region and within the m^6^A consensus sequences presented by RMBase v2.0^33^, were mutated to thymine. Luciferase assay suggested that the luciferase activity in PHLPP2-WT-pGL3 was significantly promoted by METTL14 overexpression, while the luciferase activity in PHLPP2-mut-pGL3 was not changed by METTL14 overexpression (Fig. 7h and Supplementary Fig. 8h). Next, we performed luciferase assay via transfecting WT^METTL14^, or R234/235A-R278P-D293A^METTL14^ mutant together with PHLPP2-WT-pGL3. R234/235A-R278P-D293A^METTL14^ mutant was generated based on previously reported point mutants on METTL14 that could abrogate the MTase activity^28,29^. Luciferase assay showed that, in contrast to WT^METTL14^, overexpression of the R234/235A-R278P-D293A^METTL14^ mutant did not promote the luciferase activity in PHLPP2-WT-pGL3(Fig. 7i). In addition, the protein level of PHLPP2 and phosphorylation level of Akt-S473 were not affected by the METTL14 mutant in cardiomyocytes (Fig. 7j). Moreover, we selected 4 regions that have been reported to have potential m^6^A modification sites in *Rattus Phlpp2* mRNA through searching the RNA Modification database^33^, and conducted the MazF-qPCR to verify which regions of *Phlpp2* mRNA were altered by METTL14. As shown in Fig. 7k and Supplementary Fig. 8i, the mRNA region of *Phppp2* ranging nucleotides 3613–3711 were influenced by METTL14, indicating that m^6^A modification of this conserved region was affected by METTL14. In summary, METTL14 inhibition regulates the *Phlpp2* mRNA m^6^A methylation level to suppress PHLPP2 expression, and activate Akt-S473.

### METTL14 regulates NRCMs growth and apoptosis via PHLPP2

**Fig. 8:**
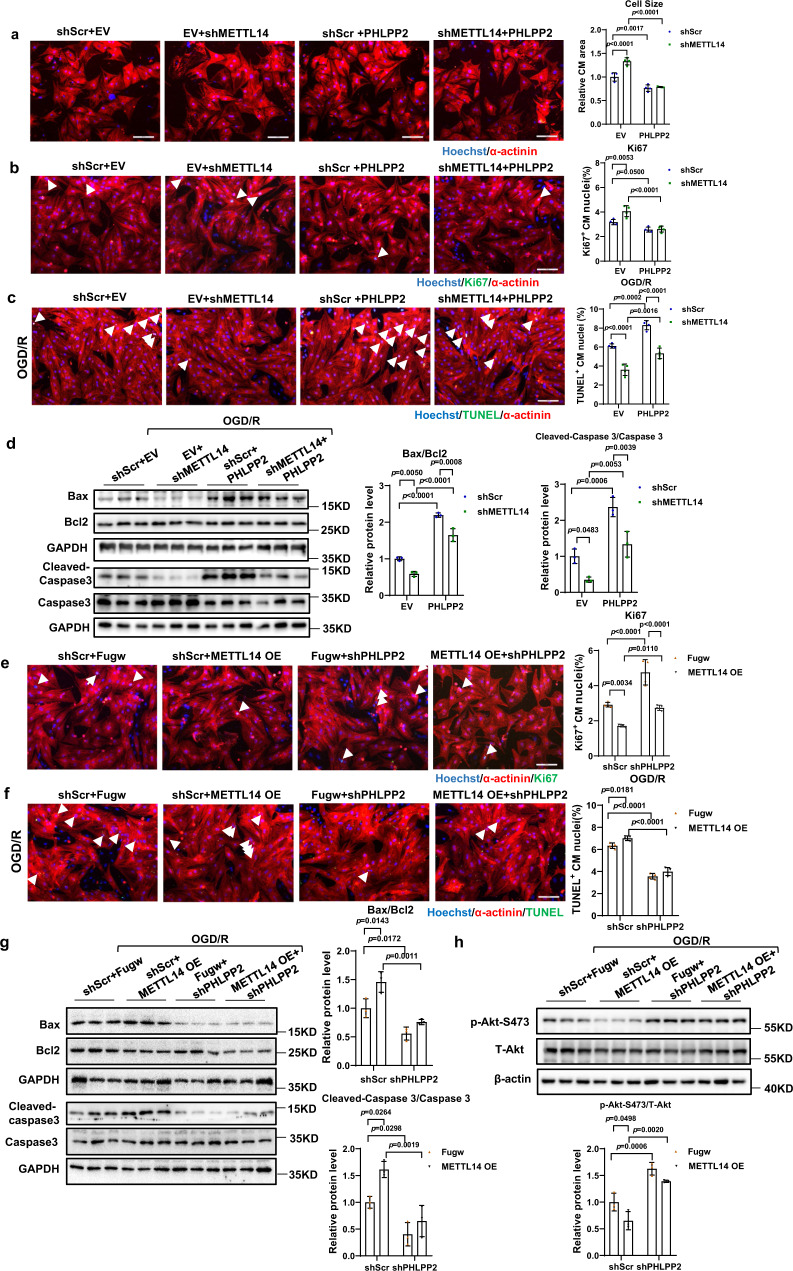
METTL14 regulates NRCMs growth and apoptosis via PHLPP2. **a** Representative images of immunofluorescence staining and quantification of the relative cardiomyocyte size displayed the block effect of PHLPP2 overexpression on METTL14 knockdown (*n* = 4 wells/group). Scale bar: 100 μm. **b** Representative images of immunofluorescence staining and quantification of the Ki67 positive cardiomyocytes displayed the block effect of PHLPP2 overexpression on METTL14 knockdown (*n* = 4 wells/group). Scale bar: 100 μm. **c** Representative images of immunofluorescence staining and quantification of the Tunel positive cardiomyocytes displayed the block effect of PHLPP2 overexpression on METTL14 knockdown in the OGD/R-induced apoptosis model (*n* = 4 wells/group). Scale bar: 100 μm. **d** Representative western blot and statistical data of NRCMs apoptosis by detection of Bax, Bcl2, Caspase 3, and Cleaved-Caspase 3 in OGD/R induced apoptosis model revealed the block effect of PHLPP2 overexpression on METTL14 knockdown (*n* = 3 wells/group). **e** Representative images of immunofluorescence staining and quantification of the Ki67 positive cardiomyocytes revealed the rescue effect of PHLPP2 knockdown on METTL14 overexpression (*n* = 4 wells/group). Scale bar: 100 μm. **f** Representative images of immunofluorescence staining and quantification of the Tunel positive cardiomyocytes demonstrated the rescue effect of PHLPP2 knockdown on METTL14 overexpression in the OGD/R-induced apoptosis model (*n* = 4 wells/group). Scale bar: 100 μm. **g** Representative western blot and statistical data of NRCMs apoptosis by detection of Bax, Bcl2, caspase 3, and Cleaved-caspase 3 in the OGD/R-induced apoptosis model demonstrated the rescue effect of PHLPP2 knockdown on METTL14 overexpression (*n* = 3 wells/group). **h** Representative western blot and statistical data of the phosphorylation levels of Akt-S473 in the OGD/R-induced NRCM apoptosis model displayed the rescue effect of PHLPP2 knockdown on METTL14 overexpression (*n* = 3 wells/group). Fugw, control without METTL14 overexpression; METTL14 OE, METTL14 overexpression; shScr, Scramble short hairpin RNA; shMETTL14, METTL14 short hairpin RNA to knockdown METTL14; EV, empty vector without PHLPP2 overexpression; PHLPP2, PHLPP2 overexpression; shPHLPP2, PHLPP2 short hairpin RNA to knockdown PHLPP2; OGD/R, oxygen-glucose deprivation/reperfusion; NRCM, neonatal rat cardiomyocyte. All data are expressed as means ± SD. **a**–**h** Two-way ANOVA followed by Tukey’s post hoc test. Source data are provided as a Source Data file.

Finally, we investigated if PHLPP2 mediated the biological function of METTL14 on cardiomyocyte growth and apoptosis, and performed a functional rescue assay in NRCMs. Analysis of western blots confirmed downregulation of PHLPP2 upon shPHLPP2 treatment and upregulation of PHLPP2 by OE PHLPP2 treatment (Supplementary Fig. 9). Positive regulation of PHLPP2 by METTL14 was also verified in OGD/R-treated NRCM and I/R -3w murine hearts (Supplementary Fig. 10). We subsequently tested whether forced overexpression of PHLPP2 could rescue the phenotype caused by METTL14 knockdown. As shown in Fig. 8a, b, PHLPP2 overexpression could rescue the pro-hypertrophy effects of METTL14 knockdown. In addition, the anti-apoptotic effects of silencing METTL14 were blocked by PHLPP2 overexpression as evaluated by TUNEL staining and western blot detection of apoptosis-associated genes (Bax/Bcl2, and cleaved-caspase 3/ caspase 3) (Fig. 8c, d). Furthermore, we demonstrated that PHLPP2 knockdown rescued reduced Ki67 positive nuclei in METTL14 overexpression treated NRCMs (Fig. 8e). Besides, TUNEL staining and western blot revealed that inhibition of PHLPP2 blunted the pro-apoptosis METTL14 overexpression in OGD/R-induced NRCMs (Fig. 8f, g). Meanwhile, the phosphorylation level of Akt-S473 regulated by METTL14 in OGD/R induced NRCM was blunted by PHLPP2 alteration (Fig. 8h). In summary, these results suggest that PHLPP2 acts as the downstream factor of METTL14 to regulate NRCMs growth and apoptosis.

## Discussion

**Fig. 9:**
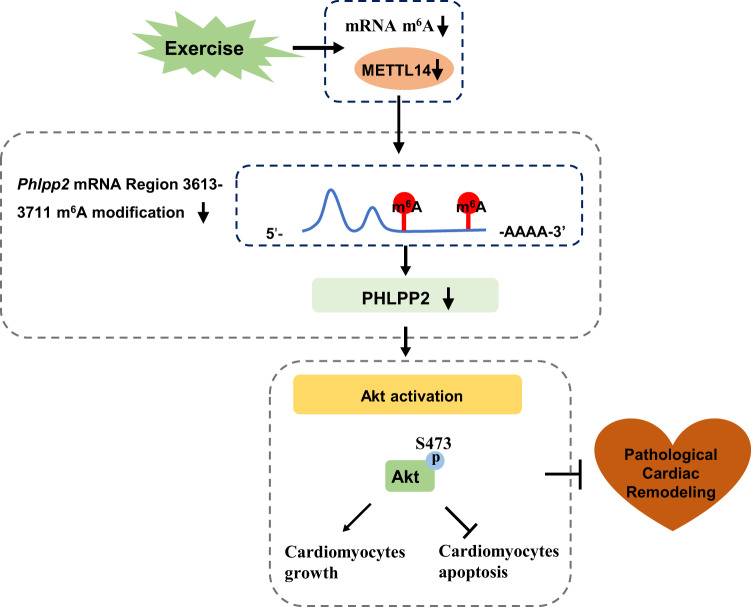
Proposed working model by which downregulation of RNA m^6^A methyltransferase METTL14 contributes to exercise-induced cardiac hypertrophy and protects against myocardial ischemia-reperfusion injury. Endurance exercise leads to a reduced mRNA m^6^A methylation level. Cardiac-specific knockdown METTL14 reduces global RNA m^6^A modification, thereby attenuating acute I/R injury as well as cardiac dysfunction in I/R remodeling. Mechanistically, silencing METTL14 regulates *Phlpp2* mRNA (ranging nucleotides 3613–3711 in *Rattus*) m^6^A modification and activates Akt-S473 which regulates cardiomyocyte growth and apoptosis.

Beneficial effects of regular exercise training on the whole-body are well recognized and exercise intervention has been proven to have significant effects on many diseases, such as obesity, diabetes, and cardiovascular diseases^2,34,35^. Many studies have reported inconsistent expression between mRNA levels and their corresponding protein levels, indicating the essential role of post-transcriptional regulation^10,12,36,37^. This indicates that focus on expression of the mRNA level alone and associated changes is not sufficient to explain the underlying mechanism for a given disease phenotype. RNA m^6^A is a highly conserved methylation modification that occurs across mammalian cells, involved in regulating RNA metabolism and various pathophysiological processes. In the heart, dysregulation of RNA m^6^A can lead to abnormal function of cardiomyocytes and imbalance of cardiac homeostasis^9,10,38^. The role of RNA m^6^A modification in regular exercise, as well as exercise-induced physiological cardiac hypertrophy, however, remains unknown. In this study, we demonstrated that endurance exercise decreased the cardiac mRNA m^6^A methylation level. One of the core components of the methyltransferase complex, METTL14, was found to be downregulated upon exercise training, both at the RNA m^6^A modification level and at the protein level, indicating that the change of METTL14 is post-transcriptionally regulated through RNA m^6^A in response to exercise training. Downregulation of METTL14 promoted cardiomyocyte growth and resisted OGD/R-induced cardiomyocyte apoptosis in vitro. In vivo, wild-type METTL14, but not METTL14 mutant overexpression, inhibited physiological cardiac hypertrophy induced by exercise training. Cardiac-specific knockdown of METTL14 subsequently reduced the global m^6^A modification levels, and attenuated acute I/R injury as well as cardiac dysfunction in I/R remodeling. Mechanistically, silencing METTL14 regulated PHLPP2 mRNA m^6^A modification and activated Akt-S473, thereby, regulating NRCM’s growth and apoptosis (Fig. 9). Our data indicate that METTL14 plays an important functional role in maintaining cardiac homeostasis. Downregulation of METTL14 may represent a promising therapeutic strategy for I/R injury and pathological cardiac remodeling.

RNA m^6^A methylation is the most prevalent and reversible RNA modification in mammals. In the heart, m^6^A demethylase FTO has been shown to be downregulated in heart failure patients and mouse hearts. Thus, upregulation of FTO could improve decline of cardiac function and fibrosis in post-ischemia mice by promoting angiogenesis^10,12^. Forced expression of ALKBH5 has been shown to improve cardiac function after myocardial infarction^39^. We found that expression of demethylase FTO and ALKBH5 were negatively regulated by METTL14 alteration in the heart and in cultured neonatal cardiomyocytes (Supplementary Fig. 11). Besides, mildly up-regulated expression of FTO and ALKBH5 has also been observed in response to exercise training (Supplementary Fig. 2c). Reduced mRNA RNA m^6^A levels were detected with METTL14 inhibition, which supports that higher demethylase activity might exist simultaneously with lower methyltransferase activity, both contributing to the total amount of methylated RNA m^6^A. This indicates that METTL14 and demethylase FTO/ ALKBH5 might work together as part of a synergistic mechanism. Further investigations are required to understand the exact underlying regulatory mechanism of this pathway. Of note, another partner, m^6^A methylase METTL3 controls cardiac homeostasis and hypertrophy as well. The cardiac-specific overexpression of METTL3 in mice has a protective effect on pathological myocardial hypertrophy, while cardiac-specific METTL3 knockout mice have been shown to exhibit abnormal cardiac function with aging and stress, indicating that a certain amount of RNA m^6^A is necessary to maintain cardiac homeostasis^9,40^. Very recently, the RNA m^6^A methylation activity of METTL3 was also reported to be controlled by piRNA CHAPIR^11^. METTL14 has been reported to participate in the regulation of many signaling pathways and progression of diseases^18–21^. Up to now, the function of METTL14 in the heart has remained largely unknown. In the present study, exercise training reduced the mRNA m^6^A modification level of METTL14. Regular exercise represents a typical stimulus that would not disrupt homeostatic regulation of physiological functions. Previous reports have suggested that METTL14 functions as a structural partner for m^6^A substrate recognition and activation of METTL3 via allostery^17^. In this present study, we found that a sustained and modest increase in the METTL14 protein level would dramatically blunt the effects of exercise training on cardiac physiological hypertrophy in vivo. Exercise-induced cardiac hypertrophy is a complex process that involves many factors and molecular pathways^2^. Here, we examined previously reported factors and signaling pathways known to be involved in exercise-induced cardiac hypertrophy, including IGF1/PI3K/Akt signaling, C/EBPβ, miR-222, *Cited4*, and lncRNA CPhar^4,5,7,41,42^. We found that METTL14 overexpression regulated the activation of Akt-S473 both in control and in mouse hearts that underwent swim-training. Interestingly, expression levels of above-mentioned exercise-induced cardiac hypertrophy associated factors were all regulated in response to swim-training, though METTL14 overexpression did not regulate them in the control group (Supplementary Fig. 12). These observations suggest that cardiac METTL14 overexpression led to deficiency of exercise-induced cardiac hypertrophy and according alteration of nearly all known associated factors. This observation might explain why a modest in vivo manipulation of METTL14 manipulation was sufficiently blunt effects of exercise. Though we cannot attribute the effect of exercise-induced cardiac hypertrophy to METTL14 alone, METTL14 manipulation is one of the major factors which appears essential for exercise-induced cardiac hypertrophy. It is very likely that other factors and signaling pathways are also involved in METTL14 regulation of exercise training-related cardiac hypertrophy. In this study, we found that cardiac METTL14 knockdown alleviated acute myocardial I/R injury, cardiac dysfunction, and I/R remodeling in mouse hearts. Furthermore, we propose that the observed downregulation of METTL14 is a moderate adaptive mechanism to maintain cardiac homeostasis upon exercise training. Of note, these data indicate that this could further benefit cardiac recovery upon I/R injury. Finally, our data provides evidence that exercise training impacts epigenetics through an RNA-related mechanism and gives us a better understanding of regulation of cardiac homeostasis and pathogenesis through m^6^A modification.

Despite importance of Akt signaling in the myocardial context, both in physiological and pathological processes, the role of m^6^A modifications in Akt signaling regulation has remained largely unknown^2,43^. Akt belongs to the AGC subfamily of protein kinases, which are composed of a PH domain, kinase domain, and regulatory domain (the hydrophobic motif). Residue Thr 308 is located in the active segment of the kinase domain, and additional phosphorylation of S473 in the hydrophobic motif of Akt stabilizes the active state, allowing most Akt to adopt an active formation^44^. Thus, phosphorylation of Thr 308 and Ser 473 are both required to maximal Akt activation^44–46^. In our present study, though we did not observe elevation of the Akt T308 phosphorylation level after METTL14 inhibition in cardiomyocytes, increased phosphorylation levels of specific Akt substrates, including FOXO3a, and GSK3β, were observed (Supplementary Fig. 13). This phenomenon might be due to increased phosphorylation on Akt S473, thereby increasing the negative charge of the hydrophobic motif, leading to an enhanced ability to allosterically stimulate Akt and activate the kinase activity. PHLPP2 regulates Akt-S473 phosphorylation as a phosphatase. Silencing of PHLPP2 leads to dephosphorylation of Akt-S473 and inhibition of Akt signaling^31,46^. As an essential regulator for Akt signaling, the roles of the PHLPP-family proteins have been well documented in several diseases, including cancers and metabolic disorders^47^. In addition to Akt1, we also detected phosphorylation of Akt2 and found that METTL14 overexpression or inhibition has no effect on phosphorylation of Akt2 in cardiomyocytes (Supplementary Fig. 14). This observation is also consistent with previously reported studies that Akt1 is the main isoform involved in regulating exercise-induced physiological hypertrophy^48^, while Akt2 takes important roles in glucose metabolism^49^. The PHLPP family contains two members, PHLPP1 and PHLPP2. PHLPP1 has been reported to inhibit leukemia inhibitory factor-induced Akt-S473 activation in the heart^50^. However, the *Phlpp1* mRNA m^6^A level and protein level were not affected by METTL14 as evidenced by luciferase assay, MazF-PCR, and western blot in this study (Supplementary Fig. 15a–c). Thus, PHLPP1 is unlikely to be the direct downstream factor of METTL14. However, we examined the expression of PHLPP1 in exercise training-induced healthy cardiac hypertrophy. Consistent with previous reports^51^, PHLPP1 was downregulated in swimming-trained hearts. Interestingly, like other previously reported factors and signaling pathways that have been known to be involved in exercise-induced cardiac hypertrophy, the PHLPP1 expression level was upregulated by METTL14 overexpression in response to swim training, though METTL14 overexpression did not regulate this in the control group (Supplementary Fig. 15d). Thus, it is possible that PHLPP1 might work together with PHLPP2 to inhibit Akt-S473 when METTL14 overexpression is present in the exercised heart. However, the underlying mechanism about how METTL14 regulate PHLPP1 in exercise hearts require further investigation. Downregulation of PHLPP2 could alleviate hypoxia-induced cardiomyocyte injury by enforcing Nrf2/ARE signaling^52^. In the present study, similar to previous reports in endometrial cancer and type 2 diabetes, we found that differentially regulated m^6^A-methylated genes were enriched in Akt signaling^18,20^. The m^6^A modification levels of PHLPP2 were positively regulated by METTL14. Though the IGV of *Phlpp2* was not significantly differentially modified in meRIP-seq, we observed the decreased m^6^A methylation level of *Phlpp2* mRNA using MazF-qPCR in the Control and Swim groups (Supplementary Fig. 16a). The observed inconsistency among different m^6^A methylation detection methods might be due to the resolution of meRIP-seq which in turn did not achieve nucleotide profiling. The m^6^A peak region (chr8:109939973 - 109941393) of *Phlpp2* we obtained from meRIP-seq data was as long as 1421 bp. Using the RMBase database^33^, we found over 20 potential m^6^A sites’ positions across this *Phlpp2* peak region, while only in the region between 3664–3734 the m^6^A methylation level decreased. *Phlpp2* was regulated through a region which was much shorter than the length of m^6^A peak region we acquired in IGV (Supplementary Fig. 16b). We further conducted SELECT-qPCR (An elongation- and ligation-based qPCR amplification method)^53,54^, and confirmed that m^6^A methylation level at mRNA A3675 and A3733 of *Phlpp2* mRNA were decreased, specifically A3675 revealed the most dramatic reduction (Supplementary Fig. 16c). The IGV from meRIP-seq represented the m^6^A methylation level of this region, while it cannot accurately reflect the m^6^A methylation level at such a large peak spanning many m^6^A peaks^55,56^. However, further investigations with application of RNA sequencing that elucidate m^6^A modification of *Phlpp2* mRNA at the level of the individual nucleotide would provide significant understanding of this observation and might explain where exactly this discrepancy derives from. Moreover, functional rescue assays confirmed that METTL14 regulated cardiomyocyte growth and apoptosis via PHLPP2. In contrast to our observation in exercised mouse hearts, a previous study showed that a reduced METTL14 level in mouse islet β-cells as well as in mouse embryonic stem cells results in decreased phosphorylation of Akt and subsequent cell cycle arrest^18,57^. However, restriction of METTL14 expression in hematopoietic stem cells and endometrial cancer cells has led to increased cell proliferation, which is consistent with our observation, suggesting that complex and distinct METTL14-mediated regulation mechanisms are present in different cell types^20,58^. Taken together, we report that METTL14 regulates Akt signaling by altering m^6^A modification of *Phlpp2* mRNA in cardiomyocytes, revealing a possible molecular mechanism underlying the role of RNA m^6^A modification in exercise-induced physiological cardiac hypertrophy.

Cardiovascular diseases remain the main cause for morbidity and mortality worldwide. Increasing evidence has shown that epigenetic regulation takes an essential role in cardiac pathophysiology^59,60^. Like other modifications, such as DNA methylation and histone modifications, the m^6^A modification in RNA is dynamic and reversible. Reversibility of m^6^A modification might provide an explanation for the fast regulatory mechanism of gene expression in response to certain stress stimulation, thereby providing also evidence of great significance for maintaining physiological homeostasis of the body, including homeostasis of the heart^9–13,40^. In this study, we focused on how RNA m^6^A methylation levels were influenced in response to endurance exercise on the post-transcriptional level. We reported that METTL14-mediated m^6^A modification was important to maintain exercise-induced physiologic cardiac hypertrophy. Besides, as in previous studies that reported changes of the heart’s global m^6^A modification, we found that it was important for cardiac homeostasis. Downregulation of cardiac METTL14 would prevent the heart to adequately respond to acute I/R injury and preserve cardiac function during I/R remodeling. Our findings support the essential role of RNA m^6^A modification and uncover the underlying mechanism of METTL14-mediated PHLPP2 posttranscriptional regulation in the heart.

In our current study, we identified the potential role of METTL14 in the heart from the perspective of RNA m^6^A methylation. We then focused on regulation of Akt signaling and specifically examined the m^6^A regulation of METTL14 on PHLPP2. However, RNA m^6^A modification alteration is a globally regulated mechanism, therefore, the change of METTL14 in the heart might methylate several transcripts not only limited to Akt signaling and *Phlpp2* transcript. Also, in addition to *Phlpp2*, other factors might also contribute to the regulatory effect of Akt-S473 on METTL14. Besides, among the effectors we detected, except RBM15B which did not exhibit a significant change, demethylase FTO and ALKBH5 were both increased. On the other hand, methyltransferases component METTL3, METTL14, WTAP, and ZC3H13 were decreased on the level of protein expression after swim-training as evidenced by western blot. Additional studies are required to explore their specific functional roles and mechanism of action. Currently, we disrupt the METTL3-METTL14 formed, positively charged groove, and abolish MTase activity *via* a METTL14 mutant, indicating that the observed effects might be METTL3–METTL14 complex dependent. However, as abolishing the METTL3-METTL14 pathway also inhibits global mRNA m^6^A levels, we cannot exclude the possibility that other m^6^A regulators besides METTL14, even readers, might also contribute to the cardiac adaption to exercise. Further investigations including meRIP-seq, specifically elucidating the global m^6^A methylation pattern change of specific m^6^A effectors or RIP-seq which can focus on a specific m^6^A binding proteins’ recognition pattern in cardiomyocytes, would provide a significant and deep understanding of the RNA m^6^A regulatory mechanisms in the exercised heart. The cardiac RNA m^6^A machinery is complex, in addition to the specific RNA m^6^A effectors, further investigations focusing on systematic study of the m^6^A levels of target genes of m^6^A regulators, and deeper understanding their biological significance as well as underlying regulatory network would offer significant opportunity to get insight into the coordination between RNA metabolism and exercise training in the heart. Further efforts will be needed to fully understand the role of RNA methylation in cardiac tissue.

In summary, we identify METTL14 as a key methyltransferase that is involved in regulating exercise-induced cardiac hypertrophy. METTL14 downregulation attenuates acute I/R injury and cardiac dysfunction during the I/R remodeling process. METTL14 downregulation in vitro regulates cardiomyocytes growth and OGD/R-induced apoptosis via PHLPP2. Mechanically, METTL14 knockdown inhibits the m^6^A level of *Phlpp2* mRNA and activates Akt-S473. Inhibiting METTL14 might be an effective therapeutic strategy for various heart diseases.

## Methods

### Animal care and use

Adult Male C57BL/6 J mice (8–9 weeks) were obtained from the Charles River Laboratories (Beijing, China) and housed in a barrier facility on a 12 h light/dark cycle at 22–24 °C and 45–55% humidity with access to food (Cat#1010083, XietongShengwu, Nanjing, China) and water ad libitum. The numbers of mice in each performed experiments were indicated in figure legend. All animal experiments were in accordance with the guidelines on the use and care of laboratory animals for biomedical research published by the National Institutes of Health (No. 85–23, revised 1996), and approved by the committee on the Ethics of Animal Experiments of Shanghai University (No. 2020-043).

For exercise swimming training, eight-week-old male C57BL/6 J mice were used for 4 weeks swimming training or no-training (control)^4,61^. Briefly, swimming training mice are starting from 10 min twice daily with a 10-minute increase each day until reaching to 90 min. 24 h after the last swimming session, mice hearts were collected for further analysis. The heart weight, body weight, and tibia length were measured for statistical analysis. To study the role of METTL14 in exercise-induced physiological cardiac hypertrophy, mice were treated with adeno-associated virus 9 (at the dose of 10^12^ vg/mice) carrying the cTnT promoter driven METTL14 overexpression, METTL14 mutant overexpression, and its control via tail vein injection one week before being subjected to swimming training. Each mouse was randomly assigned to each group. The body weights of mice before and after swim training/no training, and tibia length data, heart weights of the mice after swim training/no training in this study related to Figs. 3 and 4 have been listed in Supplementary Table 1.

For the cardiac ischemia-reperfusion (I/R) model, surgeries were performed under isoflurane anesthesia and using a ventilator to acquire passive respiration. Cardiac I/R was induced by ligation of the left anterior descending artery (LAD) for 30 min followed by cardiac reperfusion for 24 h (acute I/R injury) or 3 weeks (I/R remodeling)^62^. The sham group was treated by the same process except that the ligation part was not performed. For TTC staining, 24 h after reperfusion, the LAD was re-ligated at the same location, 1 mL Evan’s Blue (Sigma) was injected and the hearts were stained with TTC^62^. To assess surgery homogeneity, area at risk/left ventricle weight (AAR/LV) was calculated. The infarct size/area at risk (INF/AAR) was calculated to evaluate the severity of cardiac I/R. To study the role of METTL14 knockdown in I/R, a miR-30d-based shRNA framework was used to generate the cTnT promoter-driven METTL14 shRNA to restrict shMETTL14 expression to cardiomyocytes^30^. Adult C57BL/6 J mice were injected AAV9-cTnT-shMETTL14 or scrambled control through tail vein injection at the dose of 10^11^ vg/mice, and myocardial I/R or sham surgery was carried out 1 week (for acute I/R injury, TTC staining, and I/R remodeling) after AAV9 injection. All surgeries were performed by investigators blinded to the treatment. Mice were deeply anaesthetized and sacrificed for further analysis.

### Echocardiography

Mice were anesthetized with 2% isoflurane, and echocardiography using the Vevo 2100 image system (VisualSonics Inc, Toronto, Ontario, Canada) was performed. A center frequency of 30 MHz was used. M-mode echocardiograms were obtained at the level of the papillary muscle to measure left ventricular ejection fraction (EF) and left ventricular fractional shortening (FS). The echocardiographer was blinded to the genotypes and surgical procedure. Data from mice with heart rates lower than 400 beats/min were excluded from further analyses for the reason that cardiac function measured by echocardiography should maintain the heart rates at 400–650 beats/min to ensure physiological relevance^63^. All the echocardiography parameters measured in this study have been listed in Supplementary Table 2.

### m^6^A-seq and RNA-seq

To achieve a high enough RNA concentration, 2–3 mice left ventricular (LV) tissues were pooled and homogenized as one biological replicate. Total RNA was isolated from swim-trained and control mice LVs and treated with DNase I. Polyadenylated mRNA was further enriched from total RNA using Dynabeads Oligo (dT)_25_ (Ambion, Cat#61005) following the manufacturer’s protocol. mRNA was then fragmented into ~100 nucleotide fragments using fragmentation buffer (10 mM ZnCl_2_, 10 mM Tris-HCl pH7.0). Fragmented RNA was incubated for 2 h at 4 °C with affinity purified anti-m^6^A rabbit polyclonal antibody (Synaptic Systems, Cat#202003) in m^6^A Binding buffer (50 mM Tris-HCl, pH 7.4, 150 mM NaCl, 1% NP-40, 2 mM EDTA) supplemented with BSA (0.5 μg/μL). The mixture was then immunoprecipitated by incubation with protein A beads and eluted with elution buffer (1 × IP buffer and 6.7 mM m^6^A). After precipitation with 75% ethanol, eluted mRNA-containing fragments (IP) and untreated input control fragments were used to prepare libraries with a strand-specific library by the dUTP method. The libraries were sequenced on an Illumina Hiseq Novaseq 6000 platform and 150 bp paired-end reads were generated.

### m^6^A-seq data analyses

m^6^A-seq data was analyzed according to the protocol described by Yu et al.^64^. Briefly, the clean reads were mapped to the mouse genome (GRCm38) using HISAT2 (v2.1.0)^65^. Then MeTDiff (v1.1.0) was used to call m^6^A peaks and differential m^6^A peaks^24^ and peak annotation was performed using chipseeker (v1.12.1)^66^. The m^6^A peak calling for the meRIP-seq data was computed by the HEPeak based on a Hidden Markov Model-based Exome Peak-finding algorithm^67^. To obtain the differential m^6^A methylation peaks in the control and the swim-trained group, MeTDiff software was used to determine the putative differential m^6^A peaks and compute the statistical significance^24^. Briefly, the putative differential m^6^A peaks were determined as the union region of the corresponding methylation peaks in the swim-trained or control samples which are predicted by HEPeak peak calling software^24,67^. Then, MeTDiff models were used to determine the reads variation in a differential analysis also using a beta-binominal distribution. Finally, the statistical significance was determined using a likelihood ratio test. FPKM of each gene was calculated by Cufflinks (v2.2.1)^68^ using the sequencing reads from input samples. DESeq (v1.28.0) (2012) R package (v3.5.1) was used to find the differentially expressed genes (DEGs)^58^. KEGG pathway enrichment analysis of m^6^A peaks, differential m^6^A peaks, and DEGs were performed using R based approach, based on the hypergeometric distribution, respectively.

### m^6^A RNA immunoprecipitation(meRIP)-qPCR

MeRIP-qPCR was performed according to a previously reported method^69^. Briefly, Total RNA was extracted from H9C2 cardiomyocytes by miRNeasy Mini Kit (QIAGEN, Cat# 217004) with DNase I on column treatment (QIAGEN, Cat# 79254). mRNA was isolated from total RNA via oligo(dT) polystyrene beads (Sigma, Cat#MRN10) and fragmented (Ambion, AM8740) at 70 °C for 15 min. 1 μg of fragmented mRNA was saved as input sample. The remaining fragmented mRNA was immunoprecipitated with an anti-m^6^A antibody (Synaptic Systems, Cat#202003) coupled Dynabeads Protein G (Invitrogen, Cat#10004D). PCR primers of specific genes were designed based on the meRIP-seq data and RMBase database which integrated the public epitranscriptome sequencing data and annotated the RNA m^6^A methylation sites of genes^33^. m^6^A enrichment was determined by qPCR with specific gene primers listed in Supplementary Table 3.

### MazF-qPCR

MazF-qPCRs were performed as reported^25^. Specifically, total RNAs were extracted from H9C2 cardiomyocytes or mouse hearts by miRNeasy Mini Kit (QIAGEN, Cat# 217004) with DNase I on column treatment (QIAGEN, Cat# 79254). mRNA was isolated from total RNA via oligo(dT) polystyrene beads (Sigma, Cat#MRN10-1KT). mRNAs or total RNAs were treated with MazF (Takara, Cat# 2415 A) according to the manufacturer’s protocol. PCR primers of specific genes were designed based on the meRIP-seq data and RMBase database data^33^. For Fig. 7k and Supplementary Fig. 16b, meRIP-seq data, sequence alignment, and RMBase database were based to design the MazF-qPCR primers as indicated in Supplementary Table 4^33,70^. Primer sequences used in this study are listed in Supplementary Table 3.

### SELECT-qPCR detection

Total RNA was extracted from the mice hearts using RNAiso Plus (Takara, Cat# 9109), and mixed with 40 nM Up Primer, 40 nM Down Primer, and 5 μM dNTPs in 17 μl 1×NEBuffer4 (NEB, #B7004S). Total RNA (1500 ng) and the primers were incubated at a temperature gradient (90 °C for 1 min, 80 °C for 1 min, 70 °C for 1 min, 60 °C for 1 min, 50 °C for 1 min, 40 °C for 6 min)^53,71^. Then, 0.1 U Bst 2.0 WarmStart DNA polymerase (NEB, #M0538S), 5U SplintR ligase (NEB, #M0375S), 50μΜ dTTP (Diamond, #B110051-0250) and 1 mM ATP (NEB, #P0756S) was added in the mixture from the previous step to the final volume 100 μL. The mixture was incubated at 40 °C for 20 min, followed by 80 °C for 20 min, and kept at 4 °C. The final elongated and ligated products are then quantified by qPCR. Sequences used in this study are listed in Supplementary Table 5.

### Primary neonatal rat cardiomyocytes (NRCM) preparation and culture

Primary neonatal rat cardiomyocytes (NRCMs) were isolated from euthanized 1-3-day old Sprague-Dawley (SD) rats (male and female) and purified by Percoll (GE health, Cat# 17-0891-09) gradient centrifugation^62^. Isolated NRCMs were seeded in appropriate plates and cultured in Dulbecco’s modified Eagle’s medium (DMEM) (CORNING, Cat#10-013-CVR), supplemented with 5% fetal bovine serum, and 10% horse serum at 37 °C in 5% CO_2_.

### In vitro oxygen–glucose deprivation and reperfusion (OGD/R) model

The OGD/R model was established to mimic in vivo I/R injury^62^. Briefly, DMEM with no glucose was used and cultured NRCMs in an Anaero pack (MGC, Cat# 6361ZJ-3) to achieve oxygen-glucose deprivation. After 8 h, NRCMs were transferred to a normal incubator and cultured in DMEM with glucose for recovery 12 h.

### Cell line

Rat H9C2 cardiomyocytes were kindly provided by Stem Cell Bank, Chinese Academy of Sciences. HEK293T cells was kindly gifted by Dr. Qiurong Ding at Chinese Academy of Sciences, Shanghai, China, originally purchased from Stem Cell Bank, Chinese Academy of Sciences. H9C2 cardiomyocytes and HEK293T were maintained in Dulbecco’s modified Eagle’s medium (DMEM) supplemented with 10% Fetal Bovine Serum (BI) and at 37 °C in a 5% CO_2_ cell culture incubator.

### Bacterial strains

*E.coli* expression strain TransStbl3 Chemically Competent Cell were purchased from TransGen Biotech were grown in LB culture at 37 °C.

### Quantification of m^6^A in mRNA

Total RNAs were extracted from murine hearts by miRNeasy Mini Kit (QIAGEN, Cat# 217004) with DNase I on column treatment (QIAGEN, Cat# 79254). mRNA was isolated from total RNA via oligo(dT) polystyrene beads (Sigma, Cat#MRN10). To achieve sufficient enough mRNA concentration and make sure the quantity of purified mRNA, 2–3 mice left ventricular tissues or about (2–4) × 10^6^ NRCM were pooled and homogenized as one biological replicate. For mRNAs m^6^A relative quantification to determine the relative m^6^A RNA methylation status of two or three different RNA samples, m^6^A RNA Methylation Quantification Kit (Abcam, #ab185912) were used according to the manufacturer’s protocols. Briefly, 200 ng of mRNA was added to a 96-well plate. After RNA binding, m^6^A RNA capture, signal detection, relative quantification of m^6^A was analyzed as recommended by the manufacturer.

### Quantitative real-time polymerase chain reactions(qRT-PCRs)

Total RNA was isolated using RNAiso Plus (Takara, Cat# 9109). RNA concentration was measured by Nanodrop 2000 (Thermo fisher, USA), and reverse transcription of 400 ng RNA was performed using a reverse transcription PCR kit (Thermo scientific, Cat#K1622) according to the manufacturer’s instructions. RNA levels were quantified using TB Green Premix Ex Taq (Takara, Cat#RR001A) by Real-Time PCR Detection System (Roche LightCycler480). 18 s RNA or GAPDH were used as internal controls. Primer sequences used in this study are listed in Supplementary Table 3.

### Cell transfection and virus infection

For transfection overexpression and knockdown plasmids into NRCMs, plasmids (1 μg/mL) were carried out with Lipofectamine 2000 Reagent (Invitrogen, Cat#11668-019) or Sino transfection reagent (Sino Biological Inc., Cat#STF02) according to the manufacturer’s protocols. For lentiviral particles transfection to cardiomyocytes, lentiviral shMETTL14 was performed at the dose of 10^8^ transduction unit (TU) per mL medium. At the end of the experiments, the efficiency of transfection was evaluated by qPCR or western blot. shRNA sequences used in this study are listed in Supplementary Table 6 and primer sequences used for generation of overexpression constructs used in this study are listed in Supplementary Table 7.

### Western blotting

The NRCMs or cardiac tissues were lysed using RIPA lysis buffer (KenGEN, Cat#KGP-701-100). The protein sample (10 μg) was loaded onto sodium dodecylsulphate polyacrylamide gel electrophoresis. Membranes were electro-blotted onto PVDF membrane (PALL, Cat#BSP0161), and then incubated with the appropriate primary antibodies at 4 °C overnight. Mouse or rabbit IgG antibodies coupled to horseradish peroxidase were used as secondary antibodies. All proteins were visualized by hypersensitive chemiluminescence kit using ChemiDoc Imaging System (Tanon, 5200 S or Bio-Rad, 17001402) and band gray statistics were analyzed using Image J with β-actin and GAPDH as the loading control. Antibodies used in this study are listed in Supplementary Table 8.

### Luciferase reporter assays

For PHLPP2 reporter gene generation, the PHLPP2 mRNA fragment containing the wild-type (WT) or mutant m^6^A motifs (A to T), were inserted into pGL3-Basic(gccgtg/taattc) vector. The sequences of PHLPP2 reporter genes used in this study are listed in Supplementary Table 9. HEK293T cells were cultured in 12-well plates the day prior to transfection. For PHLPP2 mutant assays, cells were co-transfected with 40 ng Renila, 1 μg wild-type or mutant PHLPP2 reporter vector and METTL14 OE. For METTL14 mutant assays, cells were co-transfected with 40 ng Renila, 1 μg wild-type PHLPP2 reporter vector and METTL14 OE or METTL14 mutant (R234/235A-D292A-R278P^METTL14^). Firefly and Renila luciferase activities were detected using the Dual-luciferase reporter assay Kit (Promega, Cat# E1910) according to the manufacturer’s instructions.

### Immunofluorescent staining for cultured cells

Cardiomyocytes were fixed in 4% PFA for 20 min at room temperature, permeabilized with 0.2% Triton X-100 for 20 min, blocked with 10% goat serum for 1 h at room temperature and incubated with primary antibodies at 4 °C overnight. Cardiomyocytes were incubated with α-actinin antibody (1:200, Sigma, A7811), Ki67 antibody (1:200, Abcam, ab16667), and pHH3(1:200, Thermo Fisher Scientific, PA5-17869). For EdU assays, NRCMs were performed using Cell-Light EdU Apollo488 In Vitro Kit according to the manufacturer’s instructions. The following secondary antibodies were used: Cy3-mouse antibody (1: 200, Jackson, 715-165-151), 488-rabbit antibody (1: 200, Jackson, 111-545-003). Hoechst was used for nuclear counterstaining. Images were captured by Leica microscope (DMi8, Germany). Both EdU-/ Ki67-/pHH3- positive and α-actinin positive nuclei was counted for cardiomyocyte EdU-/ Ki67-/pHH3- positive.

For cell size measurements, NRCMs were incubated with α-actinin, and Hoechst was stained for counterstaining cell nuclei. Images were captured by Leica microscope (DMi8, Germany). Image J software was used to measure cell size. 40–60 fields were analyzed on each group, and 400–600 cardiomyocytes measured in each group.

For terminal deoxynucleotidyl transferase-mediated dUTP in situ nick-end labeling (TUNEL) staining, TUNEL FITC apoptosis detection kit (Vazyme) was stained to evaluate the cardiomyocytes apoptosis according to according to the manufacturer’s protocol after α-Actinin-Cy3 antibody (1: 200, Sigma, A7811). Nuclei were counterstained with Hoechst. Images were captured by Leica microscope (DMi8, Germany). TUNEL positive and α-actinin positive nuclei was counted for cardiomyocyte TUNEL positive.

### Immunochemistry and immunofluorescence staining for heart tissue

5 μm frozen heart sections were fixed in 4% paraformaldehyde (PFA) for 20 min, permeabilized with 0.5% Triton X-100 in PBS for 20 min, and then blocked with 5% BSA for 1 h at room temperature. For EdU staining, EdU (50 mg/kg) (Invitrogen, Cat#E10187) was intraperitoneally injected twice at three days before and the day before termination of the experiment at 50 mg/kg, respectively. Cell-Light Apollo 567 stain Kit (Ribobio, Cat#C10371-1) was used according to manufacturer’s instructions. For Ki67 staining, an α-actinin antibody (1:200, Sigma) and Ki67 antibody (1:200, Abcam) were co-incubated, and following appropriate secondary antibodies were used. Nuclei were counterstained with Hoechst. Images were captured by confocal microscope (Carl Zeiss, Thuringia, Germany).

For WGA staining, 5 μm frozen heart sections were fixed in 4% paraformaldehyde (PFA), wheat germ agglutinin Alexa Fluor™ 488 Conjugate (Invitrogen, Cat# W11261) was incubated for 20 min. Images were taken by confocal microscope (Carl Zeiss, Thuringia, Germany). Image J software was used to quantify cell size.

For TUNEL staining, Dead End Fluorometric TUNEL System (Promega, Cat#G3250) were used to perform TUNEL staining on 5 μm frozen heart sections according to the manufacturer’s protocol after α-Actinin-Cy3 antibody. Images were captured by confocal microscope (Carl Zeiss, Thuringia, Germany). TUNEL positive and α-actinin positive nuclei was counted for cardiomyocyte TUNEL positive.

Heart tissues were fixed in 4% PFA and embedded in paraffin, Paraffin sections were dewaxed and hydrated. Then, sections were subjected to Masson’s Trichrome staining (Servicebio, Cat# G1006) and H&E staining (KenGEN, Cat# KGA224) according to the manufacturer’s instruction, respectively. Images were taken by Leica microscope (DM3000 LED, Germany). Image J software was used to quantify the fibrotic area. The percentage of fibrosis was calculated as fibrosis areas/ total myocardial areas. Image J software was used to quantify cell size.

### Microscopy, confocal microscopy, and image quantification

For in vitro assays, images of cultured cells were taken by Leica microscope (DMi8, Germany) using standard procedures. 4–6 wells of each group were stained and in total 40–60 fields were analyzed on each group. For cell size measurement, 4–6 wells of each group were stained, and 40–60 fields were analyzed on each group, and 400–600 cardiomyocytes measured in each group. *n* value represents the number of wells as indicated groups.

For in vivo assays, immunofluorescence Images of heart sections were taken using a confocal microscope (Carl Zeiss, Thuringia, Germany). Masson’s Trichrome and H&E images were obtained using a Leica microscope (DM3000 LED, Germany). Images were then quantified using Image J software. 10–30 random images were obtained from 1–3 slides from each mouse. For WGA staining, at least 10 cells were quantified on each image: 100–300 cells from each mouse. *n* value represents the number of mice as indicated groups. All analyses were performed by investigators blinded to the treatment.

### Statistical analysis

All data are presented as mean ± SD using GraphPad Prism 8.0. Analyses were performed using SPSS 20.0 software (SPSS Inc., Chicago, IL) and GraphPad Prism 8.0. An independent-sample *t* test was used for comparison between two groups. Two-way ANOVA with Tukey test or One-way ANOVA was performed to compare multiple groups. For one-way analysis, The Levene test was used to verify the homogeneity of variance, and the Bonferroni test or Dunnett T3 test was performed according to the results. Differences were considered statistically significant with *p* < 0.05.

### Reporting summary

Further information on research design is available in the Nature Research Reporting Summary linked to this article.

## Supplementary information

Supplementary Information

Reporting Summary

## Data Availability

The data that support the findings of this study are available within the article and its Supplementary Information files. The MeRIP-seq and its input RNA-seq data that support the findings of this study have been deposited in the BioSample database under BioProject ID PRJNA699979. Sequencing reads were mapped to the mouse genome reference GRCm38. A reporting summary for this article is available as a Supplementary Information file. Source data are provided with this paper.

## Source data

Source Data
