# Supplementary material for: METTL14 is required for exercise-induced cardiac hypertrophy and protects against myocardial ischemia-reperfusion injury
Source: Nat Commun. 2022 Nov 9;13:6762. doi: 10.1038/s41467-022-34434-y (PMC9646739; doi:10.1038/s41467-022-34434-y)
Supplement: Supplementary file 1 — Supplementary Information [file 41467_2022_34434_MOESM1_ESM.pdf]

Supplementary Information For

METTL14 is required for exercise-induced cardiac hypertrophy and protects against myocardial ischemia-reperfusion injury

Lijun Wang, Jiaqi Wang, Pujiao Yu, Jingyi Feng, Guie Xu, Xuan Zhao, Tianhui Wang, H. Immo Lehmann, Guoping Li, Joost PG Sluijter, Junjie Xiao*

* correspondence: junjiexiao@shu.edu.cn (Junjie Xiao)

Supplementary Tables:

Supplementary Table 1. Related to method section.

The body weights of mice before and after swim training/no training, and tibia length data, heart weights of the mice after swim training/no training in this study related to Figure 3.

	Control group		Swim group	
	AAV9-cTnT-Control	AAV9-cTnT-METTL14	AAV9-cTnT-Control	AAV9-cTnT-METTL14
Before swim				
Body Weight(g)	24.60±0.756	24.08±0.914	24.33±1.007	24.21±0.614
After swim/no training				
Body Weight(g)	25.88±1.004	25.50±0.787	22.51±0.821	22.38±0.716
Heart Weight(g)	0.098±0.016	0.097±0.002	0.124±0.006	0.107±0.006
Tibia length(cm)	1.632±0.017	1.639±0.013	1.640±0.022	1.643±0.011
HW/TL(g/cm)	0.060±0.010	0.059±0.001	0.076±0.003	0.065±0.004
HW/BW(g/g)	0.004±0.0005	0.004±0.0001	0.006±0.0004	0.005±0.0003

The body weights of mice before and after swim training/no training, and tibia length data, heart weights of the mice after swim training/no training in this study related to Figure 4.

	Control group			Swim group		
	AAV9-cTnT-Control	AAV9-cTnT-$WT^{METTL14}$	AAV9-cTnT-$Mut^{METTL14}$	AAV9-cTnT-Control	AAV9-cTnT-$WT^{METTL14}$	AAV9-cTnT-$Mut^{METTL14}$
Before swim						
Body Weight(g)	25.68±1.072	25.76±0.979	25.65±0.391	25.61±0.899	25.68±0.757	25.69±0.441
After swim/no training						
Body Weight(g)	26.74±0.788	26.68±0.699	26.46±0.934	22.53±1.406	22.66±0.615	22.43±0.692
Heart Weight(g)	0.098±0.004	0.099±0.003	0.096±0.007	0.124±0.011	0.101±0.007	0.119±0.007
Tibia length(cm)	1.774±0.004	1.774±0.003	1.774±0.004	1.775±0.004	1.774±0.003	1.775±0.002
HW/TL(g/cm)	0.055±0.002	0.056±0.002	0.054±0.004	0.070±0.006	0.057±0.004	0.067±0.004
HW/BW(g/g)	0.004±0.0002	0.004±0.0002	0.004±0.0003	0.006±0.0003	0.005±0.0003	0.005±0.0003

Supplementary Table 2. Related to method section.

Parameters of echocardiography.

	Sham		I/R 3w	
	AAV9-cTnT-shScr	AAV9-cTnT-shMETTL14	AAV9-cTnT-shScr	AAV9-cTnT-shMETTL14
Heart rate (BPM)	479±48	458±34	450±29	438±17
LVESD (mm)	2.7±0.3	2.6±0.3	3.2±0.3**	2.4±0.4##
LVEDD (mm)	3.8±0.3	3.6±0.3	3.8±0.4	3.5±0.5
LV Volume; s (μl)	27.0±8.1	24.5±5.6	39.3±6.9*	20.9±10.4##
LV Volume; d (μl)	61.9±13.9	55.8±10.3	61.1±13.3	51.6±16.9
LVAW; s (mm)	1.1±0.1	1.2±0.1	1.0±0.1	1.2±0.1##
LVAW; d (mm)	0.8±0.1	0.8±0.1	0.9±0.1	0.9±0.1
LVPW; s (mm)	1.1±0.1	1.1±0.1	0.9±0.1	1.1±0.2#
LVPW; d (mm)	0.8±0.1	0.8±0.1	0.7±0.1	0.8±0.2
LV Mass (mg)	103.8±20.4	98.1±7.9	105.6±22.1	98.7±21.2
LV Mass Cor (mg)	83.0±16.3	78.5±6.3	84.5±17.7	78.9±17.0
Stroke Volume (μl)	35.0±6.5	31.3±6.0	23.5±4.8**	30.7±7.9
Cardiac output (ml/min)	16.6±2.5	14.3±2.9	10.3±2.3**	13.4±3.3

Abbreviation: BPM, beat per minute; LVESD, left ventricular end-systolic dimension; LVEDD, left ventricular end-diastolic dimension; LV Volume s, left ventricular volume systolic; LV Volume d, left ventricular volume diastolic; LVAWs, left ventricular anterior wall thickness systolic; LVAWd, left ventricular anterior wall thickness diastolic; LVPWs, left ventricular posterior wall thickness systolic; LVPWd, left ventricular posterior wall thickness diastolic; LV Mass, left ventricular mass index.

Data are presented as mean ± SD. *, $p<0.05$ compared to AAV9-cTnT-shScr +Sham; **, $p<0.01$ compared to AAV9-cTnT-shScr +Sham; #, $p<0.05$ compared to AAV9-cTnT-shScr +I/R 3w; ##, $p<0.01$ compared to AAV9-cTnT-shScr +I/R 3w. (Two-way ANOVA, Tukey)

Supplementary Table 3. Related to method section.

The primer sequences used for quantitative PCR were as follows:

	Forward	Reverse
For gene expression qPCR primers		
rat-Mettl14	TGGGGATCAAAGGAACCGTG	GCCTGATAGTGCTGTCCCTC
rat-Phlpp2	GCGTCTGACGAACACAACAC	CAGACTACAGCGCCTCTCAG
mmu-Mettl14	CTGAGAGTGCGGATAGCATTG	GAGCAGATGTATCATAGGAAGCC
mmu-Anp	TCTTCCTCGTCTTGGCCTTT	CCAGGTGGTCTAGCAGGTT
mmu-Bnp	TGGGAGGTCACTCCTATCCT	GGCCATTTCCTCCGACTTT
mmu-βMhc	CGGACCTTGGAAGACCAGAT	GACAGCTCCCCATTCTCTGT
mmu-αSma	GTCCCAGACATCAGGGAGTAA	TCGGATACTTCAGCGTCAGGA
rat-Anp	GAAGATGCCGGTAGAAGATGAG	AGAGCCCTCAGTTTGCTTTTC
rat-Bnp	GGTGCTGCCCCAGATGATT	CTGGAGACTGGCTAGGACTTC
rat-βMhc	GCCCCAAATGCAGCCAT	CGCTCAGTCATGGCGGAT
18s	TCAAGAACGAAAGTCGGAGG	GGACATCTAAGGGCATCAC
rat-Gapdh	TGACAACTCCCTCAAGATTGTCA	GGCATGGACTGTGGTCATGA
mmu-Gapdh	AGGTCGGTGTGAACGGATTTG	TGTAGACCATGTAGTTGAGGTCA
mmu-Mettl14-specific for Mutant	AAAGTGGGGTTACGCAGCA	CTAGGCTTCACGGTTCCTTTGA
mmu-Phlpp2	GCTCCCGAAGAACTCTTCCAA	GTCTTCGGATCCCAAACCCC
mmu-Mettl3	CTGGGCACTTGGATTTAAGGAA	TGAGAGGTGGTGTAGCAACTT
mmu-Wtap	GAACCTCTTCCTAAAAAGGTCCG	TTAACTCATCCCGTGCCATAAC
mmu-Fto	TTCATGCTGGATGACCTCAATG	GCCAACTGACAGCGTTCTAAG
mmu-Alkbh5	CGCGGTCATCAACGACTACC	ATGGGCTTGAACTGGAACTTG
mmu-Zc3h13	AGCACTGGAAGCACAACAGA	GTAGCCTTTCCCACGAGGTG
mmu-Rbm15b	TATAGCCGCTCAGTTCGCAG	GAGTGAGTTGTCCTCCCACG

For MazF-qPCR primers		
rat-Phlpp2-1	TCACATGGATTTGCGGGACA	TCTCAACTGGTTCCGCTCAC
rat-Phlpp2-2	GCGTCTGACGAACACAACAC	CAGACTACAGCGCCTCTCAG
rat-Phlpp2-3	GACATTCATTGCTGCCGAGG	CCCAACCCCCTAGCATGTTC
rat-Phlpp2-4	CCACAGAAGCACCCAAGAGG	GGTCACGGGGGACAACAAAT
rat-Phlpp2-5	CACTGTGGGAACACTTGTCCT	AGCAGCAGCTAATGGGTCTT
mmu-Phlpp2-0	ATGTGCCAAGCTGTCCTGTG	GGGTCATGCTCCAAGCTGAA
mmu-Phlpp2-1	GCTCCCGAAGAACTCTTCCAA	GTCTTCGGATCCCAAACCCC
mmu-Mettl14-0	TGCATCGTATTTCAGTGCCC	CGGTCGAAGCCTCTCGATTT
mmu-Mettl14-1	ACTCAAAACGGAAGTGTCTGG	TCTCTTCCTCCTGCTGCATTTC
mmu-Phlpp1-0	CCACCTAGGAGCCTTTCCGT	GCAGGGCATTGCAAGATACA
mmu-Phlpp1-1	CACCTGCCAGACTGTTACGA	TCTGGGAGAGGGCATGAAGT

mmu-Phlpp2-2	GTTGAGCACTAGCAGGGTCT	GGAGTCCAACCAAGGTCCAAT
mmu-Phlpp2-3	GCCAGCGAACAGAGACAAGA	CCTCTAGGGGCACAATGGAG
mmu-Phlpp2-4	TACCTCTCGTGTGGACGCAG	ATGGTTTGAAACACTGAGGCAA

Supplementary Table 4. Related to method section.

> *Rattus* PHLPP2(Reference Sequence: NM_001109131.2)

The potential m^6A modification sites were browsed by RMBase 2.0 database (Species *Rattus*).

Control Amplicon primer F1/R1

TCACATGGATTTGCGGACAATCAGTTGACTGATTTGGACCTTAGCTCCT
TATGTAGCTTGGAGCAGCTGCATTGTGAGCGGAACCAGTTGAGA

Targeting Amplicon primer F2/R2

GCGTCTGACGA**A**CACAACACTGTGGGCCTGGAGACGAGCTTGCTTCCCA
GGCCTGAGAGGCGCTGTAGTCTG

Targeting Amplicon primer F3/R3

Targeting Amplicon primer F4/R4

TCTGACAATGG**A**CTGG**A**CAGTGACGATGACCAGCCTGTTGAAGGCGTCA
TAACCAATGGCAGCAGGGTTGAGGTAGAGGTAGACATTCATTGCTGCCG
AGGCAGAGAGTCTGAG**A**ACTCCCCTACTCTCCCGAAGAATTCTTCCTAC
CCCTGTTCAGAGGA**A**CATGCTAGGGGGTTGGGTTTTGGGATCCGAAG**A**C
AGA**A**CAGTGTGAATAGTGGTATACTTCTGCCAGTGA**A**CAAAG**A**CAAGAT
GGAGTTACAGAAGTCTCCCTCCACTTCTTGTTTGTATGGAAAGAA**A**CTCT
CCAATGGCTCCATTGTGCCCCTAGAAG**A**CAGCCTGA**A**CCTCATTGAGGTG
GCCACAGAAGCACCCAAGAGGAAAACTGGCTATTTGCTGCCCCCACTC
AGCTGGAACCAGAGGATCAATTTGTTGTCCCCCGTGACCTGGAAGACGA
AGTGAAGGAGCAAATGAAGCAGCACCAGGAGAGCAGGCCTGAGCCTGA
ACCCAGGGAAGAGG**A**CCGG**A**CAGAGCCCCTGCAGGAGTTTGATACAGC
ACTGTGA

Targeting Amplicon primer F5/R5

CACTGTGGGA**A**CACTTGTCCTATCTAGAAGCTGTCAGTGCAGTCCGTCAT
GTGCAAG**A**CCCATTAGCTGCTGCT

*mmu*PHLPP2 m^{6}A peak region in meRIP-seq:

>Chr8:109939973-109941393(+)

TGTGCACATTAGCCCAGAGCTACGGTTGTCAGGACAACGTGGGGGCAAT
GGTGGTTTATTTGAATATTGGTGAAGAAGGCTGTACCTGTGAAATGAATG
GGCTCACCCTTCCAGGCCCTGTGGGATTTGCTTCAACTGCAGCCCTCAA
GGATACTCCCAAGCCAACCACTCCCTCCTCCAGTAGTGGGATTGCCTCCG
AGTTCAGCAGTGAGATGTCCACCTCAGAGGTGAGCAGTGAAGTGGGCTC
CACGGCCTCGGATGAGCACAACACTGTGGGCCTGGAGGCGAGCTTGCTG
CCCAGGCCAGAGAGGCGCTGCAGTCTGCACCCAGCGTCCTCCGCAGGG
GTGTTCCAGCGCCAGCCTTCTTGTGCTACTTTTTCCAGCAATCAGTCTGA
CAATGGCCTGGACAGTGACGATGACCAGCCTGTTGAAGGAGTCATAACC
AACGGCAGCAGGGTTGAAGTAGAGGTAGACATCCATTGCTGCCGAGGCA
GAGAGCCAGAGAGCTCCCCTCCGCTCCCGAAGAACTCTTCCAACGCCTG
TTCAGAGGAGCGTGCTAGAGGGGCGGGGTTTGGGATCCGAAGACAGAA
CAGTGTCAACAGTGGTATACTTCTGCCAGCGAACAGAGACAAGATGGAG
CTGCAGAAGTCTCCCTCCACTTCTTGTCTGTATGGAAAGAAACTCTCCAA
TGGCTCCATTGTGCCCCTAGAGGACAGCCTGAACCTCATTGAGGTGGCC
ACAGAAGCACCCAAAAGGAAAACTGGCTACTTTGCTGCCCCCACTCAGC
TGGAACCAGAGGATCAGTTTGTCGTCCCTCGAGACCTGGAAGAGGAAGT
GAAGGAGCAGATGAAGCAGCACCAGGAGGGCAGGCCTGAGCCCGAGCC
CCGGGGAGAGGAGCGGACAGAGCCCCTGGAGGAGTTTGATACAGCACT
GTGATTGTACCTCTCGTGTGGACGCAGCCTGAGGGAGGGCTGTGTGGTA
TTGGGGAAGGGACCTTCAGGAGATATTTGCCTCAGTGTTTCAAACCATAG
ATAATATGGAGTAGAATTTGGAGCCAAAAAGTTGAGCACTAGCAGGGTC
TGGCTCTTGATGGGCTCGGACCGCCATCAGTGTTAAGCTTCACATTGGAC
CTTGGTTGGACTCCCAGCGTTGCTGGGAGACAGCAGATGTTCTGTGTCA
GTCCTGCCACCTGCCATTAACCCTTTCTCTCCTAGGATCATTTGAGAAATT
GCCTGCCTGGGCAGGAAAGGGACTATTTCTGTGGAGGAAGTAACTGAAG
GTTGATTCCCTTTACTAATTGCTGCTGATGGATCTCTGTGACAAGAGAAAT

CACCTTATCTCTCAGACTAACTAGTGGGATGTGATGTGACTAGTCACATG GCTTTTTATTCTCTAGGAGAATACAGCCTATCAAATGTCTA
*mmu*PHLPP2 m^{6}A peak region and *Rattus* PHLPP2 (Reference Sequence: NM_001109131.2) alignment:

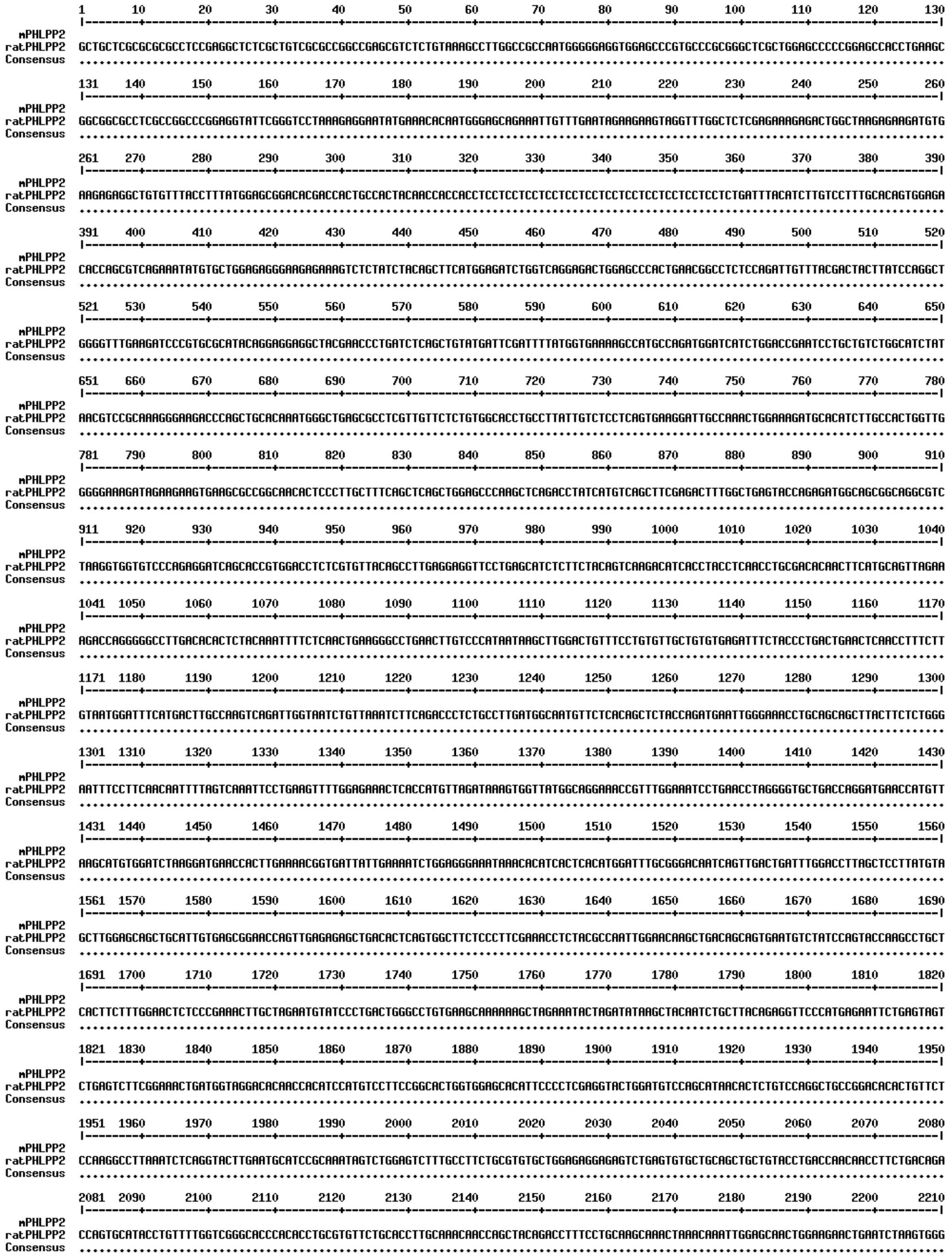

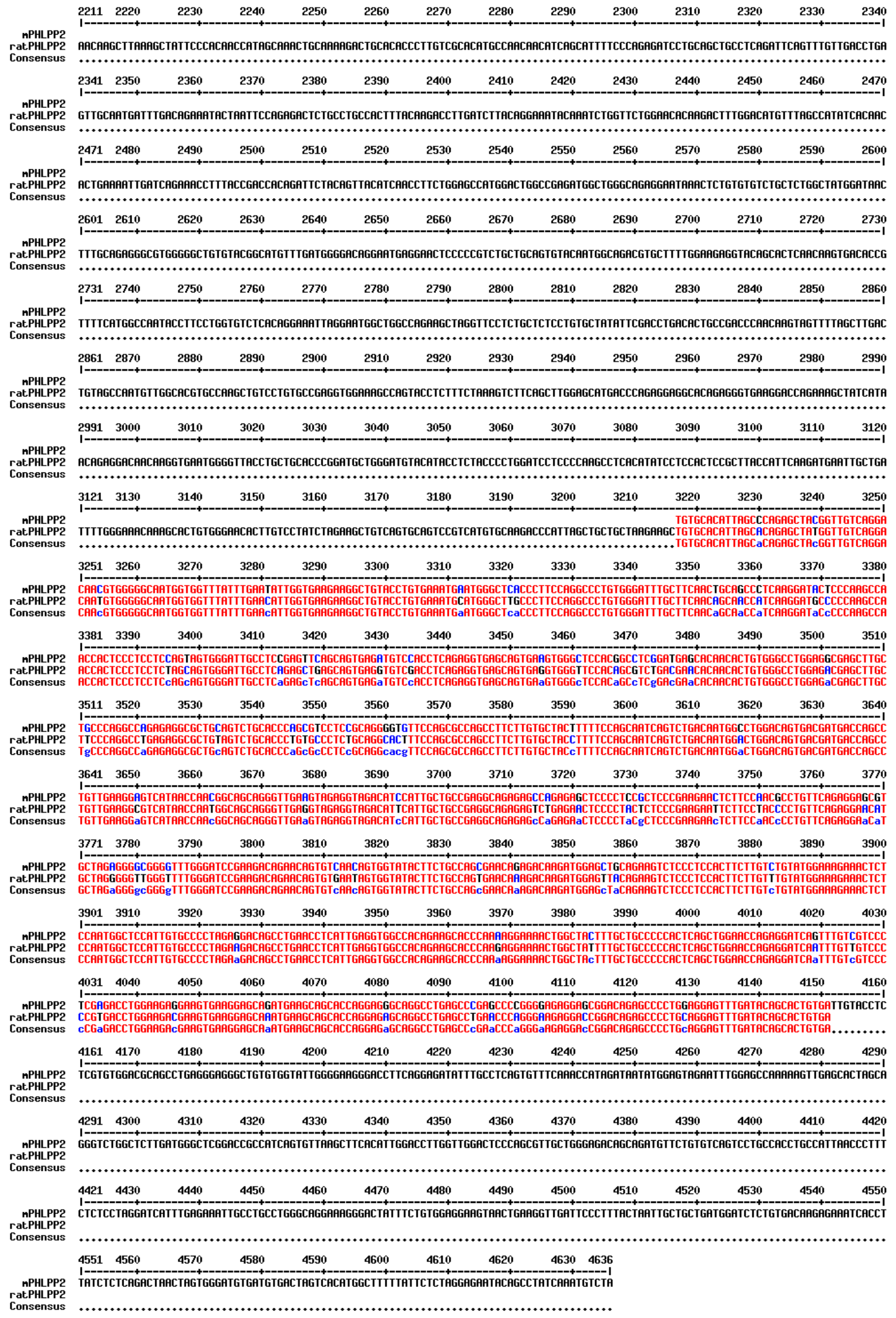

> *mmu*PHLPP2 (Reference Sequence: NM_001122594.2) (Species *Mus musculus*).

Control Amplicon primer F0/R0

<u>ATGTGCCAAGCTGTCCTGTG</u>CCGAGGTGGGAAGCCAGTACCTCTTTCTA

AAGTCTTCAGCTTGGAGCATGACCC

Targeting Amplicon primer F1/R1

Targeting Amplicon primer F3/R3

CCTGGACAGTGACGATGACCAGCCTGTTGAAGGAGTCATAACCAACGGCAGCAGG
GTTGAAGTAGAGGTAGACATCCATTGCTGCCGAGGCAGAGAGCCAGAGAGCTCCCC
TCCGCTCCCGAAGAACTCTTCCAACGCCTGTTCAGAGGAGCGTGCTAGAGGGGCGG
GGTTTGGGATCCGAAGACAGAACAGTGTCAACAGTGGTATACTTCTGCCAGCGAAC
AGAGACAAGATGGAGCTGCAGAAGTCTCCCTCCACTTCTTGTCTGTATGGAAAGAA
ACTCTCCAATGGCTCCATTGTGCCCCTAGAGGACAGCCTGAACCTCATTGAGGTGGC
CACAGAAGCACCCAAAAGGAAAACTGGCTACT

Targeting Amplicon primer F2/R2

Targeting Amplicon primer F4/R4

GAGCCCGAGCCCCGGGGAGAGGAGCGGACAGAGCCCCTGGAGGAGTTTGATACAG
CACTGTGATTGTACCTCTCGTGTGGACGCAGCCTGAGGGAGGGCTGTGTGGTATTGG
GGAAGGGACCTTCAGGAGATATTTGCCTCAGTGTTTCAAACCATAGATAATATGGAG
TAGAATTTGGAGCCAAAAAGTTGAGCACTAGCAGGGTCTGGCTCTTGATGGGCTCG
GACCGCCATCAGTGTTAAGCTTCACATTGGACCTTGGTTGGACTCCCAGCGTTGCTG
GGAGACAGCAGATGTTCTGTGTCAGTCCTGCCACCTGCCATTAACCCTTTCTCTCCT
AGGATCATTTGAGAAATTGCCTGCCTGGGCAGGAAAGGGACTATT

Supplementary Table 5. Related to method section.

Primers used in SELECT.

Name	Sequences (5’-->3’)
mPHLPP2-m^{6}A3675-up	tagccagtaccgtagtgcgtgCTGAACAGGCGTTGGAAGAG
mPHLPP2-m^{6}A3675-down	TCTTCGGGAGCGGAGGGGAGcagaggctgagtcgctgcat
mPHLPP2-m^{6}A3733-up	tagccagtaccgtagtgcgtg CCACTGTTGACACTGTTCTG
mPHLPP2-m^{6}A3733-down	CTTCGGATCCCAAACCCCGCcagaggctgagtcgctgcat
mPHLPP2-A155-up	tagccagtaccgtagtgcgtg GGAACCAAACCTACTTCTTC
mPHLPP2-A155-down	ATTCAAACAAGTTCTGCTCCcagaggctgagtcgctgcat
qPCR for SELECT-F	ATGCAGCGACTCAGCCTCTG
qPCR for SELECT-R	TAGCCAGTACCGTAGTGCGTG

Supplementary Table 6. Related to method section.

The shRNA sequences were used in this study as follows:

	sequence
METTL14 shRNA for NRCM	F: 5’-CCGGGCTAATGTTGACATTGACTTACTCGAGTAAGTCAATGTCAACATTAGCTTTTTG-3’<br>R: 5’-AATTCAAAAAGCTAATGTTGACATTGACTTACTCGAGTAAGTCAATGTCAACATTAGC-3’
METTL14 shRNA for murine	F: ggtaccAAGGTATATTGCTGTTGACAGTGAGCGCAGGGCTTCCTATGATACATCTGCTTAGTGAAGCCACAG<br>R: gaattcCGAGGCAGTAGGCACAGGGCTTCCTATGATACATCTGCTTACATCTGTGGCTTCACTAAGCAGATG
PHLPP2 shRNA for NRCM	F: 5’-CCGGAGACACCAGCGTCAGAAATATCTCGAGATATTTCTGACGCTGGTGTCTTTTTTG-3’<br>R: 5’-AATTCAAAAAAGACACCAGCGTCAGAAATATCTCGAGATATTTCTGACGCTGGTGTCT-3’

Supplementary Table 7. Related to method section.

The primer sequences used for generation of overexpression constructs were as follows:

	**Forward**	**Reverse**
For ratMettl14:		
ratMettl14-OE	ATGAGTTTGGGAGCTGAGAGT	CTACCGAGGTGTAAAGCCACCT
ratMettl14-op	gctgcaggtcgactctagagATGAGTTTGGGAGCTGAGAGT	gataagcttgatatcgaattCTACCGAGGTGTAAAGCCACCT
For ratPhlpp2:		
rat-Phlpp2-gene	F1: CGAGCGTCTCTGTAAAGCCT	R1: TGATGTTGTTGGCATGTGCG
	F2: GCATCCGCAAATAGTCTGGA	R2: TCACAGTGCTGTATCAAACTCCTGCA
		R3: TCCAGACTATTTGCGGATGC
ratPhlpp2-op	gtggaggtggaggttctagaAGCGTCTCTGTAAAGCCTTG	tcagcggtttaaacttaagcttgTCACAGTGCTGTATCAAACTCCTGCA

Supplementary Table 8. Related to method section.

Antibodies used in this study.

Antibodies	Source	Catalog	lot	Application
Rabbit polyclonal anti-ZC3H13	Abcam	Cat#ab70802	GR3235168-28	Western Blot
Rabbit monoclonal anti-C/EBPβ[E299]	Abcam	Cat#ab32358	GR3232277-8	Western Blot
Rabbit Polyclonal anti-PHLPP2	ABclonal	Cat#A18218	1167720201	Western Blot
Rabbit monoclonal anti-[KO Validated] active + pro Caspase-3[ARC0133]	ABclonal	Cat#A19654	4001330002	Western Blot
Rabbit monoclonal anti-Caspase-3 p12[ARC0143]	ABclonal	Cat#A19664	4000000143	Western Blot
Rabbit monoclonal anti-Bcl-2[ARC0173]	ABclonal	Cat#A19693	4000000173	Western Blot
Rabbit polyclonal anti-[KO Validated] Bax	ABclonal	Cat#A12009	5500008806	Western Blot
Rabbit polyclonal anti-PHLPP1	ABclonal	Cat#A9542	3521609005	Western Blot
Rabbit polyclonal anti-RBM15B	ABclonal	Cat#A9873	0051800201	Western Blot
Rabbit monoclonal anti-FOXO3A [75D8]	Beyotime Biotechnology	Cat#AF609	031221210930	Western Blot
Rabbit polyclonal Phospho-FoxO3a (Ser253) Antibody	Cell Signaling Technology	Cat #9466	6	Western Blot
Rabbit polyclonal GAPDH	Bioworld Technology	Cat#AP0063	01201908	Western Blot
Rabbit monoclonal anti-METTL14 [D8K8W]	Cell Signaling Technology	Cat#51104	1	Western Blot
Rabbit monoclonal anti-METTL3 [D2I6O]	Cell Signaling Technology	Cat#96391	1	Western Blot
Rabbit monoclonal	Cell Signaling	Cat#4060	38	Western Blot

anti-Phospho-Akt (Ser473) [D9E] XP	Technology			
Rabbit monoclonal anti-Phospho-Akt (Thr308)[D25E6]XP	Cell Signaling Technology	Cat#13038	18	Western Blot
Rabbit monoclonal anti-FTO [D2V1I]	Cell Signaling Technology	Cat#45980	1	Western Blot
Rabbit monoclonal anti-ALKBH5 [E5Y7C]	Cell Signaling Technology	Cat#80283	1	Western Blot
Rabbit monoclonal anti-Phospho-Akt2 (Ser474) [D3H2]	Cell Signaling Technology	Cat#8599	2	Western Blot
Mouse monoclonal anti-Akt2 [L79B2]	Cell Signaling Technology	Cat#5239	4	Western Blot
Rabbit monoclonal anti-Phospho-GSK-3β (Ser9) [D85E12] XP	Cell Signaling Technology	Cat#5558	10	Western Blot
Rabbit monoclonal anti-GSK-3β [D5C5Z] XP	Cell Signaling Technology	Cat#12456	8	Western Blot
Rabbit monoclonal anti-PI3 Kinase p110α [C73F8]	Cell Signaling Technology	Cat#4249	11	Western Blot
Rabbit Polyclonal WTAP polyclonal antibody	Proteintech	Cat#10200-1-AP	00048586	Western Blot
Rabbit Polyclonal AKT polyclonal antibody	Proteintech	Cat#10176-2-AP	00099352	Western Blot
Mouse Monoclonal Beta Actin antibody[2D4H5]	Proteintech	Cat#66009-1-Ig	10004156	Western Blot
Rabbit polyclonal IGF-1 antibody	Proteintech	Cat#28530-1-AP	00085177	Western Blot
Rabbit Polyclonal Phospho-Histone H3 (Ser10) antibody	Thermo Fisher Scientific	Cat#PA5-17869	WC3216175A	Immunofluorescence
Rabbit monoclonal Ki67 antibody [SP6]	Abcam	Cat#ab16667	GR3375640-18	Immunofluorescence
Monoclonal Anti-alpha-Actinin [EA-53] (Sarcomeric)	Sigma-Aldrich	Cat#A7811	0000120831	Immunofluorescence

antibody				
Cy3 AffiniPure Donkey Anti-Mouse IgG (H+L)	Jackson ImmunoResearch Labs	Cat#715-165-151	155867	Immunofluorescence
Alexa Fluor 488-conjugated Affinipure Goat Anti-Mouse IgG(H+L)	Jackson ImmunoResearch Labs	Cat#115-545-003	158218	Immunofluorescence
Alexa Fluor 488-AffiniPure Goat Anti-Rabbit IgG (H+L) antibody	Jackson ImmunoResearch Labs	Cat#111-545-003	146644	Immunofluorescence
Anti-m6A antibody	Synaptic Systems	Cat#202 003	2-125	Immunoprecipitation
Rabbit polyclonal anti-YTHDF1	Proteintech	Cat#17479-1-AP	00102690	Immunoprecipitation
Normal Rabbit-IgG	Sigma-Aldrich	Cat#NI01	3238429	Immunoprecipitation
Rabbit polyclonal anti-METTL14	ABclonal	Cat#A8530	5500003462	Immunoprecipitation

Supplementary Table 9. Related to method section.

***mmu*PHLPP2-WT-pGL3**

Cloning vector (Insert site): pGL3-Basic(gccgtg/taattc)

>*mmu*PHLPP2-WT-pGL3

GGAGGCGGCGGAAGCTCTGACAATGGCCTGGACAGTGACGATGACCAGCCTGTTG
AAGGAGTCATAACCAACGGCAGCAGGGTTGAAGTAGAGGTAGACATCCATTGCTGC
CGAGGCAGAGAGCCAGAGAGCTCCCCTCCGCTCCCGAAGAACTCTTCCAACGCCTG
TTCAGAGGAGCGTGCTAGAGGGGCGGGGTTTGGGATCCGAAGACAGAACAGTGTC
AACAGTGGTATACTTCTGCCAGCGAACAGAGACAAGATGGAGCTGCAGAAGTCTCC
CTCCACTTCTTGTCTGTATGGAAAGAAACTCTCCAATGGCTCCATTGTGCCCCTAGA
GGACAGCCTGAACCTCATTGAGGTGGCCACAGAAGCACCCAAAAGGAAAACTGGC
TACTTTGCTGCCCCCACTCAGCTGGAACCAGAGGATCAGTTTGTCGTCCCTCGAGAC
CTGGAAGAGGAAGTGAAGGAGCAGATGAAGCAGCACCAGGAGGGCAGGCCTGAG
CCCGAGCCCCGGGGAGAGGAGCGGACAGAGCCCCTGGAGGAGTTTGATACAGCAC
TGTGATTGTACCTCTCGTGTGGACGCAGCCTGAGGGAGGGCTGTGTGGTATTGGGG
AAGGGACCTTCAGGAGATATTTGCCTCAGTGTTTCAAACCATAGATAATATGGAGTA
GAATTTGGAGCCAAAAAGTTGAGCACTAGCAGGGTCTGGCTCTTGATGGGCTCGGA
CCGCCATCAGTGTTAAGCTTCACATTGGACCTTGGTTGGACTCCCAGCGTTGCTGGG
AGACAGCAGATGTTCTGTGTCAGTCCTGCCACCTGCCATTAACCCTTTCTCTCCTAG
GATCATTTGAGAAATTGCCTGCCTGGGCAGGAAAGGGACTATTTCTGTGGAGGAAG
TAACTGAAGGTTGATTCCCTTTACTAATTGCTGCTGATGGATCTCTGTGACAAGAGA
AATCACCTTATCTCTCAGACTAACTAGTGGGATGTGATGTGACTAGTCACATGGCTTT
TTATTCTCTAGGAGAATACAGCCTATCAAATGTCTA

***mmu*PHLPP2-mut-pGL3**

Cloning vector (Insert site): pGL3-Basic(gccgtg/taattc)

>*mmu*PHLPP2-mut-pGL3

GGAGGCGGCGGAAGCTCTGACAATGGCCTGGTCAGTGACGATGACCAGCCTGTTGA
AGGAGTCATAACCAACGGCAGCAGGGTTGAAGTAGAGGTAGTCATCCATTGCTGCC
GAGGCAGAGAGCCAGAGAGCTCCCCTCCGCTCCCGAAGATCTCTTCCAACGCCTGT
TCAGAGGAGCGTGCTAGAGGGGCGGGGTTTGGGATCCGAAGTCAGATCAGTGTCAA
CAGTGGTATACTTCTGCCAGCGATCAGAGTCAAGATGGAGCTGCAGAAGTCTCCCTC
CACTTCTTGTCTGTATGGAAAGAATCTCTCCAATGGCTCCATTGTGCCCCTAGAGGTC
AGCCTGATCCTCATTGAGGTGGCCACAGAAGCACCCAAAAGGAAATCTGGCTACTT
TGCTGCCCCCACTCAGCTGGATCCAGAGGATCAGTTTGTCGTCCCTCGAGTCCTGGA
AGAGGAAGTGAAGGAGCAGATGAAGCAGCACCAGGAGGGCAGGCCTGAGCCCGA
GCCCCGGGGAGAGGAGCGGTCAGAGCCCCTGGAGGAGTTTGATACAGCACTGTGAT
TGTACCTCTCGTGTGGACGCAGCCTGAGGGAGGGCTGTGTGGTATTGGGGAAGGGT
CCTTCAGGAGATATTTGCCTCAGTGTTTCAATCCATAGATAATATGGAGTAGAATTTG
GAGCCAAAAAGTTGAGCACTAGCAGGGTCTGGCTCTTGATGGGCTCGGTCCGCCAT
CAGTGTTAAGCTTCACATTGGTCCTTGGTTGGTCTCCCAGCGTTGCTGGGAGTCAGC
AGATGTTCTGTGTCAGTCCTGCCACCTGCCATTAACCCTTTCTCTCCTAGGATCATTT
GAGAAATTGCCTGCCTGGGCAGGAAAGGGTCTATTTCTGTGGAGGAAGTAACTGAA
GGTTGATTCCCTTTACTAATTGCTGCTGATGGATCTCTGTGACAAGAGAAATCACCTT

ATCTCTCAGTCTAACTAGTGGGATGTGATGTGACTAGTCACATGGCTTTTTATTCTCT
AGGAGAATACAGCCTATCAAATGTCTA

***mmu*PHLPP1-WT-pGL3**

Cloning vector (Insert site): pGL3-Basic(gccgtg/taattc)

>*mmu*PHLPP1-WT-pGL3

GGAGGCGGCGGAAGCGGCATGGCATCTGAGATCAGCAGTGAACTGTCTACCTCCGA
GATGAGTAGTGAGGTGGGCTCCACAGCCTCTGATGAGCCCCCGTCTGGAGTCCTGA
ATGAGAGCAGCCCTGCCTACCCCAACGAGCAGCGCTGCATGCTCCACCCTGTCTGC
CTGTCTAACTCCTTCCAACGTCAGCTGTCCAGCGCTACTTTCTCCAGCGCGTTCTCT
GACAACGGCCTTGACAGTGACGATGAAGAACCCATTGAGGGGGTGTTCAGCAACG
GCAGCCGGGTTGAGGTGGAAGTAGACATCCACTGCAGCAGGGCCAAGGAAAAGGA
GAGACAGCAGCACCTACTTCAGGTGCCAGCTGAGGCCAGTGATGAGGGCATTGTCA
TCAGTGCCAATGAGGATGAGTCAGGTCTGTCCAAGAAGGCAGACTTTTCTGCTGTG
GGAACCATTGGACGACGCAGGGCTAATGGCTCTGTAGCTCCCCAGGAAAGGAGCCA
TAATGTGATAGAGGTTGCTGCAGATGCGCCTCTCCGGAAGCCAGGAGGCTATTTTGC
AGCCCCTGCTCAGCCAGATCCAGATGATCAGTTTATCATCCCCCCAGAGCTGGAAGA
GGAAGTCAAAGAAATCATGAAACATCACCAGGAGCAGCAGCAGCAGCAGCAGCAG
CAGCAGCTGCCACCACCACCCCAGCCGCCACAGCCACAGCCACAGCCACAGCCAC
AGCCACAGCCACAGCCACAGCGGCACTTCCAAATGGATCACCTGCCAGACTGTTAC
GATACACCGCTATGACCCAGCCTGAGTACTGTTTTAAACAATAAACTAACCAGAGAG
ACTGAGAGTCTCCCAGGCTCACATTAAACCAGGGGTGTTAACTTCATGCCCTCTCCC
AGACACTGCCCCACCTGTCTCTGCAGCTCATCTGTAGGTTCTCTTTGTATTGGTTACT
TTTAACAATTAACCACTTTTCTTCTAGTGATACTTTACCAATATGATTTAAATGTGCAG
ATAATGTGGCCCTGTTCTCGGGAACCCAGAACCTTTTACAGACATTTTTTAATTGTGT
TCCTTCCTGCTGCCACAATCAGCATGACTGTATAGAGTCCCTTATACCATCTACACAT
CCAGAGTTATATGGAAGCAGAATGCCC

***mmu*PHLPP1-mut-pGL3**

Cloning vector (Insert site): pGL3-Basic(gccgtg/taattc)

>*mmu*PHLPP1-mut-pGL3

GGAGGCGGCGGAAGCGGCATGGCATCTGAGATCAGCAGTGATCTGTCTACCTCCGA
GATGAGTAGTGAGGTGGGCTCCACAGCCTCTGATGAGCCCCCGTCTGGAGTCCTGA
ATGAGAGCAGCCCTGCCTACCCCAACGAGCAGCGCTGCATGCTCCACCCTGTCTGC
CTGTCTAACTCCTTCCAACGTCAGCTGTCCAGCGCTACTTTCTCCAGCGCGTTCTCT
GACAACGGCCTTGACAGTGACGATGAAGATCCCATTGAGGGGGTGTTCAGCAACGG
CAGCCGGGTTGAGGTGGAAGTAGTCATCCACTGCAGCAGGGCCAAGGAAAAGGAG
AGTCAGCAGCACCTACTTCAGGTGCCAGCTGAGGCCAGTGATGAGGGCATTGTCAT
CAGTGCCAATGAGGATGAGTCAGGTCTGTCCAAGAAGGCAGTCTTTTCTGCTGTGG
GAACCATTGGACGACGCAGGGCTAATGGCTCTGTAGCTCCCCTGGAAAGGAGCCAT
AATGTGATAGAGGTTGCTGCAGATGCGCCTCTCCGGAAGCCAGGAGGCTATTTTGCA
GCCCCTGCTCAGCCAGATCCAGATGATCAGTTTATCATCCCCCCAGAGCTGGAAGAG
GAAGTCAAAGAAATCATGAATCATCACCAGGAGCAGCAGCAGCAGCAGCAGCAGC
AGCAGCTGCCACCACCACCCCAGCCGCCACAGCCACAGCCACAGCCACAGCCACA
GCCACAGCCACAGCCACAGCGGCACTTCCAAATGGATCACCTGCCAGTCTGTTACG
ATACACCGCTATGACCCAGCCTGAGTACTGTTTTAATCAATAATCTAACCAGAGAGTC

TGAGAGTCTCCCAGGCTCACATTAATCCAGGGGTGTTAACTTCATGCCCTCTCCCAG TCACTGCCCCACCTGTCTCTGCAGCTCATCTGTAGGTTCTCTTTGTATTGGTTACTTTT AACAATTAACCACTTTTCTTCTAGTGATACTTTACCAATATGATTTAAATGTGCAGATA ATGTGGCCCTGTTCTCGGGATCCCAGATCCTTTTACAGTCATTTTTTAATTGTGTTCCT TCCTGCTGCCACAATCAGCATGACTGTATAGAGTCCCTTATACCATCTACACATCCAG AGTTATATGGAAGCAGAATGCCC
***Rattus* PHLPP2-WT-pGL3**<br>Cloning vector (Insert site): pGL3-Basic(gccgtg/taattc)<br>>*Rattus* PHLPP2-WT-pGL3<br>GGAGGCGGCGGAAGCTCTGACAATGGACTGGACAGTGACGATGACCAGCCTGTTG AAGGCGTCATAACCAATGGCAGCAGGGTTGAGGTAGAGGTAGACATTCATTGCTGC CGAGGCAGAGAGTCTGAGAACTCCCCTACTCTCCCGAAGAATTCTTCCTACCCCTGT TCAGAGGAACATGCTAGGGGGTTGGGTTTTGGGATCCGAAGACAGAACAGTGTGAA TAGTGGTATACTTCTGCCAGTGAACAAAGACAAGATGGAGTTACAGAAGTCTCCCTC CACTTCTTGTTTGTATGGAAAGAAACTCTCCAATGGCTCCATTGTGCCCCTAGAAGA CAGCCTGAACCTCATTGAGGTGGCCACAGAAGCACCCAAGAGGAAAACTGGCTATT TTGCTGCCCCCACTCAGCTGGAACCAGAGGATCAATTTGTTGTCCCCCGTGACCTGG AAGACGAAGTGAAGGAGCAAATGAAGCAGCACCAGGAGAGCAGGCCTGAGCCTG AACCCAGGGAAGAGGACCGGACAGAGCCCCTGCAGGAGTTTGATACAGCACTGTG A
***Rattus* PHLPP2-mut-pGL3**<br>Cloning vector (Insert site): pGL3-Basic(gccgtg/taattc)<br>>*Rattus* PHLPP2-mut-pGL3<br>GGAGGCGGCGGAAGCTCTGACAATGGTCTGGTCAGTGACGATGACCAGCCTGTTGA AGGCGTCATAACCAATGGCAGCAGGGTTGAGGTAGAGGTAGTCATTCATTGCTGCCG AGGCAGAGAGTCTGAGATCTCCCCTACTCTCCCGAAGAATTCTTCCTACCCCTGTTC AGAGGATCATGCTAGGGGGTTGGGTTTTGGGATCCGAAGTCAGATCAGTGTGAATAG TGGTATACTTCTGCCAGTGATCAAAGTCAAGATGGAGTTACAGAAGTCTCCCTCCAC TTCTTGTTTGTATGGAAAGAATCTCTCCAATGGCTCCATTGTGCCCCTAGAAGTCAGC CTGATCCTCATTGAGGTGGCCACAGAAGCACCCAAGAGGAAATCTGGCTATTTTGCT GCCCCCACTCAGCTGGAACCAGAGGATCAATTTGTTGTCCCCCGTGACCTGGAAGA CGAAGTGAAGGAGCAAATGAAGCAGCACCAGGAGAGCAGGCCTGAGCCTGATCCC AGGGAAGAGGTCCGGTCAGAGCCCCTGCAGGAGTTTGATACAGCACTGTGA

Supplementary Figures

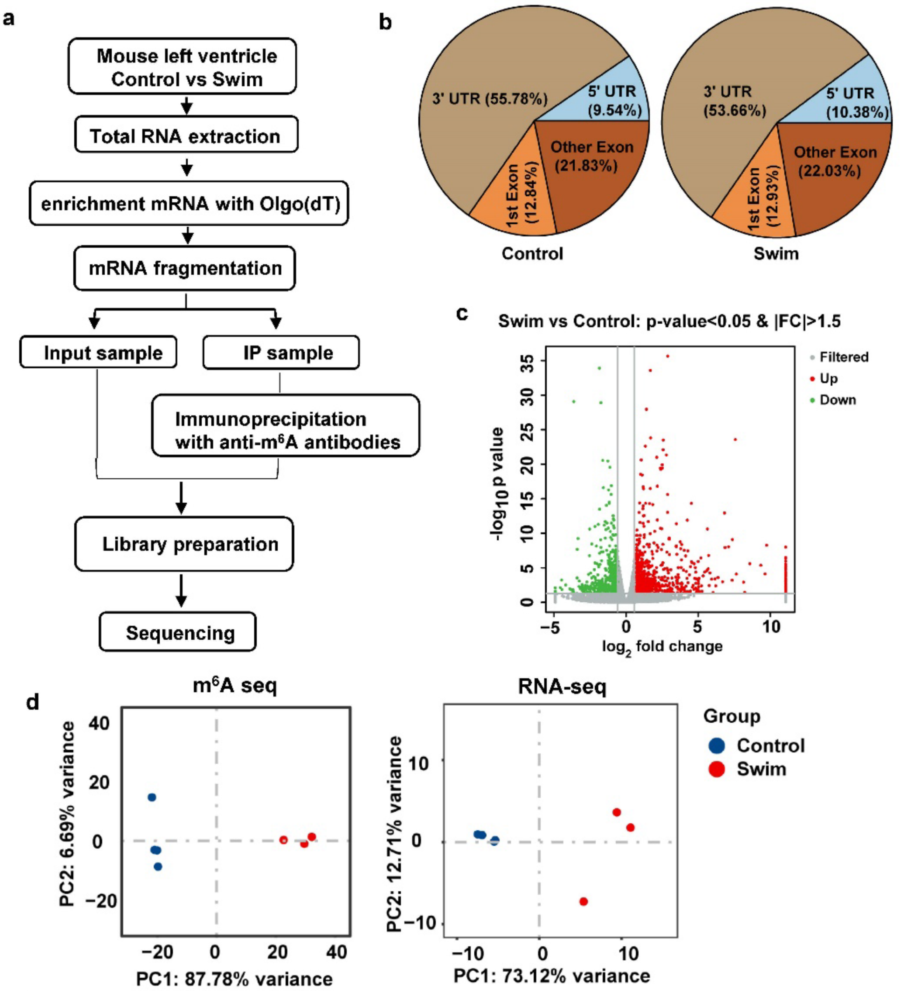

e

Differentially methylated peaks	swim-vs-control
Number of Peaks	475
Number of genes	436
Total Length of Peaks(bp)	1252289
Average Length of Peaks(bp)	2636.4
Median Length of Peaks(bp)	299
Minimum Length of Peaks(bp)	140
Maximum Length of Peaks(bp)	284643
Percentage of Genome(%)	0.05

Supplementary Fig. 1: Identifying m^{6}A RNA methylome of the exercised murine hearts by MeRIP-seq and RNA-seq. (**a**) Diagram of meRIP-seq experiment. (**b**) Pie charts showing the distribution of m^{6}A RNA methylation in the swim and control murine hearts, respectively. (**c**) Volcano plot of the RNA-seq. Transcripts with significantly increased peak (red) and decreased peak (green) changes are highlighted (*p*-value<0.05, fold change>1.5). Negative binomial test (two-sided). (**d**) PCA plots of MeRIP-seq and RNA-seq. (**e**) Statistics of the differential methylation peaks which were detected by meRIP in the swim and control hearts. Seq, sequencing. IP, immunoprecipitation.

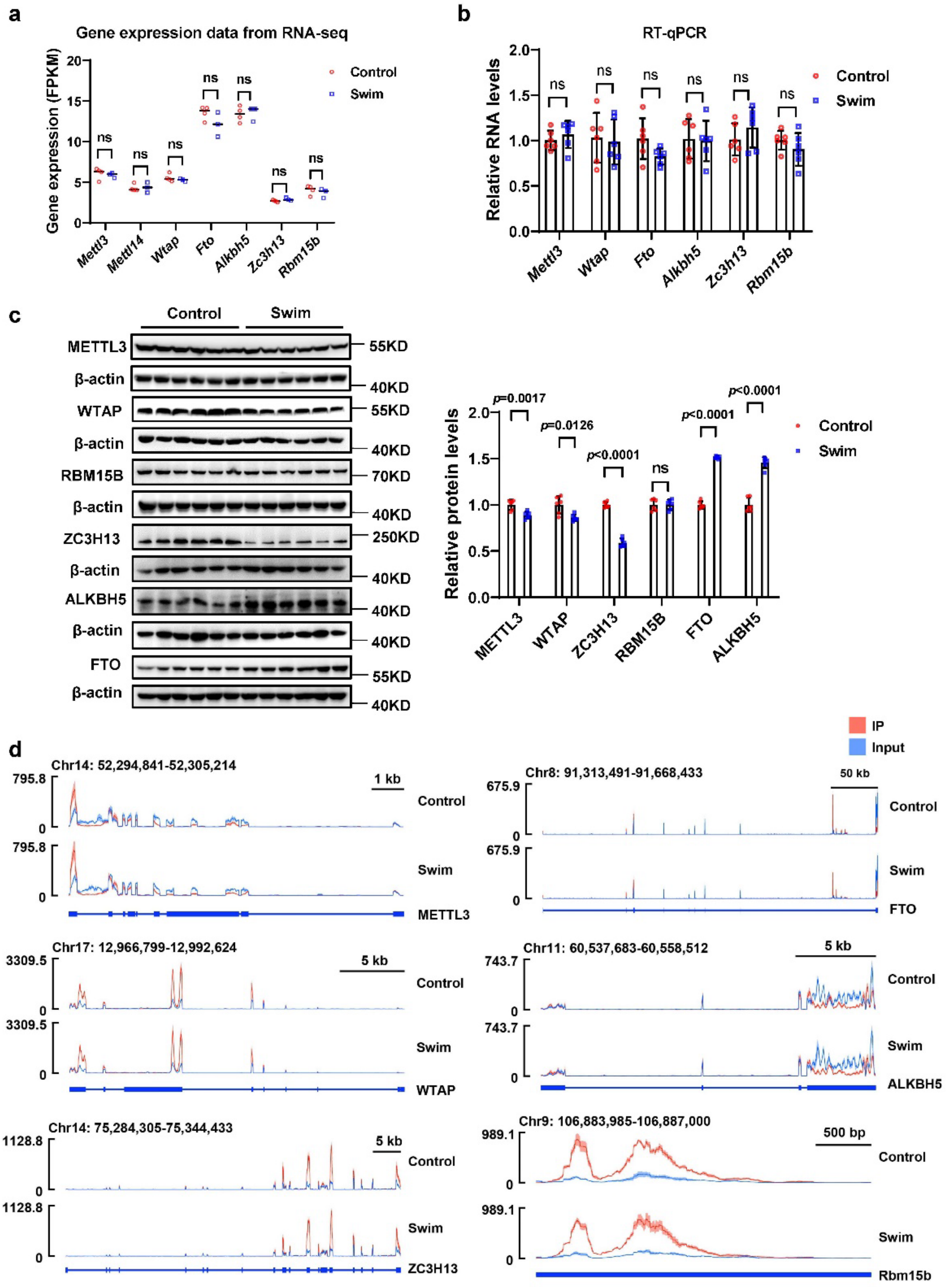

e

chr	chromStart	chromEnd	Name	diff.lg(p)	diff.log$_2$(FC)
Chr3	123368443	123368741	*Mettl14*	-4.11	-0.749
Chr3	123380298	123383616	*Mettl14*	-1.45	-0.719

Supplementary Fig. 2: The regulation of m^{6}A effectors in response to exercise. (**a**) Gene expression data of m^{6}A effectors (METTL3, METTL14, WTAP, FTO, ALKBH5, ZC3H13, and RBM15B) from RNA-seq (n=4:3 independent biological samples). (**b**) qPCR analysis of *Mettl3*, *Wtap*, *Fto*, *Alkbh5*, *Zc3h13*, and *Rbm15b* mRNA expression levels in the hearts with or without swim training (n=6 mice/group). (**c**) Representative western blot and statistical data displaying the knockdown of m^{6}A effectors (METTL3, WTAP, FTO, ALKBH5, ZC3H13, and

RBM15B) in whole lysates isolated from the hearts of swim and control murine (n=6 mice/group). (**d**) Integrative genomics viewer (IGV) tracks revealing the results of meRIP-seq (Red) and RNA-seq (Blue) reads distributions in *Mettl3*, *Wtap*, *Fto*, *Alkbh5*, *Zc3h13*, and *Rbm15b* mRNA of the swim and control. Seq, sequencing. (**e**) m^6A peaks on *Mettl14* mRNA in the hearts with or without swim training determined using meRIP-seq. IP, immunoprecipitation. RT-qPCR, quantitative reverse transcription polymerase chain reaction. ns, nonstatistically significant. All data are expressed as means ± SD. (**b** and **c**, independent-sample t-test, two-sided.) Source data are provided as a Source Data file.

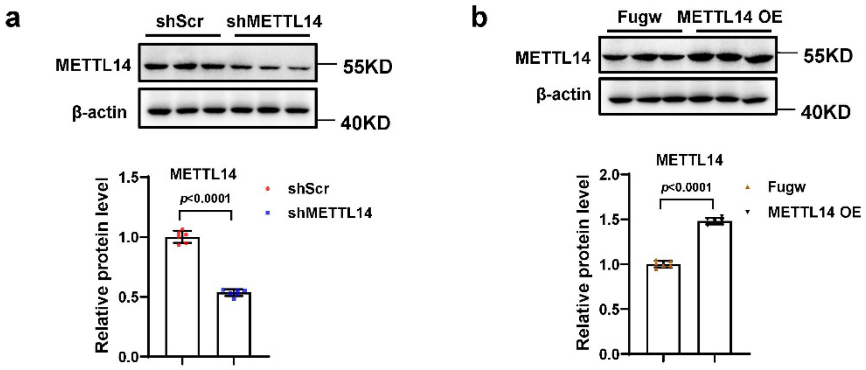

Supplementary Fig. 3: Overexpression and inhibition of METTL14 in NRCMs. (**a**) Representative western blot and statistical data displaying the knockdown of METTL14 in NRCMs by shMETTL14 (n=6 wells/group). (**b**) Representative western blot and statistical data displaying the overexpression of METTL14 in NRCMs via transfecting with METTL14 overexpression construct (n=6 wells/group). NRCM, neonatal rat cardiomyocyte. Fugw, control without METTL14 overexpression; METTL14 OE, METTL14 overexpression; shScr, Scramble short hairpin RNA; shMETTL14, METTL14 short hairpin RNA to knockdown METTL14. All data are expressed as means ± SD. (**a** and **b**, independent-sample t-test, two-sided.) Source data are provided as a Source Data file.

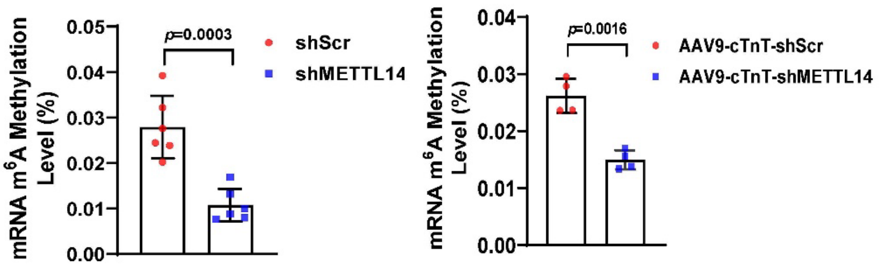

Supplementary Fig. 4: METTL14 inhibition reduces the mRNA m^{6}A level in NRCM. The relative mRNA m^{6}A level with or without METTL14 knockdown in NRCM (n=6 /group) or mice hearts (n=4 /group). NRCM, neonatal rat cardiomyocyte. shScr, Scramble short hairpin RNA; shMETTL14, METTL14 short hairpin RNA to knockdown METTL14; AAV9-cTnT-shScr, cardiac-specific troponin-T promoter-driven Scramble AAV9; AAV9-cTnT-shMETTL14, cardiac-specific troponin-T promoter-driven METTL14 knockdown AAV9. All data are expressed as means ± SD. (Independent-sample t-test, two-sided.) Source data are provided as a Source Data file.

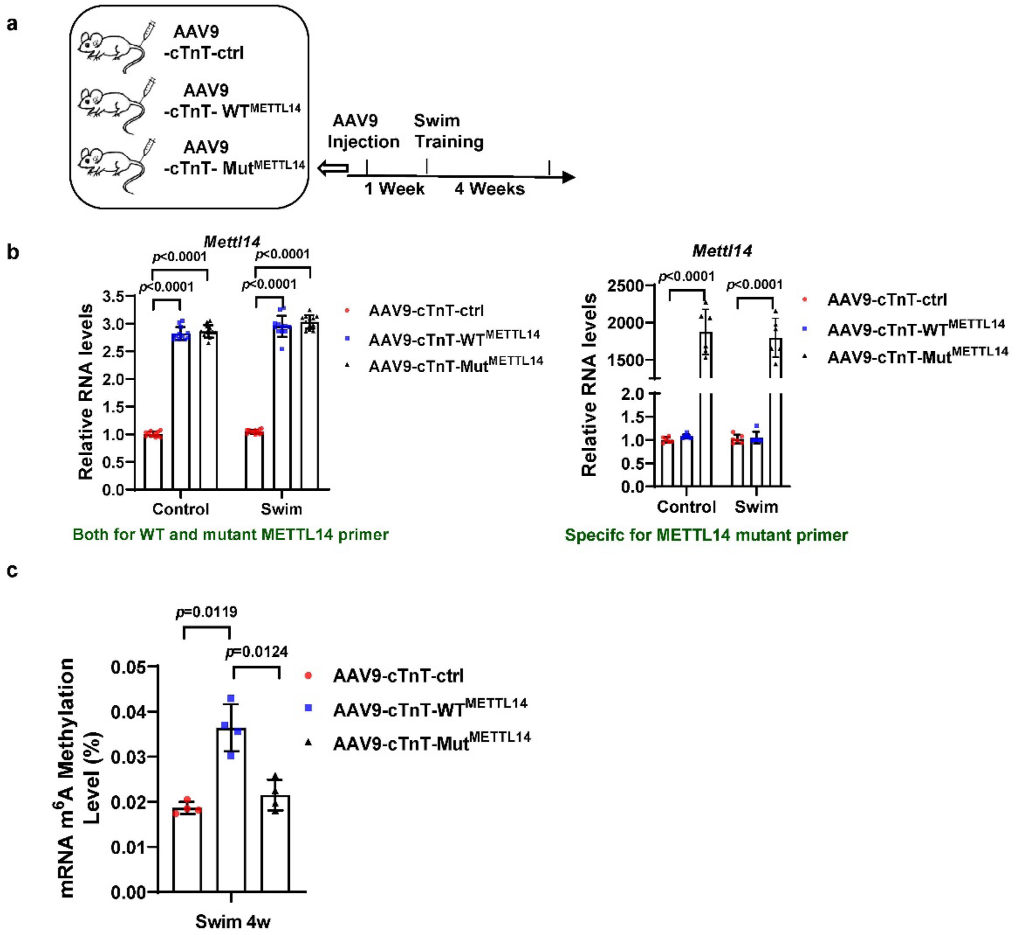

Supplementary Fig. 5: Mutant METTL14 abolishes the cardiac m^{6}A methylation elevation after swimming training. (**a**) The schedule of virus injection and swim-induced mice physiological cardiac hypertrophy model establishment. WT^{METTL14}, wild -type METTL14 overexpression. Mut^{METTL14}, METTL14 R254/R255A-R298P-D312A mutant overexpression. (**b**) qPCR analysis of *Mettl14* mRNA expression levels in mice hearts treated as indicated groups. Left panel, primer, which was used to PCR, for both WT and mutant METTL14 (n=12 /group). Right panel, primer, which was used to PCR, specific for mutant METTL14 (n=6 /group). (**c**) The relative mRNA m^{6}A level in hearts treated as indicated (n=4 /group). AAV9-cTnT-ctrl, cardiac-specific troponin-T promoter-driven control AAV9; AAV9-cTnT-WT^{METTL14}, cardiac-specific troponin-T promoter-driven wild-type METTL14 overexpression AAV9; AAV9-cTnT- Mut^{METTL14}, cardiac-specific troponin-T promoter-driven METTL14 R254/R255A-R298P-D312A mutant overexpression AAV9. All data are expressed as means ± SD. (**b**, two-way ANOVA followed by Tukey's post hoc test; **c**, one-way ANOVA followed by Bonferroni test.) Source data are provided as a Source Data file.

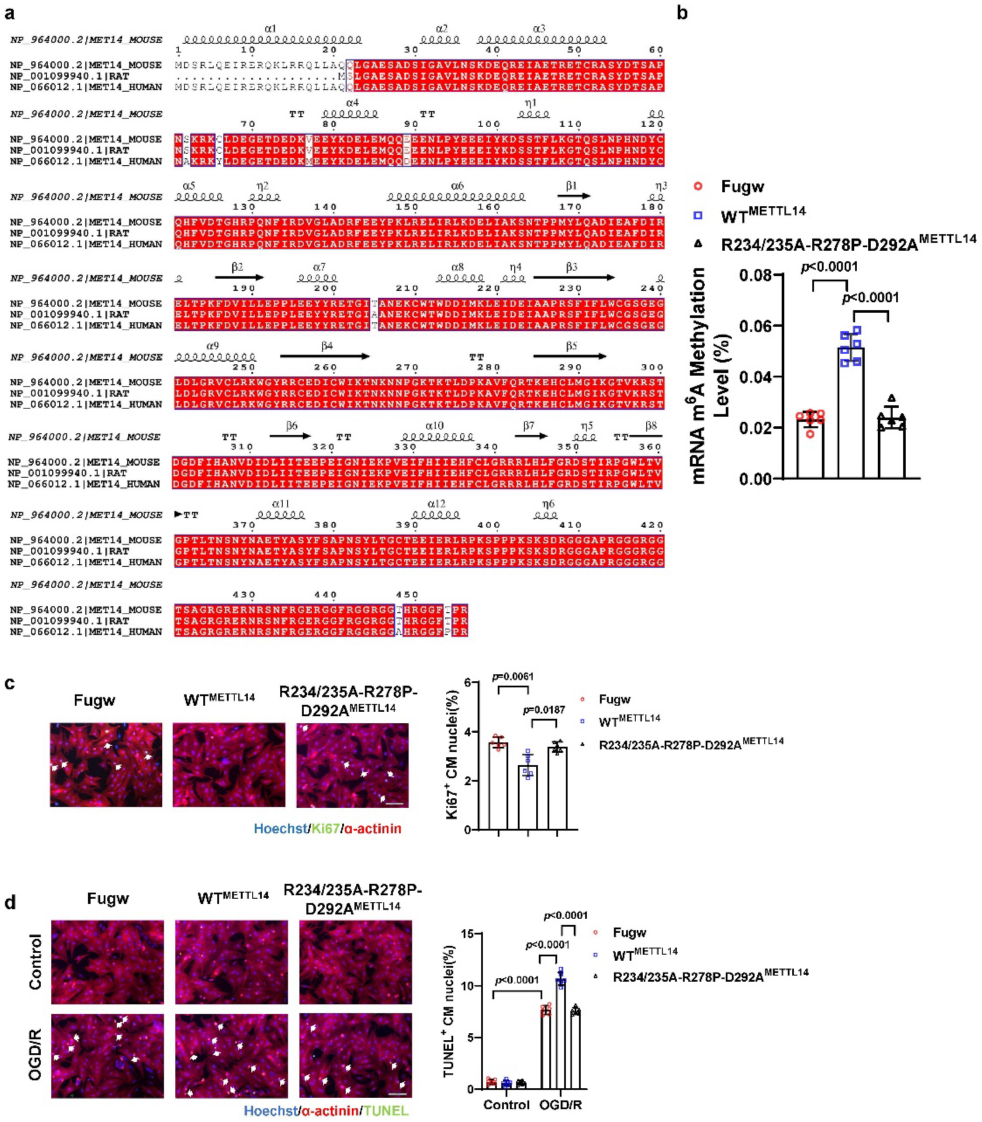

Supplementary Fig. 6: The pro-apoptosis and antiproliferative effects of $\text{WT}^{\text{METTL14}}$ overexpression on NRCM are disrupted by $\text{mut}^{\text{METTL14}}$ overexpression. (**a**) Protein alignment of METTL14 across mammalian species. Protein alignment was generated by CLUSTALW and ESPript 3.0. PDB file of murine METTL14 was download from AlphaFold Protein Structure Database. (**b**) The relative mRNA m^{6}A level in NRCM treated as indicated (n=6 /group). (**c**) Representative images of immunofluorescence staining and quantification of the Ki67 positive cardiomyocytes treated as indicated (n=6 wells/group). Scale bar: 100 μm. (**d**) Representative images of immunofluorescence staining and quantification of the TUNEL positive cardiomyocytes treated as indicated (n=6 wells/group). Scale bar: 100 μm. NRCM, neonatal rat cardiomyocyte. OGD/R, oxygen-glucose deprivation/reperfusion. Fugw, control without METTL14 overexpression; $\text{WT}^{\text{METTL14}}$, NRCM treated with wild-type METTL14 overexpression; $\text{R234/235A-R278P-D292A}^{\text{METTL14}}$, NRCM treated with METTL14 mutant overexpression. All data are expressed as means ± SD. (**b**, one-way ANOVA followed by Bonferroni test; **c**, one-way ANOVA followed by Dunnett T3 test; **d**, two-way ANOVA followed by Tukey's post hoc test.) Source data are provided as a Source Data file.

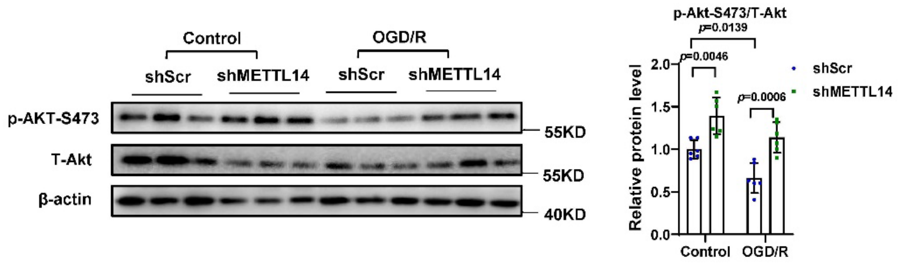

Supplementary Fig. 7: The activation of Akt-S473 presented in OGD/R treated NRCM is regulated by METTL14. Representative western blot and statistical data of the phosphorylation levels of Akt-S473 in OGD/R induced NRCM apoptosis model treated with shMETTL14 or scrambled control (n=6 wells/group). NRCM, neonatal rat cardiomyocyte. shScr, Scramble short hairpin RNA; shMETTL14, METTL14 short hairpin RNA to knockdown METTL14; OGD/R, oxygen-glucose deprivation/reperfusion. All data are expressed as means ± SD. (Two-way ANOVA followed by Tukey's post hoc test.) Source data are provided as a Source Data file.

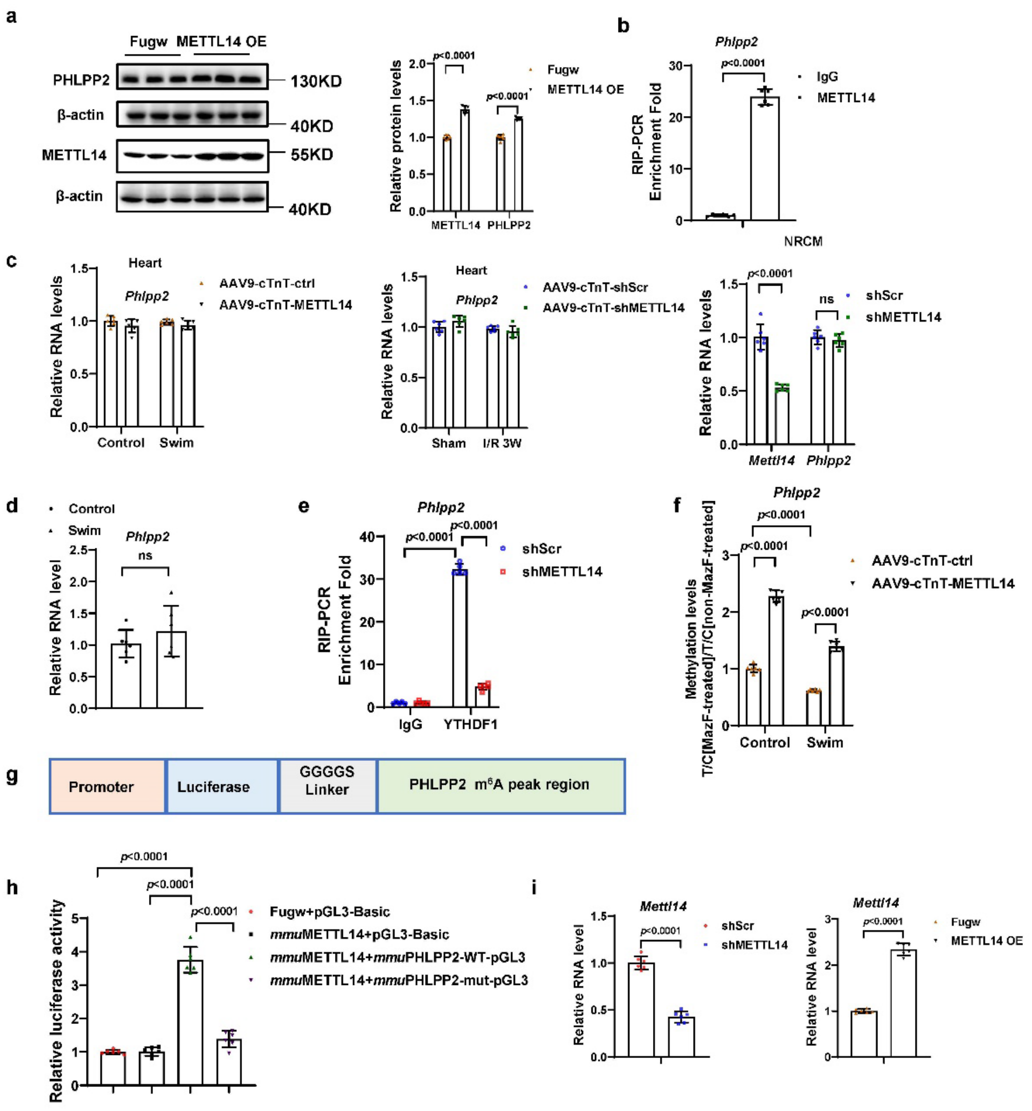

Supplementary Fig. 8: The regulation of METTL14 on PHLPP2. (**a**) Western blot analyses of PHLPP2 and METTL14 in NRCM with or without METTL14 overexpression (n=6 wells/group). (**b**) Validation of *Phlpp2* mRNA binding to METTL14 in H9C2 cardiomyocytes using RIP-qPCR (n=6/group). (**c**) qPCR analysis of *Phlpp2* mRNA expression levels in the hearts or NRCM treated as indicated (n=6 mice or wells/group). (**d**) qPCR analysis of *Phlpp2* mRNA expression levels in the hearts with or without swimming training (n=6 mice/group). (**e**) The binding of *Phlpp2* mRNA to YTHDF1 in H9C2 cardiomyocytes with or without METTL14 knockdown (n=5/group). (**f**) The methylation levels of *Phlpp2* mRNA in mice hearts treated as indicated measured by MazF-qPCR (n=6 mice/group). (**g**) Scheme of WT-PHLPP2 or Mut-PHLPP2 luciferase reporter gene. (**h**) Relative luciferase activity of mouse PHLPP2 wild type (PHLPP2-WT-pGL3) or m^{6}A mutant (PHLPP2-mut-pGL3) reporter gene with or without mouse METTL14 overexpression (n=6/group). (**i**) qPCR analysis of *Mettl14* expression with METTL14 overexpression or knockdown, related to MazF-qPCR in Fig. 7k (n=6/group). NRCM, neonatal rat cardiomyocyte; RIP, RNA immunoprecipitation assay; Fugw, control without METTL14 overexpression; METTL14 OE, METTL14 overexpression; shScr, Scramble short hairpin RNA; shMETTL14, METTL14 short hairpin RNA to knockdown METTL14; AAV9-cTnT-ctrl, cardiac-specific troponin-T promoter-driven

control AAV9; AAV9-cTnT-METTL14, cardiac-specific troponin-T promoter-driven METTL14 overexpression AAV9; AAV9-cTnT-shScr, cardiac-specific troponin-T promoter-driven Scramble AAV9; AAV9-cTnT-shMETTL14, cardiac-specific troponin-T promoter-driven METTL14 knockdown AAV9. ns, nonstatistically significant. All data are expressed as means ± SD. (**a**-**b**, **d**, and **i**, independent-sample t-test, two-sided; **c**, **e**-**f**, two-way ANOVA followed by Tukey's post hoc test; **h**, one-way ANOVA followed by Dunnett T3 test.) Source data are provided as a Source Data file.

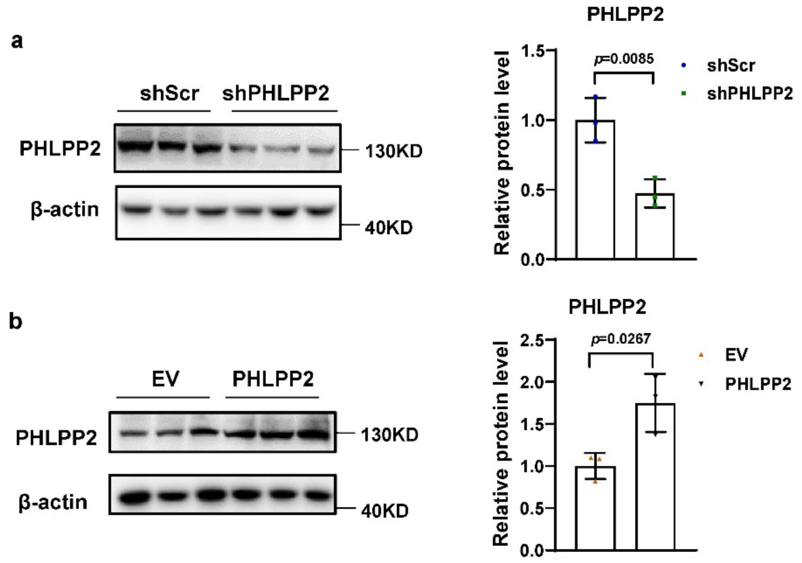

Supplementary Fig. 9: Overexpression and inhibition of PHLPP2 in NRCMs. (**a**) Representative western blot and statistical data displaying the knockdown of PHLPP2 in NRCMs by shPHLPP2 (n=3 wells/group). (**b**) Representative western blot and statistical data displaying the overexpression of PHLPP2 in NRCMs via transfecting with PHLPP2 overexpression construct (n=3 wells/group). NRCM, neonatal rat cardiomyocyte. EV, Empty vector without PHLPP2 overexpression; PHLPP2, PHLPP2 overexpression; shScr, Scramble short hairpin RNA; shPHLPP2, PHLPP2 short hairpin RNA to knockdown PHLPP2. All data are expressed as means ± SD. (**a** and **b**, independent-sample t-test, two-sided.) Source data are provided as a Source Data file.

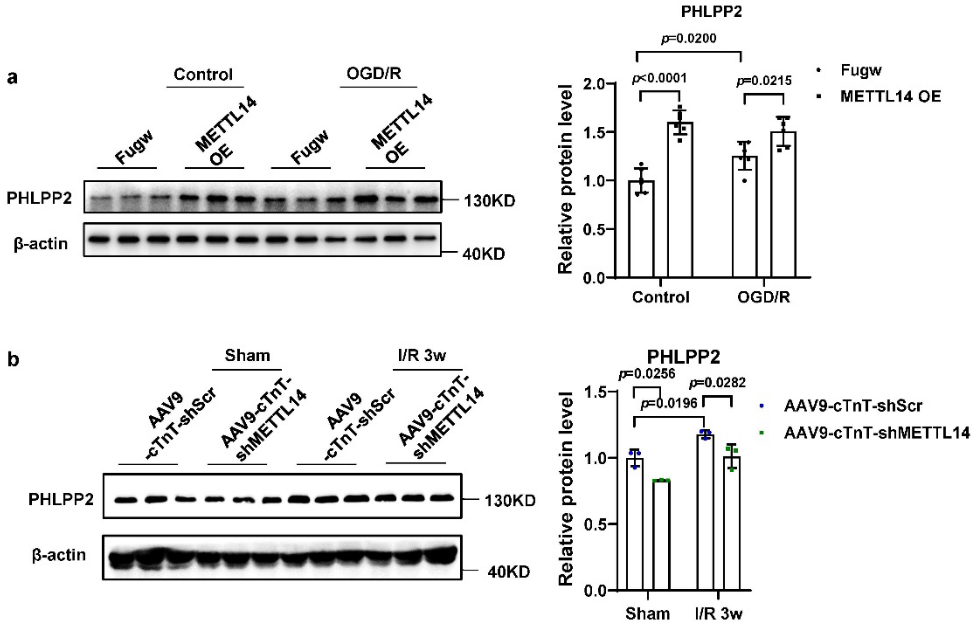

Supplementary Fig. 10: The expression of PHLPP2 were positively regulated by METTL14. (**a**) Representative western blot and statistical data of PHLPP2 expression levels in the NRCMs treated as indicated (n=6 wells /group). (**b**) Representative western blot and statistical data of PHLPP2 expression levels in the mice hearts treated as indicated (n=3 mice/group). NRCM, neonatal rat cardiomyocyte; OGD/R, oxygen-glucose deprivation/reperfusion; Fugw, control without METTL14 overexpression; METTL14 OE, METTL14 overexpression; AAV9-cTnT-shScr, cardiac-specific troponin-T promoter-driven Scramble AAV9; AAV9-cTnT-shMETTL14, cardiac-specific troponin-T promoter-driven METTL14 knockdown AAV9. All data are expressed as means ± SD. (**a** and **b**, Two-way ANOVA followed by Tukey's post hoc test.) Source data are provided as a Source Data file.

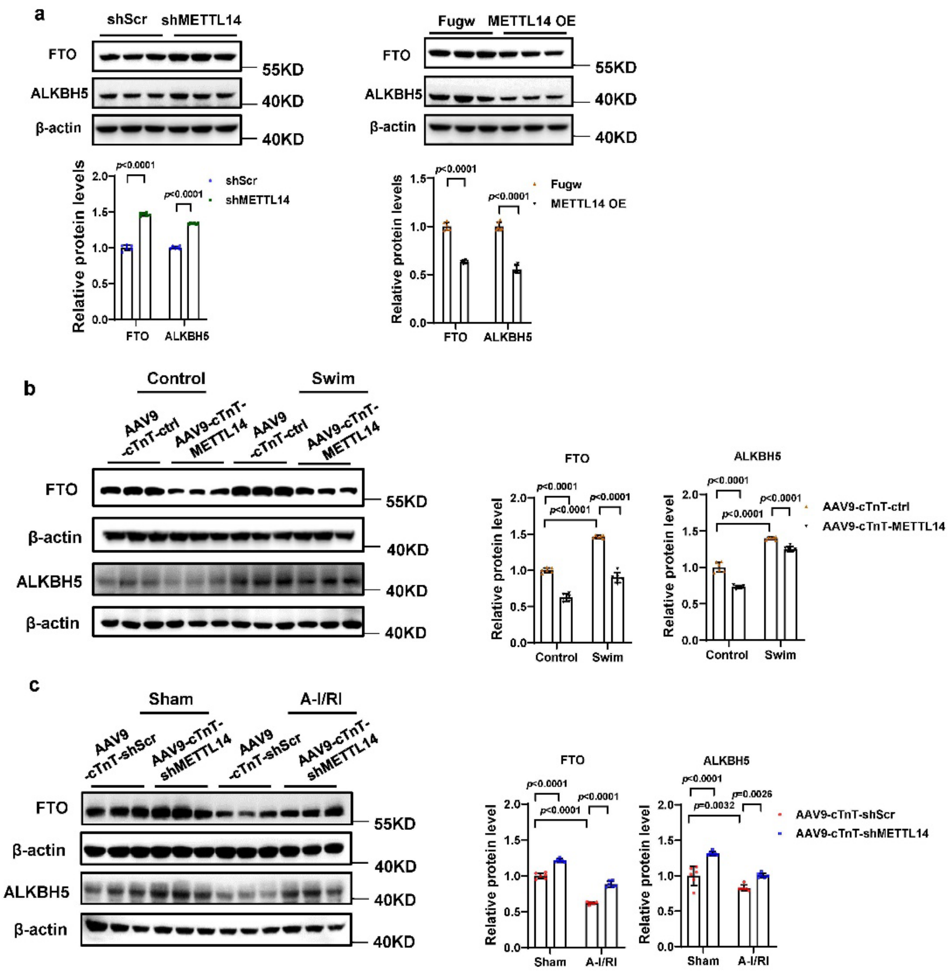

Supplementary Fig. 11: Demethylase FTO and ALKBH5 were negatively regulated by METTL14 alteration. (**a**) Representative western blot and statistical data of FTO and ALKBH5 in NRCMs with METTL14 knockdown or METTL14 overexpression (n=6 wells/group). (**b-c**) Representative western blot and statistical data of FTO and ALKBH5 expression levels in the mice hearts treated as indicated (n=6 mice/group). Fugw, control without METTL14 overexpression; METTL14 OE, METTL14 overexpression; shScr, Scramble short hairpin RNA; shMETTL14, METTL14 short hairpin RNA to knockdown METTL14; AAV9-cTnT-shScr, cardiac-specific troponin-T promoter-driven Scramble AAV9; AAV9-cTnT-shMETTL14, cardiac-specific troponin-T promoter-driven METTL14 knockdown AAV9; AAV9-cTnT-ctrl, cardiac-specific troponin-T promoter-driven control AAV9; AAV9-cTnT-METTL14, cardiac-specific troponin-T promoter-driven METTL14 overexpression AAV9. A-I/RI, acute myocardial ischemia-reperfusion injury. All data are expressed as means ± SD. (**a**, independent-sample t-test, two-sided; **b** and **c**, Two-way ANOVA followed by Tukey's post hoc test.) Source data are provided as a Source Data file.

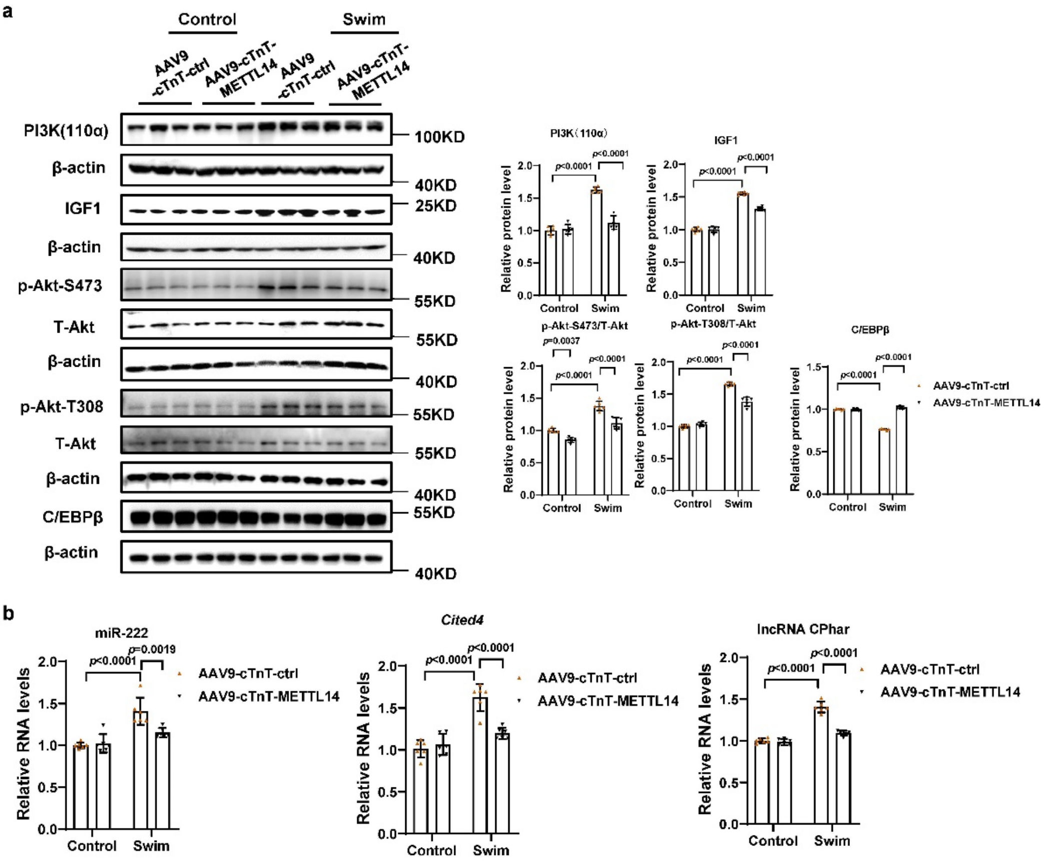

Supplementary Fig. 12: Expression of previously reported factors and signaling pathways known to be involved in exercise-induced cardiac hypertrophy. (**a**) Representative western blot and statistical data of IGF1/PI3K/Akt signaling and C/EBPβ expression levels in the mice hearts treated as indicated (n=6 mice/group). (**b**) qPCR analysis of miR-222, *Cited4*, and lncRNA CPhar expression levels in the hearts treated as indicated (n=6 mice/group). AAV9-cTnT-ctrl, cardiac-specific troponin-T promoter-driven control AAV9; AAV9-cTnT-METTL14, cardiac-specific troponin-T promoter-driven METTL14 overexpression AAV9. All data are expressed as means ± SD. (**a** and **b**, Two-way ANOVA followed by Tukey's post hoc test.) Source data are provided as a Source Data file.

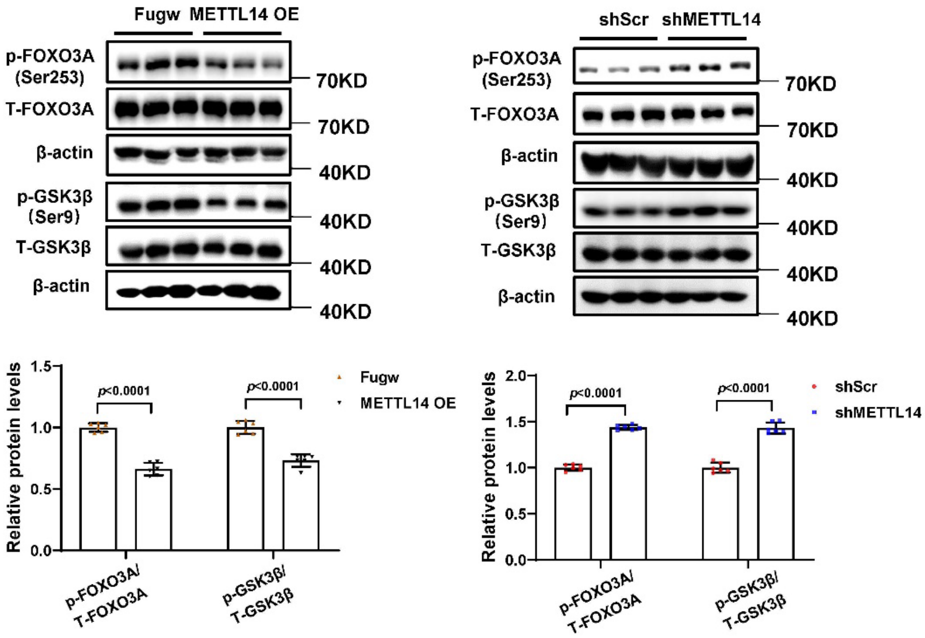

Supplementary Fig. 13: The phosphorylation levels of Akt substrates including FOXO3a, and GSK3β. Representative western blot and statistical data of phosphorylation levels of FOXO3a, and GSK3β in NRCMs with METTL14 knockdown or METTL14 overexpression (n=6 wells/group). Fugw, control without METTL14 overexpression; METTL14 OE, METTL14 overexpression; shScr, Scramble short hairpin RNA; shMETTL14, METTL14 short hairpin RNA to knockdown METTL14. All data are expressed as means ± SD. (Independent-sample t-test, two-sided.) Source data are provided as a Source Data file.

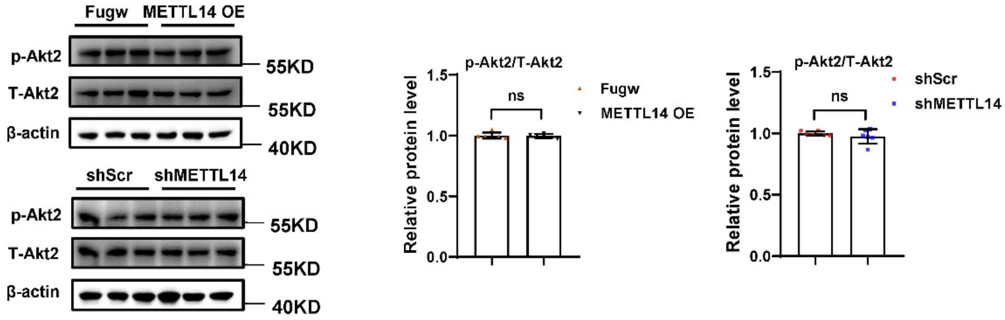

Supplementary Fig. 14: The phosphorylation levels of Akt2 in NRCMs with METTL14 knockdown or METTL14 overexpression (n=6 wells/group). Fugw, control without METTL14 overexpression; METTL14 OE, METTL14 overexpression; shScr, Scramble short hairpin RNA; shMETTL14, METTL14 short hairpin RNA to knockdown METTL14. ns, nonstatistically significant. All data are expressed as means ± SD. (Independent-sample t-test, two-sided.) Source data are provided as a Source Data file.

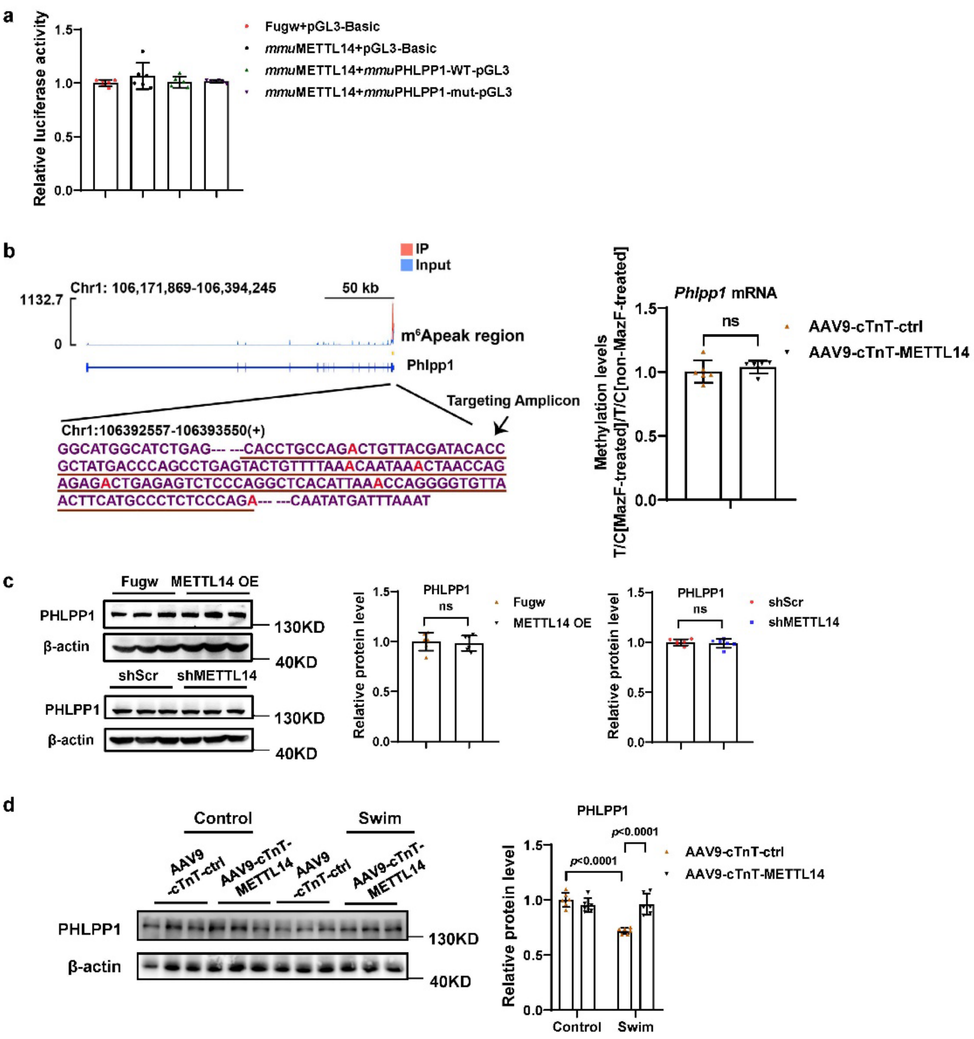

Supplementary Fig. 15: *Phlpp1* mRNA m^{6}A level and protein level were not affected by METTL14. (**a**) Relative luciferase activity of mouse PHLPP1 wild type (PHLPP1-WT-pGL3) or m^{6}A mutant (PHLPP1-mut-pGL3) reporter gene with or without mouse METTL14 overexpression (n=6/group). (**b**) The methylation levels of *Phlpp1* mRNA in mice hearts treated as indicated measured by MazF-qPCR (n=6 mice/group). (**c**) Representative western blot and statistical data of PHLPP1 in NRCMs with METTL14 knockdown or METTL14 overexpression (n=6 wells/group). (**d**) Representative western blot and statistical data of PHLPP1 expression level in the mice hearts treated as indicated (n=6 mice/group). Fugw, control without METTL14 overexpression; METTL14 OE, METTL14 overexpression; shScr, Scramble short hairpin RNA; shMETTL14, METTL14 short hairpin RNA to knockdown METTL14; AAV9-cTnT-ctrl, cardiac-specific troponin-T promoter-driven control AAV9; AAV9-cTnT-METTL14, cardiac-specific troponin-T promoter-driven METTL14 overexpression AAV9. ns, nonstatistically significant. All data are expressed as means ± SD. (**a**, one-way ANOVA followed by Dunnett T3 test; **b** and **c**, independent-sample t-test, two-sided; **d**, Two-way ANOVA followed by Tukey's post hoc test.) Source data are provided as a Source Data file.

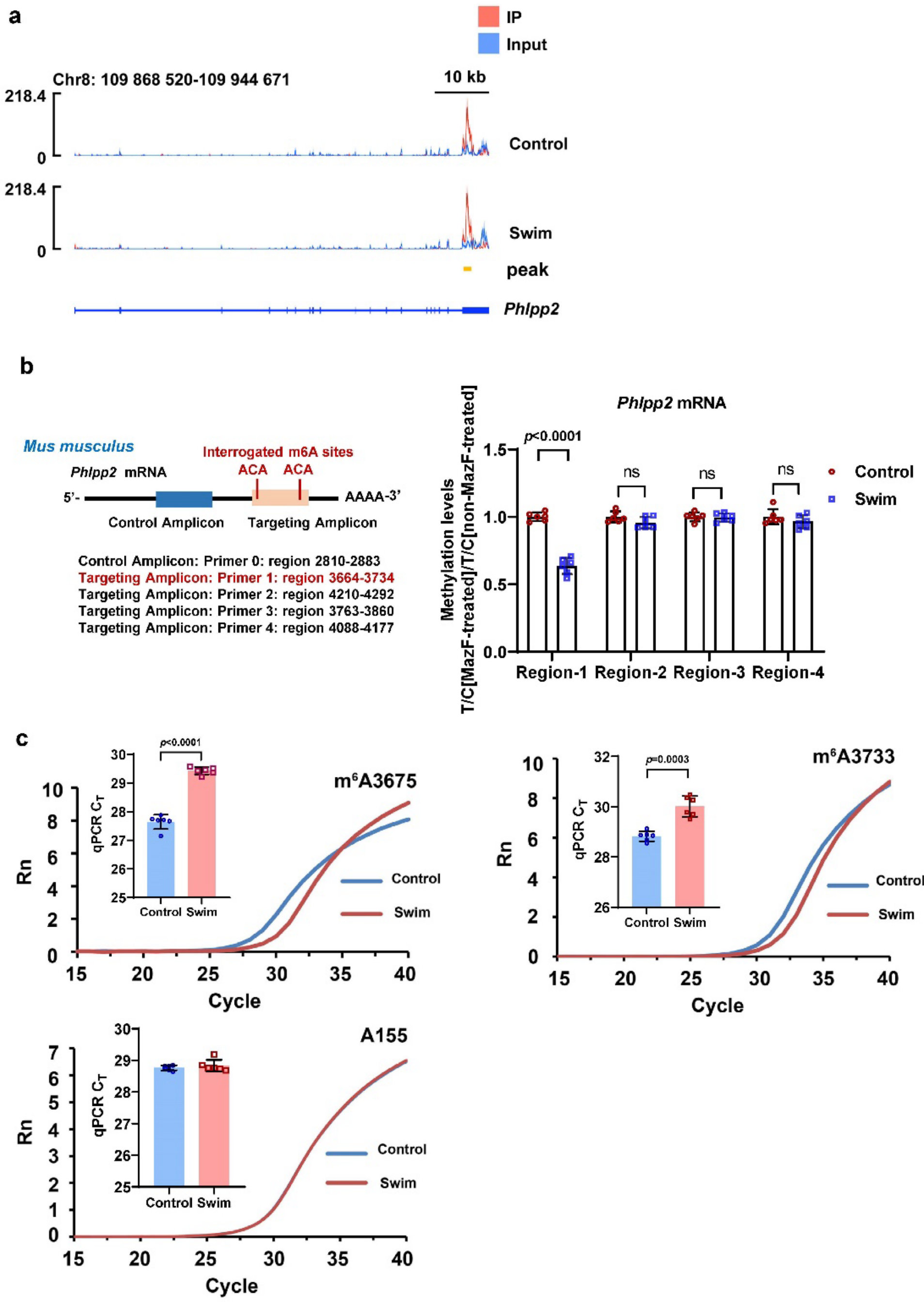

Supplementary Fig. 16: RNA m^{6}A modifications regulation in *Phlpp2* mRNA in response to exercise training in mouse heart. (**a**) Integrative genomics viewer (IGV) tracks revealing the results of meRIP-seq (Red) and RNA-seq (Blue) reads distributions in *Phlpp2* mRNA of the swim and control. Seq, sequencing. (**b**) MazF-qPCR identified the methylation modification region of *Phlpp2* mRNA which is specifically regulated in response to swimming training (n=6 mice/group). (**c**) Real-time fluorescence amplification curves and bar plot of the threshold cycle (C_T) of qPCR showing SELECT results for detecting m^{6}A3675, m^{6}A3733 and A155 sites (for input control) in *Phlpp2* mRNA between control and swim mice hearts (n=6 /group). ns, nonstatistically significant; All data are expressed as means ± SD. (**b** and **c**, independent-sample t-test, two-sided.) Source data are provided as a Source Data file.
