# Supplementary material for: METTL14 is required for exercise-induced cardiac hypertrophy and protects against myocardial ischemia-reperfusion injury
Source: Nat Commun. 2022 Nov 9;13:6762. doi: 10.1038/s41467-022-34434-y (PMC9646739; doi:10.1038/s41467-022-34434-y)
Supplement: Supplementary file 2 — Reporting Summary [file 41467_2022_34434_MOESM2_ESM.pdf]

nature portfolio

Corresponding author(s): Junjie Xiao

Last updated by author(s): Oct 9, 2022

Reporting Summary

Nature Portfolio wishes to improve the reproducibility of the work that we publish. This form provides structure for consistency and transparency in reporting. For further information on Nature Portfolio policies, see our Editorial Policies and the Editorial Policy Checklist.

Statistics

For all statistical analyses, confirm that the following items are present in the figure legend, table legend, main text, or Methods section.

n/a	Confirmed	
☐	☒	The exact sample size (*n*) for each experimental group/condition, given as a discrete number and unit of measurement
☐	☒	A statement on whether measurements were taken from distinct samples or whether the same sample was measured repeatedly
☐	☒	The statistical test(s) used AND whether they are one- or two-sided<br>*Only common tests should be described solely by name; describe more complex techniques in the Methods section.*
☒	☐	A description of all covariates tested
☐	☒	A description of any assumptions or corrections, such as tests of normality and adjustment for multiple comparisons
☐	☒	A full description of the statistical parameters including central tendency (e.g. means) or other basic estimates (e.g. regression coefficient) AND variation (e.g. standard deviation) or associated estimates of uncertainty (e.g. confidence intervals)
☐	☒	For null hypothesis testing, the test statistic (e.g. *F*, *t*, *r*) with confidence intervals, effect sizes, degrees of freedom and *P* value noted<br>*Give P values as exact values whenever suitable.*
☒	☐	For Bayesian analysis, information on the choice of priors and Markov chain Monte Carlo settings
☒	☐	For hierarchical and complex designs, identification of the appropriate level for tests and full reporting of outcomes
☒	☐	Estimates of effect sizes (e.g. Cohen's *d*, Pearson's *r*), indicating how they were calculated

Our web collection on statistics for biologists contains articles on many of the points above.

Software and code

Policy information about availability of computer code

Data collection

Echocardiography:
Vevo 2100 image system (VisualSonics Inc, Toronto, Ontario, Canada)
For brightfield and fluorescence microscopy:
Leica microscope (DM3000 LED, Germany)
Nikon Stereo Microscopes (SMZ18, Japan)
Zeiss Confocal microscope (LSM710, Germany)
Zeiss Confocal microscope (LSM880, Germany)
Leica microscope (DMi8, Germany)
For qPCR:
Real-Time PCR Detection System (Roche LightCycler480)
For Western blot:
ChemiDoc Imaging System (Tanon, 5200S)
ChemiDoc MP Imaging System (Bio-Rad, 17001402)
For meRIP-seq
Clean reads were mapped to the mouse genome (GRCm38) using HISAT2 (v2.1.0).
MeTDiff (v1.1.0) was used to call m6A peaks (HEPeak based on a Hidden Markov Model-based Exome Peak-finding algorithm) and differential m6A peaks and peak annotation was performed using chipseeker (v1.12.1). The putative differential m6A peaks were determined as the union region of the corresponding methylation peaks in the swim-trained or control samples which are predicted by HEPeak peak calling software. Then, MeTDiff models were used to determine the reads variation in a differential analysis also using a beta-binominal distribution. Finally, the statistical significance was determined using a likelihood ratio test. FPKM of each gene was calculated by Cufflinks (v2.2.1) using the sequencing reads from input samples. DESeq (v1.28.0) (2012) R package (v3.5.1) was used to find the differentially expressed genes.

Data analysis	Image J 1.50i was used for western blot grayscale analysis.<br>Microscopic images were analyzed by using ImageJ 1.50i.<br>All data are presented as mean ± SD using GraphPad Prism 8.0.<br>Statistical analyses were performed using SPSS 20.0 software (SPSS Inc., Chicago, IL) and GraphPad Prism 8.0.

For manuscripts utilizing custom algorithms or software that are central to the research but not yet described in published literature, software must be made available to editors and reviewers. We strongly encourage code deposition in a community repository (e.g. GitHub). See the Nature Portfolio guidelines for submitting code & software for further information.

Data

Policy information about availability of data

All manuscripts must include a data availability statement. This statement should provide the following information, where applicable:

- Accession codes, unique identifiers, or web links for publicly available datasets
- A description of any restrictions on data availability
- For clinical datasets or third party data, please ensure that the statement adheres to our policy

The data that support the findings of this study are available within the article and its Supplementary Information files. MeRIP-seq and its input RNA-seq data that support the findings of this study have been deposited in the BioSample database under BioProject ID PRJNA699979(https://www.ncbi.nlm.nih.gov/bioproject/PRJNA699979). Sequencing reads were mapped to the mouse genome reference GRCm38. A reporting summary for this article is available as a Supplementary Information file. Source data are provided with this paper.

Field-specific reporting

Please select the one below that is the best fit for your research. If you are not sure, read the appropriate sections before making your selection.

☒ Life sciences ☐ Behavioural & social sciences ☐ Ecological, evolutionary & environmental sciences

For a reference copy of the document with all sections, see nature.com/documents/nr-reporting-summary-flat.pdf

Life sciences study design

All studies must disclose on these points even when the disclosure is negative.

Sample size	No statistical method was performed to predetermine sample size. Instead, sample sizes were determined based on our prior experience with similar in in vitro and in vivo studies (PMID: 25863248, 30782145, 33092465, 33279721). The numbers of performed experiments were indicated in each figure legend.
Data exclusions	For in vivo I/R remodeling experiments, data from 4 mice with heart rates lower than 400 beats/min were excluded from further analyses for the reason that cardiac function measured by echocardiography should maintain the heart rates at 400-650 beats/min to ensure physiological relevance (PMID: 29351456).
Replication	Replication attempts were successful. All cell (including primary neonatal rat cardiomyocytes and cell line) experiments were repeated independently at least twice with similar results in repeated experiments. All in vivo experiments and its detections were performed using a minimum of 4 mice (exact numbers are repoted in figure legends).
Randomization	Animals were randomly assigned to experimental groups at the start of the study.<br>For cell culture experiments, individual wells were randomized into groups of treatment conditions.
Blinding	The group allocation of cell and mice was conducted in a blinded manner, investigators were also blinded to group assignments during data collection and analysis.<br>The experimental results analysis of molecular biology are obtained by quantitative methods, so we were not blinded to sample allocation.

Reporting for specific materials, systems and methods

We require information from authors about some types of materials, experimental systems and methods used in many studies. Here, indicate whether each material, system or method listed is relevant to your study. If you are not sure if a list item applies to your research, read the appropriate section before selecting a response.

Materials & experimental systems

n/a	Involved in the study
	☒ Antibodies
	☒ Eukaryotic cell lines
☒	Palaeontology and archaeology
	☒ Animals and other organisms
☒	Human research participants
☒	Clinical data
☒	Dual use research of concern

Methods

n/a	Involved in the study
☒	ChIP-seq
☒	Flow cytometry
☒	MRI-based neuroimaging

Antibodies

Antibodies used

The antibodies used for western blot:
1. Rabbit polyclonal anti-ZC3H13 Cat#ab70802, Lot: GR3235168-28, Abcam, 1:2000.
2. Rabbit monoclonal anti-C/EBPβ[E299] Cat#ab32358, Lot: GR3232277-8, clone#E299, Abcam, 1:1000.
3. Rabbit Polyclonal anti-PHLPP2 Cat# A18218, Lot: 1167720201, ABclonal, 1:1000.
4. Rabbit monoclonal anti-[KO Validated] active + pro Caspase-3 Cat# A19654, Lot: 4001330002, clone#ARC0133, ABclonal, 1:1000.
5. Rabbit monoclonal anti-Caspase-3 p12 Cat#A19664, Lot: 4000000143, clone#ARC0143, ABclonal, 1:1000.
6. Rabbit monoclonal anti-Bcl-2 Cat# A19693, Lot: 4000000173, clone#ARC0173, ABclonal, 1:1000.
7. Rabbit Polyclonal [KO Validated] anti-Bax Cat# A12009, Lot: 5500008806, ABclonal, 1:1000.
8. Rabbit Polyclonal anti-PHLPP1 Cat# A9542, Lot: 3521609005, ABclonal, 1:1000.
9. Rabbit polyclonal anti-RBM15B Cat# A9873, Lot: 0051800201, ABclonal, 1:1000.
10. Rabbit monoclonal anti-FOXO3a Cat#AF609, Lot: 031221210930, clone#75D8, Beyotime Biotechnology, 1:1000.
11. Rabbit polyclonal Phospho-FoxO3a (Ser253) Antibody Cat#9466, Lot:6, Cell Signaling Technology, 1:1000.
12. Rabbit polyclonal anti-GAPDH Cat# AP0063, Lot: 01201908, Bioworld Technology, 1:2000.
13. Rabbit monoclonal anti-METTL14(D8K8W) Cat# 51104, Lot: 1, clone#D8K8W, Cell Signaling Technology, 1:1000.
14. Rabbit monoclonal anti-METTL3(D2I6O) Cat#96391, Lot: 1, clone#D2I6O, Cell Signaling Technology, 1:1000.
15. Rabbit monoclonal anti-Phospho-Akt (Ser473) (D9E) XP Cat# 4060, Lot: 38, clone#D9E, Cell Signaling Technology, 1:1000.
16. Rabbit monoclonal anti-Phospho-Akt (Thr308) (D25E6) XP Cat#13038, Lot:18, clone# D25E6, Cell Signaling Technology, 1:1000.
17. Rabbit monoclonal anti-FTO (D2V1I) Cat# 45980, Lot: 1, clone#D2V1I, Cell Signaling Technology, 1:1000.
18. Rabbit monoclonal anti-ALKBH5 (E5Y7C) Cat# 80283, Lot: 1, clone#E5Y7C, Cell Signaling Technology, 1:1000.
19. Rabbit monoclonal anti-Phospho-Akt2 (Ser474) (D3H2) (Akt2 Specific) Cat# 8599, Lot: 2, clone#D3H2, Cell Signaling Technology, 1:1000.
20. Mouse Monoclonal anti-Akt2 (L79B2) Cat# 5239, Lot: 4, clone#L79B2, Cell Signaling Technology, 1:1000.
21. Rabbit monoclonal anti-Phospho-GSK-3β (Ser9) (D85E12) XP Cat# 5558, Lot: 10, clone#D85E12, Cell Signaling Technology, 1:1000.
22. Rabbit monoclonal anti-GSK-3β (D5C5Z) XP Cat# 12456, Lot: 8, clone#D5C5Z, Cell Signaling Technology, 1:1000.
23. Rabbit monoclonal anti-PI3 Kinase p110α (C73F8) Cat#4249, Lot: 11, clone#C73F8, Cell Signaling Technology, 1:1000.
24. Rabbit Polyclonal anti-WTAP Cat#10200-1-AP, Lot: 00048586, Proteintech, 1:1000.
25. Rabbit Polyclonal anti-AKT Cat# 10176-2-AP, Lot: 00099352, Proteintech, 1:1000.
26. Mouse Monoclonal anti-Beta Actin(2D4H5) Cat# 66009-1-Ig, Lot: 10004156, clone#2D4H5, Proteintech, 1:20000.
27. Rabbit polyclonal anti-IGF-1 Cat#28530-1-AP, Lot: 00085177, Proteintech, 1:1000.

The antibodies used for Immunofluorescence:
1. Rabbit Polyclonal anti-Phospho-Histone H3 (Ser10) Cat# PA5-17869, Lot: WC3216175A, Thermo Fisher Scientific, 1:200.
2. Rabbit monoclonal anti-Ki67 (SP6)-Proliferation Marker Cat# ab16667, Lot: GR3375640-18, clone#SP6, Abcam, 1:200.
3. Mouse monoclonal anti-alpha-Actinin (Sarcomeric)(EA-53) Cat# A7811, Lot: 0000120831, clone#EA-53, Sigma-Aldrich, 1:200.
4. Mouse Polyclonal anti-Cy3 AffiniPure Donkey Anti-Mouse IgG (H+L) Cat# 715-165-151, Lot: 155867, Jackson ImmunoResearch Labs, 1:200.
5. Rabbit Polyclonal anti-Alexa Fluor 488-AffiniPure Goat Anti-Rabbit IgG (H+L) Cat# 111-545-003, Lot: 146644, Jackson ImmunoResearch Labs, 1:200.
6. Mouse Polyclonal Alexa Fluor 488-conjugated Affinipure Goat Anti-Mouse IgG(H+L) Cat#115-545-003, Lot: 158218, Jackson ImmunoResearch Labs, 1:200.

The antibodies used for Immunoprecipitation:
1. Rabbit anti-m6A Cat# 202 003, Lot: 2-125, Synaptic Systems, 10μg/meRIP.
2. Normal Rabbit-IgG Cat#NI01, Lot: 3238429, Sigma-Aldrich, same μg to RIP antibody in each experiment.
3. Rabbit polyclonal anti-YTHDF1 Cat#17479-1-AP, Lot: 00102690, Proteintech, 4μg/RIP.
4. Rabbit polyclonal anti-METTL14 Cat#A8530, Lot: 5500003462, ABclonal, 4μg/RIP.

Validation

Validation was relied on the available data commercial websites for all antibodies.
The antibodies used for western blot:
1. Rabbit polyclonal anti-ZC3H13 Cat#ab70802, Lot: GR3235168-28, Abcam, 1:2000. (https://www.abcam.cn/zc3h13-antibody-ab70802.html) Abcam claims that this Rabbit polyclonal anti-ZC3H13 (Cat#ab70802) is suitable for WB applications in mouse.

2. Rabbit monoclonal anti-C/EBPβ[E299] Cat#ab32358, Lot: GR3232277-8, clone#E299, Abcam, 1:1000. (https://www.abcam.cn/cebp-beta-antibody-e299-c-terminal-ab32358.html) Abcam claims that this Rabbit monoclonal anti-C/EBPβ (Cat#ab32358) is suitable for WB applications in mouse.
3. Rabbit Polyclonal anti-PHLPP2 Cat# A18218, Lot: 1167720201, ABclonal, 1:1000. (https://abclonal.com.cn/catalog/A18218) ABclonal claims that this Rabbit Polyclonal anti-PHLPP2 (Cat# A18218) is suitable for WB applications in mouse and rat.
4. Rabbit monoclonal anti-[KO Validated] active + pro Caspase-3 Cat# A19654, Lot: 4001330002, clone#ARC0133, ABclonal, 1:1000. (https://abclonal.com.cn/catalog/A19654) ABclonal claims that this Rabbit monoclonal anti-[KO Validated] active + pro Caspase-3 Cat# A19654 is suitable for WB applications in mouse.
5. Rabbit monoclonal anti-Caspase-3 p12(ARC0143) Cat#A19664, Lot: 4000000143, clone#ARC0143, ABclonal, 1:1000. (https://abclonal.com.cn/catalog/A19664) ABclonal claims that this Rabbit monoclonal anti-Caspase-3 p12 (Cat#A19664) is suitable for WB applications in rat.
6. Rabbit monoclonal anti-Bcl-2(ARC0173) Cat# A19693, Lot: 4000000173, clone#ARC0173, ABclonal, 1:1000. (https://abclonal.com.cn/catalog/A19693) ABclonal claims that this Rabbit monoclonal anti-Bcl-2 (Cat# A19693) is suitable for WB applications in mouse and rat.
7. Rabbit Polyclonal [KO Validated] anti-Bax Cat# A12009, Lot: 5500008806, ABclonal, 1:1000. (https://abclonal.com.cn/catalog/A12009) ABclonal claims that this Rabbit Polyclonal [KO Validated] anti-Bax (Cat# A12009) is suitable for WB applications in mouse and rat.
8. Rabbit Polyclonal anti-PHLPP1 Cat# A9542, Lot: 3521609005, ABclonal, 1:1000. (https://abclonal.com.cn/catalog/A9542) ABclonal claims that this Rabbit Polyclonal anti-PHLPP1 (Cat# A9542) is suitable for WB applications in mouse and rat.
9. Rabbit polyclonal anti-RBM15B Cat# A9873, Lot: 0051800201, ABclonal, 1:1000. (https://abclonal.com.cn/catalog/A9873) ABclonal claims that this Rabbit polyclonal anti-RBM15B (Cat# A9873) is suitable for WB applications in mouse.
10. Rabbit monoclonal anti-FOXO3a(75D8) Cat#AF609, Lot: 031221210930, clone#75D8, Beyotime Biotechnology, 1:1000. (https://www.beyotime.com/product/AF609.htm) Beyotime claims that this Rabbit monoclonal anti-FOXO3a (Cat#AF609) is suitable for WB applications in mouse and rat.
11. Rabbit polyclonal Phospho-FoxO3a (Ser253) Antibody Cat#9466, Lot:6, Cell Signaling Technology, 1:1000. (https://www.cellsignal.cn/products/primary-antibodies/phospho-foxo3a-ser253-antibody/9466?site-search-type=Products&N=4294956287&Ntt=%239466&fromPage=plp&_requestid=596462) Cell Signaling Technology claims that this Rabbit polyclonal Phospho-FoxO3a (Ser253) Antibody (Cat#9466) is suitable for WB applications in mouse and rat.
12. Rabbit polyclonal anti-GAPDH Cat# AP0063, Lot: 01201908, Bioworld Technology, 1:2000. (https://www.bioworlde.com/Primary-Antibodies/46409.html) Bioworld claims that this Rabbit polyclonal anti-GAPDH (Cat# AP0063) is suitable for WB applications in rat cell.
13. Rabbit monoclonal anti-METTL14(D8K8W) Cat# 51104, Lot: 1, clone#D8K8W, Cell Signaling Technology, 1:1000. (https://www.cellsignal.cn/products/primary-antibodies/mettl14-d8k8w-rabbit-mab/51104?site-search-type=Products&N=4294956287&Ntt=mettl14&fromPage=plp) Cell Signaling Technology claims that this Rabbit monoclonal anti-METTL14 (Cat# 51104) is suitable for WB applications in mouse and rat.
14. Rabbit monoclonal anti-METTL3(D2I6O) Cat#96391, Lot: 1, clone#D2I6O, Cell Signaling Technology, 1:1000. (https://www.cellsignal.cn/products/primary-antibodies/mettl3-d2i6o-rabbit-mab/96391?site-search-type=Products&N=4294956287&Ntt=96391&fromPage=plp&_requestid=1843254) Cell Signaling Technology claims that this Rabbit monoclonal anti-METTL3 (Cat#96391) is suitable for WB applications in mouse and rat.
15. Rabbit monoclonal anti-Phospho-Akt (Ser473) (D9E) XP Cat# 4060, Lot: 38, clone#D9E, Cell Signaling Technology, 1:1000. (https://www.cellsignal.cn/products/primary-antibodies/phospho-akt-ser473-d9e-xp-rabbit-mab/4060?site-search-type=Products&N=4294956287&Ntt=4060&fromPage=plp&_requestid=1834821) Cell Signaling Technology claims that this Rabbit monoclonal anti-Phospho-Akt (Ser473) (D9E) XP (Cat# 4060) is suitable for WB applications in mouse and rat.
16. Rabbit monoclonal anti-Phospho-Akt (Thr308) (D25E6) XP Cat#13038, Lot:18, clone# D25E6, Cell Signaling Technology, 1:1000. (https://www.cellsignal.cn/products/primary-antibodies/phospho-akt-thr308-d25e6-xp-rabbit-mab/13038?site-search-type=Products&N=4294956287&Ntt=13038&fromPage=plp&_requestid=1844515) Cell Signaling Technology claims that this Rabbit monoclonal anti-Phospho-Akt (Thr308) (D25E6) XP (Cat#13038) is suitable for WB applications in mouse and rat.
17. Rabbit monoclonal anti-FTO (D2V1I) Cat# 45980, Lot: 1, clone#D2V1I, Cell Signaling Technology, 1:1000. (https://www.cellsignal.cn/products/primary-antibodies/fto-d2v1i-rabbit-mab/45980?site-search-type=Products&N=4294956287&Ntt=45980&fromPage=plp&_requestid=1844641) Cell Signaling Technology claims that this Rabbit monoclonal anti-FTO (D2V1I) (Cat# 45980) is suitable for WB applications in mouse and rat.
18. Rabbit monoclonal anti-ALKBH5 (E5Y7C) Cat# 80283, Lot: 1, clone#E5Y7C, Cell Signaling Technology, 1:1000. (https://www.cellsignal.cn/products/primary-antibodies/alkbh5-e5y7c-rabbit-mab/80283?site-search-type=Products&N=4294956287&Ntt=80283&fromPage=plp&_requestid=1844747) Cell Signaling Technology claims that this Rabbit monoclonal anti-ALKBH5 (E5Y7C) (Cat# 80283) is suitable for WB applications in mouse and rat.
19. Rabbit monoclonal anti-Phospho-Akt2 (Ser474) (D3H2) (Akt2 Specific) Cat# 8599, Lot: 2, clone#D3H2, Cell Signaling Technology, 1:1000. (https://www.cellsignal.cn/products/primary-antibodies/phospho-akt2-ser474-d3h2-rabbit-mab-akt2-specific/8599?site-search-type=Products&N=4294956287&Ntt=8599&fromPage=plp&_requestid=1844821) Cell Signaling Technology claims that this Rabbit monoclonal anti-Phospho-Akt2 (Ser474) (D3H2) (Akt2 Specific) (Cat# 8599) is suitable for WB applications in rat.
20. Mouse Monoclonal anti-Akt2 (L79B2) Cat# 5239, Lot: 4, clone#L79B2, Cell Signaling Technology, 1:1000. (https://www.cellsignal.cn/products/primary-antibodies/akt2-l79b2-mouse-mab/5239?site-search-type=Products&N=4294956287&Ntt=5239&fromPage=plp&_requestid=1844979) Cell Signaling Technology claims that this Mouse Monoclonal anti-Akt2 (L79B2) (Cat# 5239) is suitable for WB applications in rat.
21. Rabbit monoclonal anti-Phospho-GSK-3β (Ser9) (D85E12) XP Cat# 5558, Lot: 10, clone#D85E12, Cell Signaling Technology, 1:1000. (https://www.cellsignal.cn/products/primary-antibodies/phospho-gsk-3b-ser9-d85e12-xp-rabbit-mab/5558?site-search-type=Products&N=4294956287&Ntt=5558&fromPage=plp&_requestid=1845081) Cell Signaling Technology claims that this Rabbit monoclonal anti-Phospho-GSK-3β (Ser9) (D85E12) XP (Cat# 5558) is suitable for WB applications in rat.
22. Rabbit monoclonal anti-GSK-3β (D5C5Z) XP Cat# 12456, Lot: 8, clone#D5C5Z, Cell Signaling Technology, 1:1000. (https://www.cellsignal.cn/products/primary-antibodies/gsk-3b-d5c5z-xp-rabbit-mab/12456?site-search-type=Products&N=4294956287&Ntt=12456&fromPage=plp&_requestid=1845169) Cell Signaling Technology claims that this Rabbit monoclonal anti-GSK-3β (D5C5Z) XP (Cat# 12456) is suitable for WB applications in rat.
23. Rabbit monoclonal anti-PI3 Kinase p110α (C73F8) Cat#4249, Lot: 11, clone#C73F8, Cell Signaling Technology, 1:1000. (https://www.cellsignal.cn/products/primary-antibodies/pi3-kinase-p110a-c73f8-rabbit-mab/4249?site-search-

type=Products&N=4294956287&Ntt=4249&fromPage=plp&_requestid=1845299) Cell Signaling Technology claims that this Rabbit monoclonal anti-PI3 Kinase p110α (C73F8) (Cat#4249) is suitable for WB applications in mouse.
24. Rabbit Polyclonal anti-WTAP Cat#10200-1-AP, Lot: 00048586, Proteintech, 1:1000. (https://www.ptgcn.com/products/WTAP-Antibody-10200-1-AP.htm) Proteintech Group claims that this Rabbit Polyclonal anti-WTAP (Cat#10200-1-AP) is suitable for WB applications in mouse.
25. Rabbit Polyclonal anti-AKT Cat# 10176-2-AP, Lot: 00099352, Proteintech, 1:1000. (https://www.ptgcn.com/products/AKT-Antibody-10176-2-AP.htm) Proteintech Group claims that this Rabbit Polyclonal anti-AKT (Cat# 10176-2-AP) is suitable for WB applications in mouse and rat.
26. Mouse Monoclonal anti-Beta Actin Cat# 66009-1-Ig, Lot: 10004156, clone#2D4H5, Proteintech, 1:20000. (https://www.ptgcn.com/products/Pan-Actin-Antibody-66009-1-Ig.htm) Proteintech Group claims that this Mouse Monoclonal anti-Beta Actin (Cat# 66009-1-Ig) is suitable for WB applications in mouse and rat.
27. Rabbit polyclonal anti-IGF-1 Cat#28530-1-AP, Lot: 00085177, Proteintech, 1:1000. (https://www.ptgcn.com/products/IGF1-Antibody-28530-1-AP.htm#product-information) Proteintech Group claims that this Rabbit polyclonal anti-IGF-1 (Cat#28530-1-AP) is suitable for WB applications in mouse.

The antibodies used for Immunofluorescence:
1. Rabbit Polyclonal anti-Phospho-Histone H3 (Ser10) Cat# PA5-17869, Lot: WC3216175A, Thermo Fisher Scientific, 1:200. (https://www.thermofisher.cn/cn/zh/antibody/product/Phospho-Histone-H3-Ser10-Antibody-Polyclonal/PA5-17869) Thermo Fisher Scientific claims that this Rabbit Polyclonal anti-Phospho-Histone H3 (Ser10) (Cat# PA5-17869) is suitable for IF applications in mouse and rat.
2. Rabbit monoclonal anti-Ki67 (SP6) Cat# ab16667, Lot: GR3375640-18, clone#SP6, Abcam, 1:200. (https://www.abcam.cn/ki67-antibody-sp6-ab16667.html) Abcam claims that this Rabbit monoclonal anti-Ki67 antibody [SP6] (Cat# ab16667) is suitable for IF applications in mouse and rat.
3. Mouse monoclonal anti-alpha-Actinin (Sarcomeric)(EA-53) Cat# A7811, Lot: 0000120831, clone#EA-53, Sigma-Aldrich, 1:200. (https://www.sigmaaldrich.cn/CN/zh/product/sigma/a7811) Sigma-Aldrich claims that this Mouse monoclonal anti-alpha-Actinin (Sarcomeric) (Cat# A7811) is suitable for IF applications in mouse and rat.
4. Mouse Polyclonal anti-Cy3 AffiniPure Donkey Anti-Mouse IgG (H+L) Cat# 715-165-151, Lot: 155867, Jackson ImmunoResearch Labs, 1:200. (https://www.jacksonimmuno.com/catalog/products/715-165-151).
5. Rabbit Polyclonal anti-Alexa Fluor 488-AffiniPure Goat Anti-Rabbit IgG (H+L) Cat# 111-545-003, Lot: 146644, Jackson ImmunoResearch Labs, 1:200. (https://www.jacksonimmuno.com/catalog/products/111-545-003)
6. Mouse Polyclonal Alexa Fluor 488-conjugated Affinipure Goat Anti-Mouse IgG(H+L) Cat#115-545-003, Lot: 158218, Jackson ImmunoResearch Labs, 1:200. (https://www.jacksonimmuno.com/catalog/products/115-545-003)

The antibodies used for Immunoprecipitation:
1. Rabbit anti-m6A Cat# 202 003, Lot: 2-125, Synaptic Systems, 10μg/meRIP. (https://www.sysy.com/product/202003#list) Synaptic Systems claims that this Rabbit anti-m6A (Cat# 202 003) is suitable for IP applications.
2. Normal Rabbit-IgG Cat#NI01, Lot: 3238429, Sigma-Aldrich, same μg to RIP antibody in each experiment. (https://www.sigmaaldrich.cn/CN/zh/product/mm/ni01) Sigma-Aldrich claims that this Normal Rabbit-IgG (Cat#NI01) is suitable for IP applications.
3. Rabbit polyclonal anti-YTHDF1 Cat#17479-1-AP, Lot: 00102690, Proteintech, 4μg/RIP. (https://www.ptgcn.com/products/YTHDF1-Antibody-17479-1-AP.htm) Proteintech Group claims that this Rabbit polyclonal anti-YTHDF1 (Cat#17479-1-AP) is suitable for IP applications in in mouse and rat.
4. Rabbit polyclonal anti-METTL14 Cat#A8530, Lot: 5500003462, ABclonal, 4μg/RIP. (https://abclonal.com.cn/catalog/A8530) ABclonal claims that this Rabbit polyclonal anti-METTL14 (Cat#A8530) is suitable for IP applications in mouse and rat.

Eukaryotic cell lines

Policy information about cell lines

Cell line source(s)	Rat H9C2 cardiomyocytes (#GNR5) was purchased from Stem Cell Bank, Chinese Academy of Sciences. HEK293T cells was kindly gifted by Dr. Qiurong Ding at Chinese Academy of Sciences, Shanghai, China, originally purchased from Stem Cell Bank, Chinese Academy of Sciences.
Authentication	We authenicated the species of H9C2 by PCR assays with species-specific primers, and verified the species of H9C2 is Rat, no cross contamination with human, mouse and hamster species. HEK293T Cell line was not authenicated.
Mycoplasma contamination	Cells were not tested for mycoplasma contamination.
Commonly misidentified lines (See ICLAC register)	No commonly misidentified cell lines were used.

Animals and other organisms

Policy information about studies involving animals; ARRIVE guidelines recommended for reporting animal research

Laboratory animals	1. mouse, male C57BL/6J, 8-9 weeks, housed in a barrier facility on a 12 h light/dark cycle at 22-24℃ and 45-55% humidity with access to food (cat#1010083, Xietong Shengwu, Nanjing,China) and water ad libitum.<br>2. Sprague-Dawley (SD) rats, male and female, 1-3-day old.
Wild animals	Our study did not involve wild animals.

Field-collected samples	Our study did not involve field-collections.
Ethics oversight	All experiments of animal were in accordance with the guidelines on the use and care of laboratory animals for biomedical research published by the National Institutes of Health (No. 85-23, revised 1996), and approved by the committee on the Ethics of Animal Experiments of Shanghai University (No.2020-043).

Note that full information on the approval of the study protocol must also be provided in the manuscript.
